# Numerical data on heat flux of a novel controlled-temperature double skin façade

**DOI:** 10.1016/j.dib.2021.107034

**Published:** 2021-04-20

**Authors:** Loucas Georgiou, Angeliki Kylili, Paris A. Fokaides

**Affiliations:** a Frederick Research Center, Cyprus; b Frederick University, School of Engineering, Cyprus

**Keywords:** Double skin façade (DSF), Heat flux, Computational fluid dynamics (CFD), Simulation, Building, Orientation, Climatic conditions

## Abstract

Hourly heat flux for variant boundary conditions of a novel controlled-temperature double skin façade (DSF) building element in a two- dimensional time- dependent study was determined. The building element is subjected to boundary conditions, characterizing different orientations (azimuth 0°, 90°, 180°, 270°) and climatic conditions of the four seasons. This data article provides detailed numerical data on the hourly heat flux, temperatures attained at the exterior and within the building element for six different geometries and for the variant boundary conditions under study. The external boundary conditions were determined with the use of the PVGIS tool, corrected in accordance to the sol-air temperature equation. The numerical simulation studies were performed with the use of the computational fluid dynamics (CFD) tool Comsol Multiphysics [2].

**Specifications Table**

**Table:** 

Subject	Energy Engineering, Building physics
Specific subject area	Heat transfer, Finite Elements Modeling (FEM), Heat flux, double skin façade (DSF), Boundary conditions
Type of data	Tables; Figures
How data were acquired	Solar radiation tool (PVGIS) [1] for boundary conditions
	Finite elements numerical calculation model (COMSOL Multiphysics) for heat flux
Data format	Analysed and processed output data
Parameters for data collection	The geometric parameters, which have been used as input for the development of the numerical simulation models, were obtained using experimental building elements. The international standard EN 10456: 2007 has provided the building materials’ thermophysical properties [3], while for those materials not included in the standard, their properties were obtained from laboratory tests.
	The temperature profiles, which were imposed on the exterior surfaces of the masonry to represent the variant climatic conditions under investigation, were obtained with the use of PVGIS [1]. Accordingly, data from the PVGIS tool [1] was obtained to be representative of the different orientations and seasons
Description of data collection	The values used for the development of the geometric models of the DSF building elements were based on actual experimental building elements. For the materials, whose thermophysical properties were acquired experimentally, their properties were measured based on the analysis of the temperature response of each material to heat flow impulses with the use of a measuring instrument for direct measurement of heat transfer properties.
	The PVGIS tool [1] was employed for the acquirement of climatic data, which was used as input for the definition of the external boundary conditions of the simulation models. The climatic data extracted was for the calendar months January (winter), April (spring), July (summer), and October (autumn) and for the orientations azimuth 0°, 90°, 180° and 270°. The acquired data were corrected using the sol-air temperature equation.
	The heat flux data was extracted from the FEM tool, based on the numerical simulation studies conducted.
Data source location	Nicosia, Cyprus, 35.18° N, 33.37°E
Data accessibility	https://data.mendeley.com/datasets/dfj4f7fww4/3

**Value of the Data**

• The data provided in this work presents the variability of heat flux for a building wall incorporating a DSF under variant external boundary conditions.
• The data can be useful for both new and existing buildings, where the application of a DSF aims to the reduction of heat losses through the building wall and the improvement of the building's thermal performance.
• The dataset can support researchers by demonstrating the methodology for the development of novel building elements and the definition the optimal design for application, given the key objective is improving the overall energy performance of the built environment.

## 1 Data Description

Figures and tables provided in this work represent reference figures (Fig. 1) or present summary information (Fig. 2, Fig. 3, Fig. 4, Fig. 5 and Table 1, Table 2, Table 3), whereas figures and tables provided in the supplementary material in Mendeley Data include more analytical data (Reference Fig. 1, Fig. 2, Fig. 3, Fig. 4, Fig. 5, Figures A1-A6, Figures B1-B24, Tables A1-A6).

**Fig. 1:**
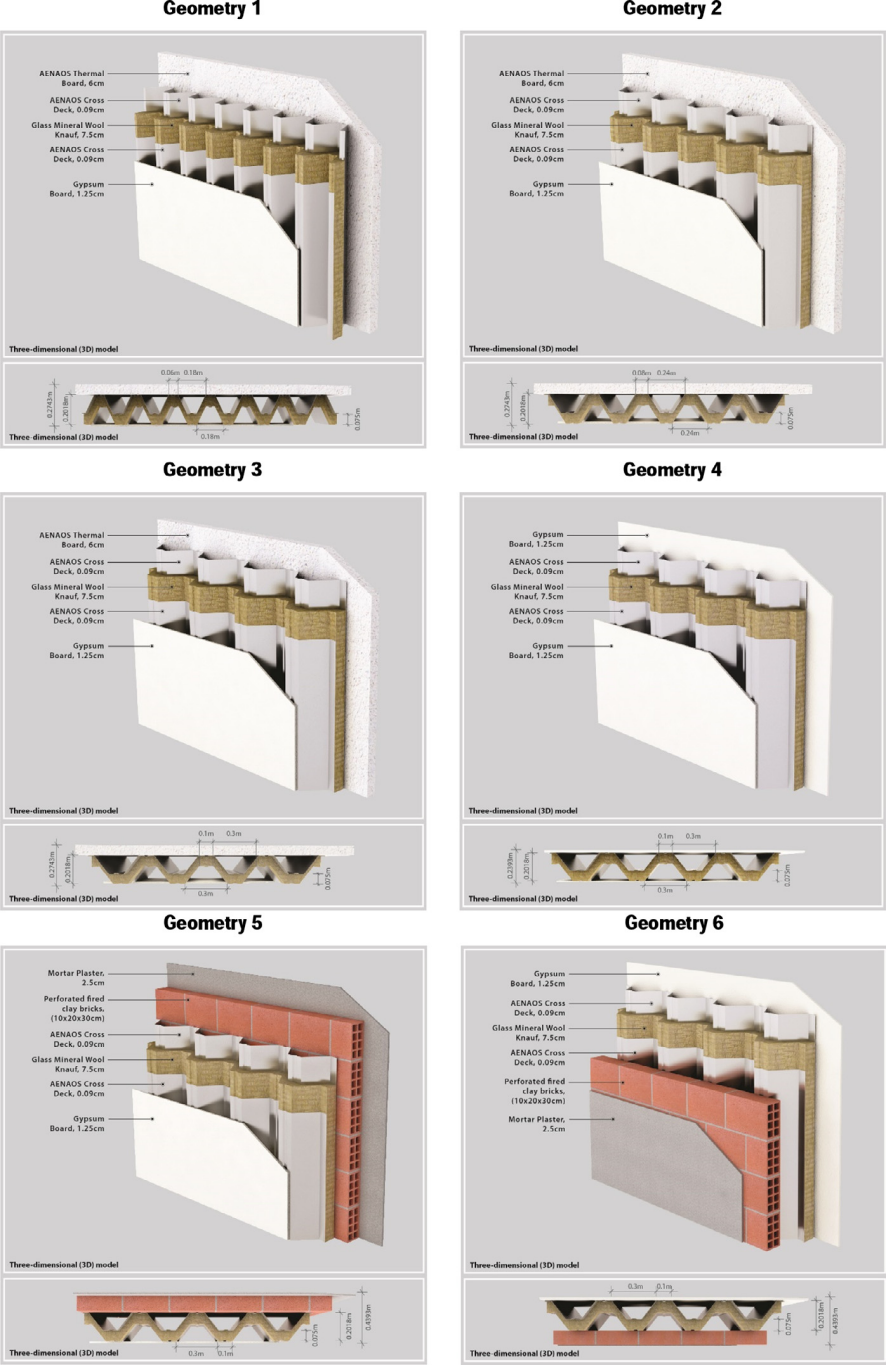
Three- dimensional designs of the geometries of the novel controlled-temperature building element. Geometry 1, Geometry 2, Geometry 3, Geometry 4, Geometry 5, Geometry 6.

**Fig. 2:**
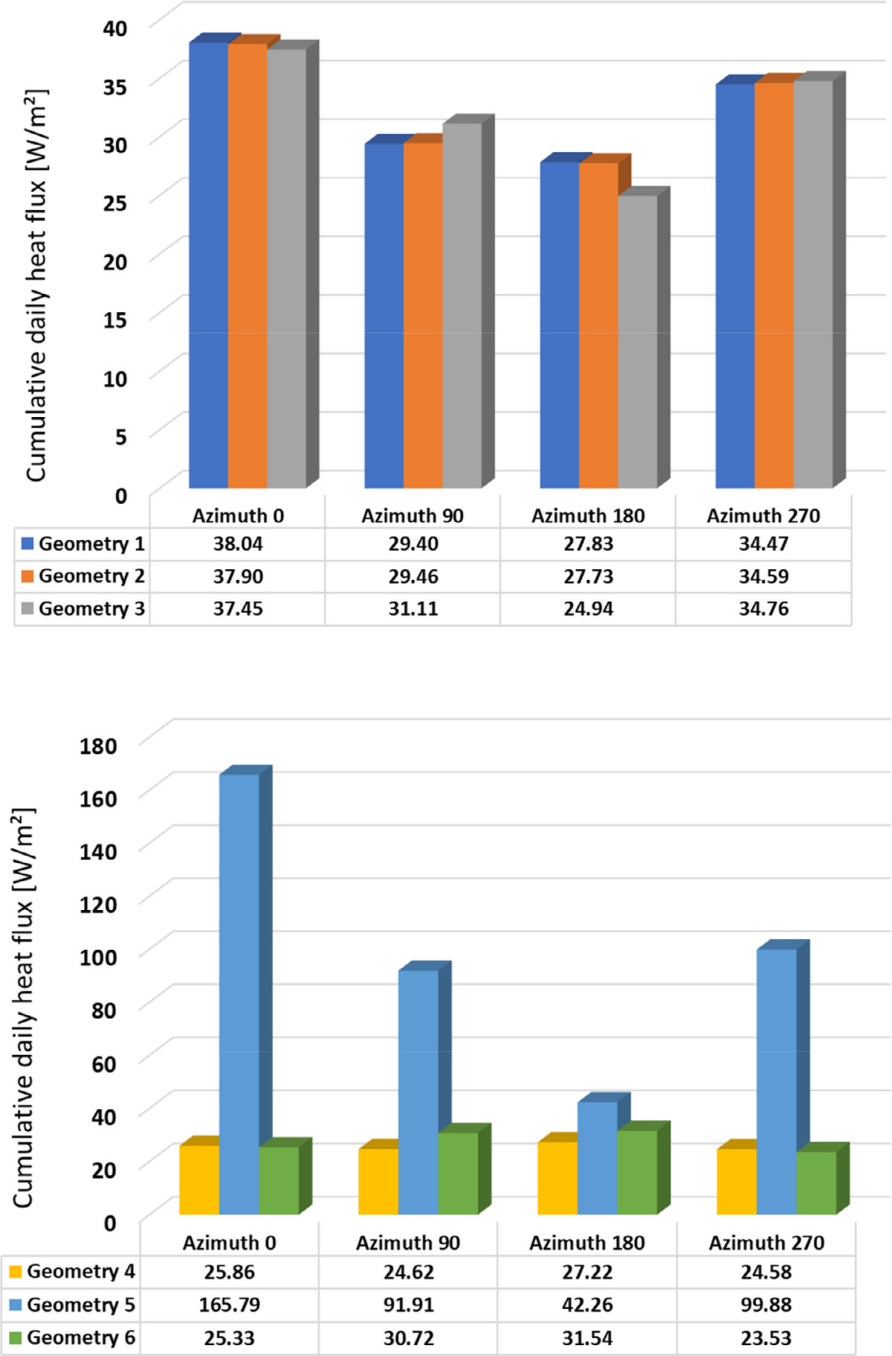
Cumulative daily heat flux – Winter – Geometries 1-3 (above); 4-6 (below).

**Fig. 3:**
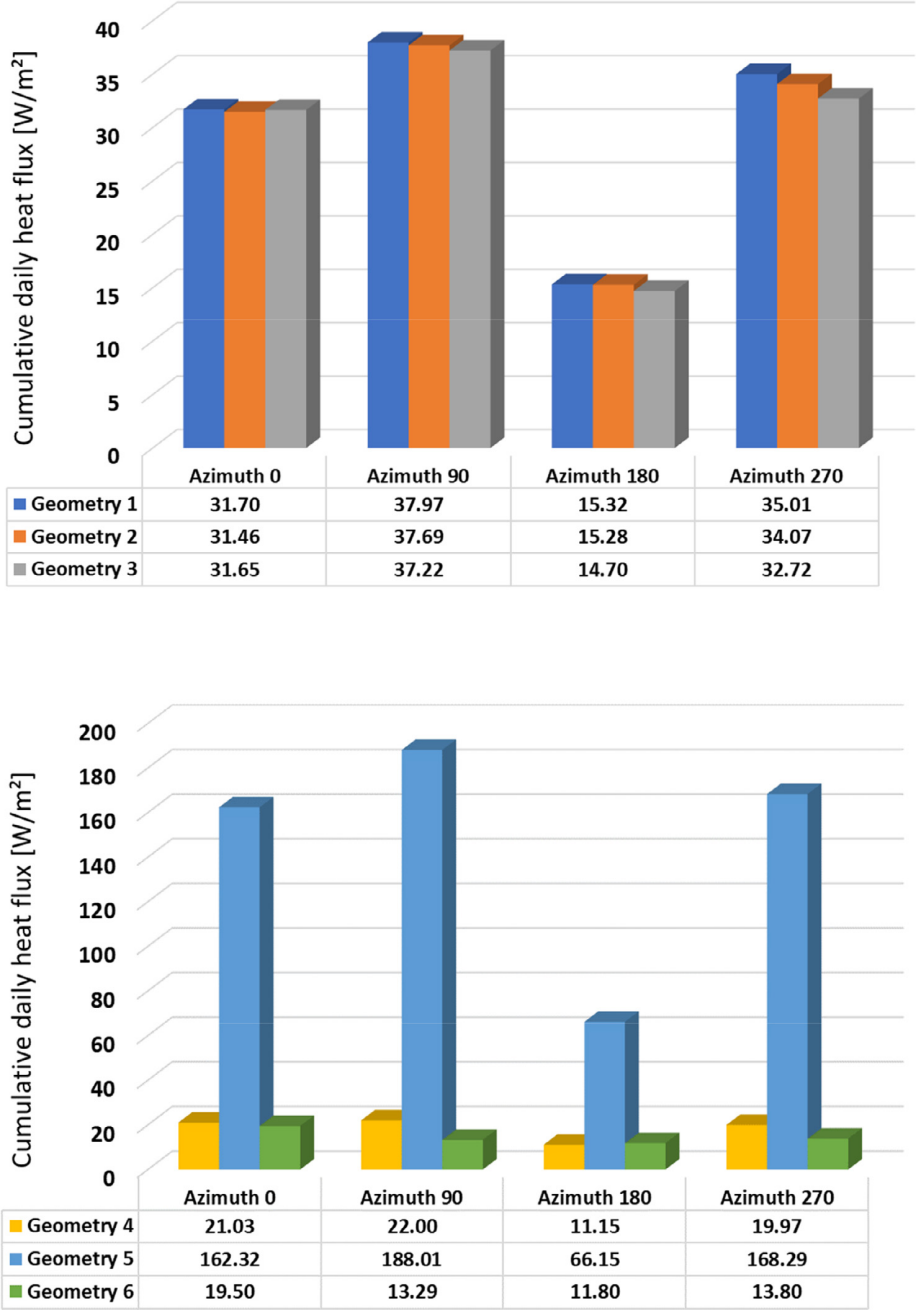
Cumulative daily heat flux – Spring – Geometries 1-3 (above); 4-6 (below).

**Fig. 4:**
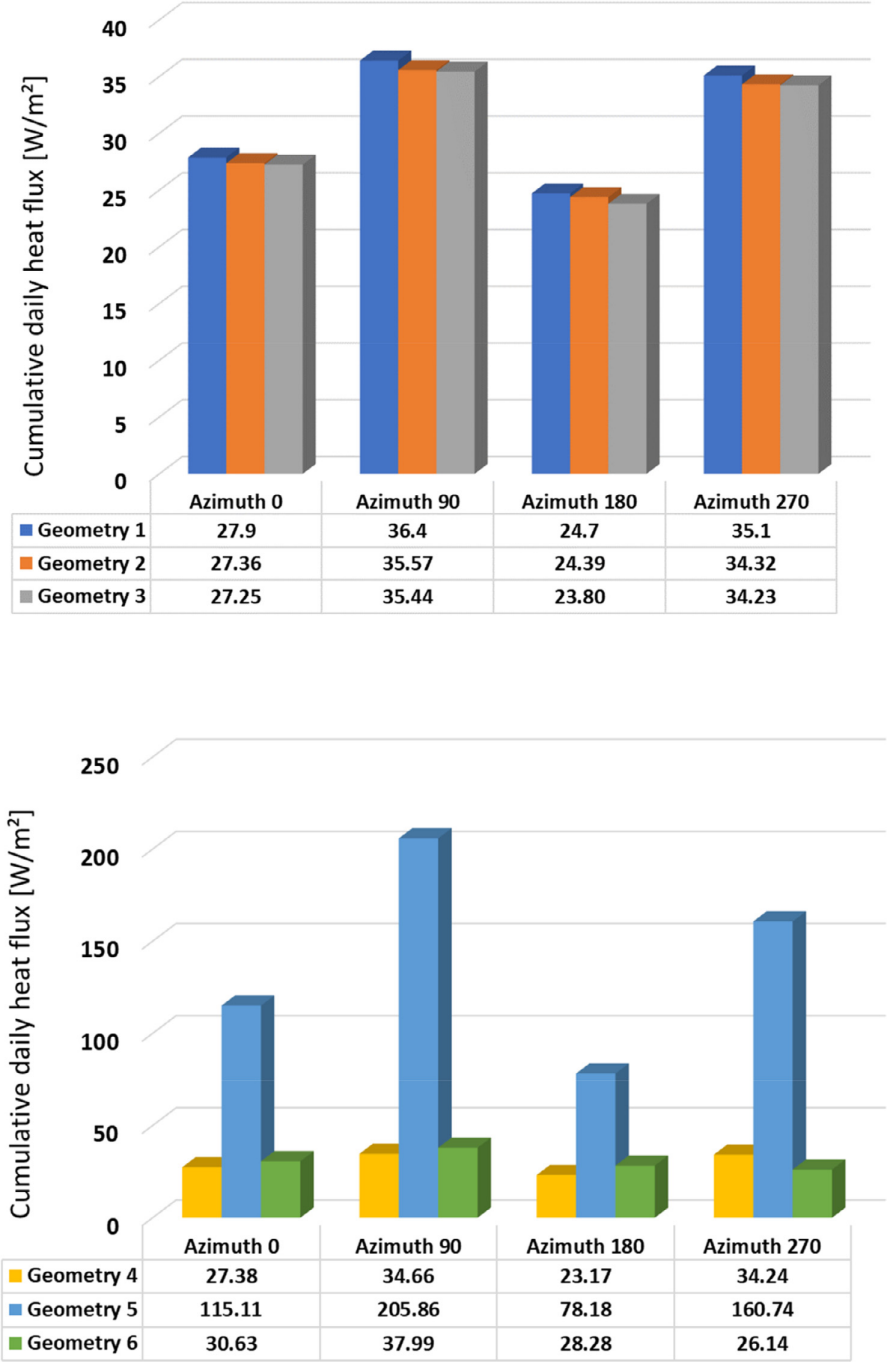
Cumulative daily heat flux – Summer – Geometries 1-3 (above); 4-6 (below).

**Fig. 5:**
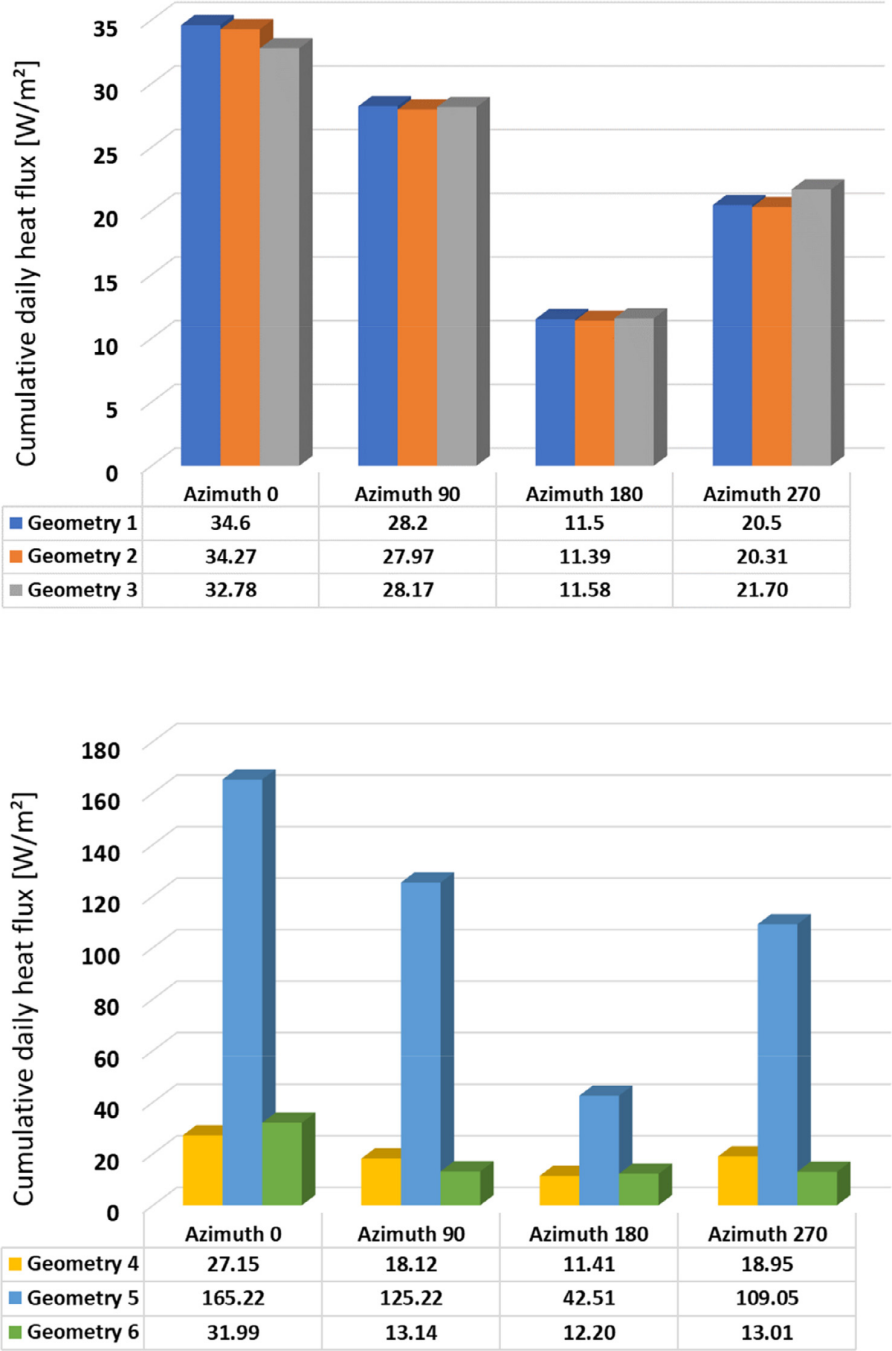
Cumulative daily heat flux – Autumn – Geometries 1-3 (above); 4-6 (below).

**Table 1:** Cumulative daily heat flux [$W/m^{2}$] according to orientation and season.

	Winter	Spring	Summer	Autumn
Azimuth 0°				

Geometry 1	38.04	31.70	27.90	34.60
Geometry 2	37.90	31.46	27.36	34.27
Geometry 3	37.45	31.65	27.25	32.78
Geometry 4	25.86	21.03	27.38	27.15
Geometry 5	165.79	162.32	115.11	165.22
Geometry 6	25.33	19.50	30.63	31.99

Azimuth 90°				

Geometry 1	29.40	37.97	36.40	28.20
Geometry 2	29.46	37.69	35.57	27.97
Geometry 3	31.11	37.22	35.44	28.17
Geometry 4	24.62	22.00	34.66	18.12
Geometry 5	91.91	188.01	205.86	125.22
Geometry 6	30.72	13.29	37.99	13.14

Azimuth 180°				

Geometry 1	27.83	15.32	24.70	11.50
Geometry 2	27.73	15.28	24.39	11.39
Geometry 3	24.94	14.70	23.80	11.58
Geometry 4	27.22	11.15	23.17	11.41
Geometry 5	42.26	66.15	78.18	42.51
Geometry 6	31.54	11.80	28.28	12.20

Azimuth 270°				

Geometry 1	34.7	35.01	35.10	20.50
Geometry 2	34.59	34.07	34.32	20.31
Geometry 3	34.76	32.72	34.23	21.70
Geometry 4	24.58	19.97	34.24	18.95
Geometry 5	99.88	168.29	160.74	109.05
Geometry 6	23.53	13.80	26.14	13.01

Fig. 1 presents the building elements which were investigated in terms of this work are provided. The dataset is comprised of tables of hourly heat flux of six (6) different designs of a novel controlled-temperature DSF building element (AENAOS) for a 24- hour time period (Tables A1 – A6). The data is differentiated into the four different seasons of the year (winter, spring, summer, autumn) and four different orientations (azimuth 0°, 90°, 180°, 270°). The hourly heat flux of the six designs per season and orientation is also shown graphically in Figures A1 – A6.

The temperatures obtained at three different points of the building elements under investigation are shown in Figures B1 – B24. For each of the investigated designs, Point 1 is located at the external surface of the building element; Point 2 is located within the thermal board and Point 3 is located within the glass mineral wool (points’ location indicated in Reference Figures 7-12).

The summary of the cumulative daily heat flux are provided in Table 1 and illustrated in Fig. 2, Fig. 3, Fig. 4, Fig. 5. This data is the sum of absolute hourly values and are distinguished by building element design, season, and orientation. In the summary table, heat flux values are highlighted in red and green colour, where red indicates the highest values and green indicates the lowest values.

## 2 Experimental Design, Materials and Methods

The calculation procedure, based on two- dimensional time- dependent finite element numerical modeling which solves the equation of heat transfer for a novel DSF building element (AENAOS), has been performed by the followed steps:

1. Development of the mathematical model and calculation algorithm. A physics controlled mesh and extremely fine size elements were used to discretize the model, and a time step of 0, 1, 72 [hr] range.
2. Development of the building element designs, distinguished by variations in the geometry of the trapezoidal sheet and the thickness of the materials. The investigated geometries of the novel controlled-temperature building element, illustrated in Figure 1, take into consideration existing construction solutions and restrictions related to the practical application of the building element.
3. Definition of the materials’ thermophysical properties retrieved from the international standard EN 10456:2007 and laboratory tests [3] (Table 2).

**Table 2:** Thermophysical properties of the materials used as input in the numerical simulation study of novel double- skin façade (DSF) controlled-temperature building element.

Material	Density [kg/m^3^]	Thermal Conductivity [W/(m∙K)]	Heat Capacity [J(Kg∙K)]	Thickness [cm]
Glass Mineral Wool	50	0.040	1030	7.50
Gypsum Board	664	0.190	1090	1.25
Mortar Plaster	700	1.000	1000	2.50
Perforated Fired Clay Brick (Clay Material)	880	0.400	900	20.00
Perforated Fired Clay Brick (Air Holes 5 × 5 [cm])	1.23	0.025	1008	20.00
AENAOS Cross Deck	7850	44.500	475	0.09

**Table 3:** Calculated hourly temperature values used as external boundary temperature (°C) in the numerical simulation study of novel double- skin façade (DSF) controlled-temperature building element.

Season/ Calculated External Temperature [°C]	Winter				Spring				Summer				Autumn			
Time [hr]	Azimuth [°]															

	0	90	180	270	0	90	180	270	0	90	180	270	0	90	180	270
1	12,01	12,01	12,01	12,01	15,66	15,66	15,66	15,66	25,84	25,84	25,84	25,84	23,74	23,74	23,74	23,74
2	12,01	12,01	12,01	12,01	15,57	15,57	15,57	15,57	25,52	25,52	25,52	25,52	23,66	23,66	23,66	23,66
3	12,01	12,01	12,01	12,01	15,47	15,47	15,47	15,47	25,20	25,20	25,20	25,20	23,58	23,58	23,58	23,58
4	12,02	12,02	12,02	12,02	15,38	15,38	15,38	15,38	25,07	25,07	25,60	26,12	23,49	23,49	23,49	23,49
5	12,14	12,14	12,14	12,14	16,96	16,96	18,29	25,61	27,17	27,17	31,48	40,21	23,98	23,79	23,79	24,72
6	12,66	12,32	12,32	12,25	20,30	18,50	18,78	35,59	28,55	28,55	32,77	49,87	30,15	24,97	24,97	37,42
7	20,63	13,41	13,41	13,12	25,58	19,93	19,93	39,18	30,93	29,66	30,99	52,21	35,61	25,87	25,87	40,78
8	24,84	14,52	14,52	23,30	30,45	20,91	20,91	38,88	35,47	30,18	30,18	49,86	41,08	26,92	26,92	40,84
9	30,47	15,30	15,30	24,32	33,99	22,07	22,07	34,81	38,61	30,48	30,48	45,14	44,94	28,24	28,24	37,89
10	32,67	15,68	15,68	24,46	35,82	22,38	22,38	28,97	40,59	30,82	30,82	38,66	46,92	28,97	28,97	33,10
11	31,93	17,93	15,69	20,89	35,26	27,80	23,05	23,05	40,92	35,72	30,98	30,98	44,83	34,60	29,59	29,59
12	30,29	21,87	15,76	15,91	33,68	33,08	22,74	22,74	39,82	42,78	31,11	31,11	42,51	38,06	28,83	28,83
13	27,59	24,38	15,84	15,98	30,57	36,62	22,06	22,06	37,24	47,31	31,26	31,26	40,44	41,56	28,79	28,79
14	24,57	25,38	15,09	15,65	27,61	39,27	21,83	21,83	34,13	51,15	30,89	30,89	35,30	40,48	27,86	27,86
15	18,25	20,67	14,04	14,90	23,69	40,13	20,93	20,93	30,26	53,52	32,88	30,26	30,02	35,63	26,68	26,68
16	13,26	13,26	13,26	13,85	20,11	34,18	21,13	20,01	29,83	50,48	34,60	29,83	25,35	25,35	25,35	25,35
17	13,20	13,20	13,20	13,20	18,13	18,20	18,16	18,13	28,72	40,99	33,19	28,72	24,98	24,98	24,98	24,98
18	13,14	13,14	13,14	13,14	17,46	17,46	17,46	17,46	27,60	27,60	27,60	27,60	24,62	24,62	24,62	24,62
19	13,07	13,07	13,07	13,07	16,80	16,80	16,80	16,80	27,24	27,24	27,24	27,24	24,26	24,26	24,26	24,26
20	13,07	13,07	13,07	13,07	16,52	16,52	16,52	16,52	26,83	26,83	26,83	26,83	24,00	24,00	24,00	24,00
21	13,07	13,07	13,07	13,07	16,24	16,24	16,24	16,24	26,42	26,42	26,42	26,42	23,74	23,74	23,74	23,74
22	13,07	13,07	13,07	13,07	15,95	15,95	15,95	15,95	26,01	26,01	26,01	26,01	23,49	23,49	23,49	23,49
23	13,47	13,47	13,47	13,47	15,96	15,96	15,96	15,96	25,82	25,82	25,82	25,82	23,45	23,45	23,45	23,45
24	13,87	13,87	13,87	13,87	15,97	15,97	15,97	15,97	25,64	25,64	25,64	25,64	23,41	23,41	23,41	23,41
4. Generation of ambient temperature and solar radiation data with the use of the PVGIS tool [1]. In order to define the typical day for which the simulations are to be performed, the data of each month was statistically processed to determine the day whose mean statistical deviation was the smallest in relation to the mean values of the month, based on the following equation (standard deviation) [4]:

$S=\sqrt{\frac{\sum_{i=1}^{N}{\left(x_{i}-\bar{x}\right)}^{2}}{N-1}}$

5. Definition of boundary conditions,of which the exterior boundary conditions were calculated using the sol-air temperature equation [5]:
  $T_{sol-air}=T_{0}+\;\frac{\left(aI-\Delta Q_{ir}\right)}{h_{0}}$
  The temperature values used as exterior boundary conditions are presented in Table 3 while the boundary conditions imposed on the interior side of the investigated building element was kept constant at 22 °C.The boundary conditions within the building element across the different layers were defined as open boundary. The air flow within the novel controlled-temperature double skin façade was considered to have a constant velocity of 0.05 m/s and a constant temperature of 22 °C.
6. Conduction of numerical simulation study for the four seasons (winter, spring, summer, autumn) and for four orientations of the building (azimuth 0°, 90°, 180° and 270°), which provided the hourly heat flux data

## Ethics Statement

No ethical issues are associated with this work.

## CRediT Author Statement

**Angeliki Kylili:** Writing original draft, Writing review & editing. **Loucas Georgiou:** Formal analysis, Investigation, Writing review & editing; **Paris A. Fokaides:** Conceptualization, Methodology, Validation, Resources, Visualization, Supervision, Project administration.

## Declaration of competing interest

The authors declare that they have no known competing financial interests or personal relationships which have, or could be perceived to have, influenced the work reported in this article.

## Appendix Supplementary materials

Image, application 1

Image, application 2
