# Supplementary material for: Numerical data on heat flux of a novel controlled-temperature double skin façade
Source: Data Brief. 2021 Apr 20;36:107034. doi: 10.1016/j.dib.2021.107034 (PMC8257991; doi:10.1016/j.dib.2021.107034)
Supplement: Supplementary file 1 [file mmc1.docx]

| **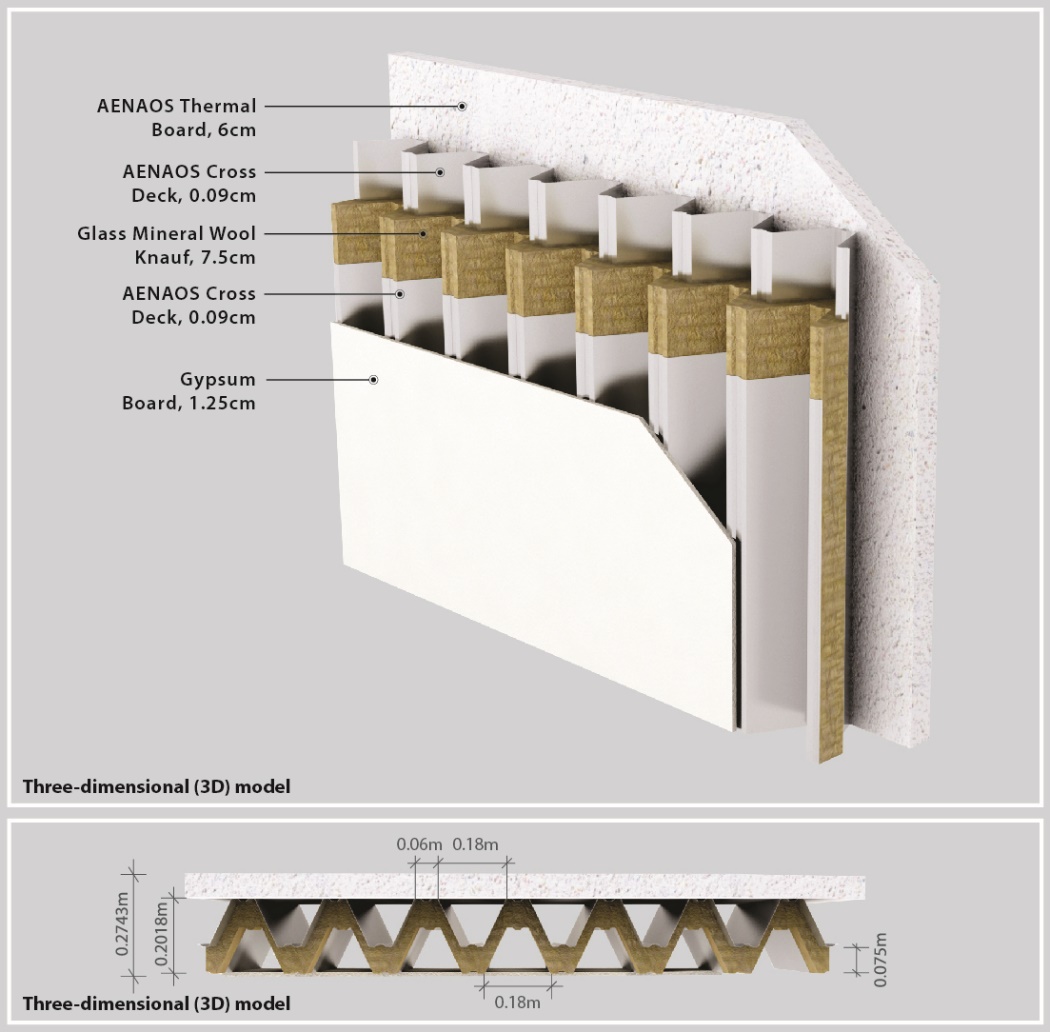** | **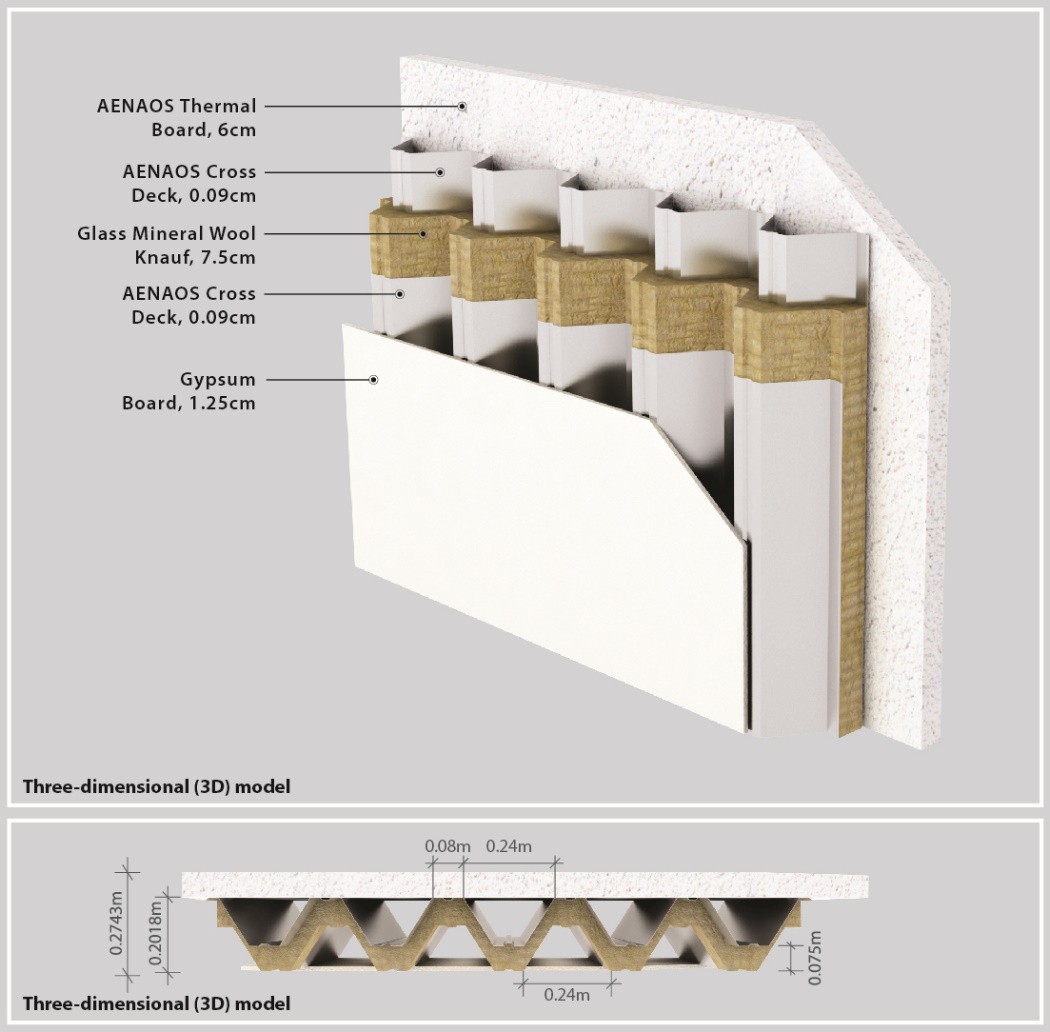** |
| --- | --- |
| **Reference Figure 1.** Investigated geometry 1 of novel double-skin façade (DSF) controlled-temperature building element | **Reference Figure 2.** Investigated geometry 2 of novel double-skin façade (DSF) controlled-temperature building element |

| **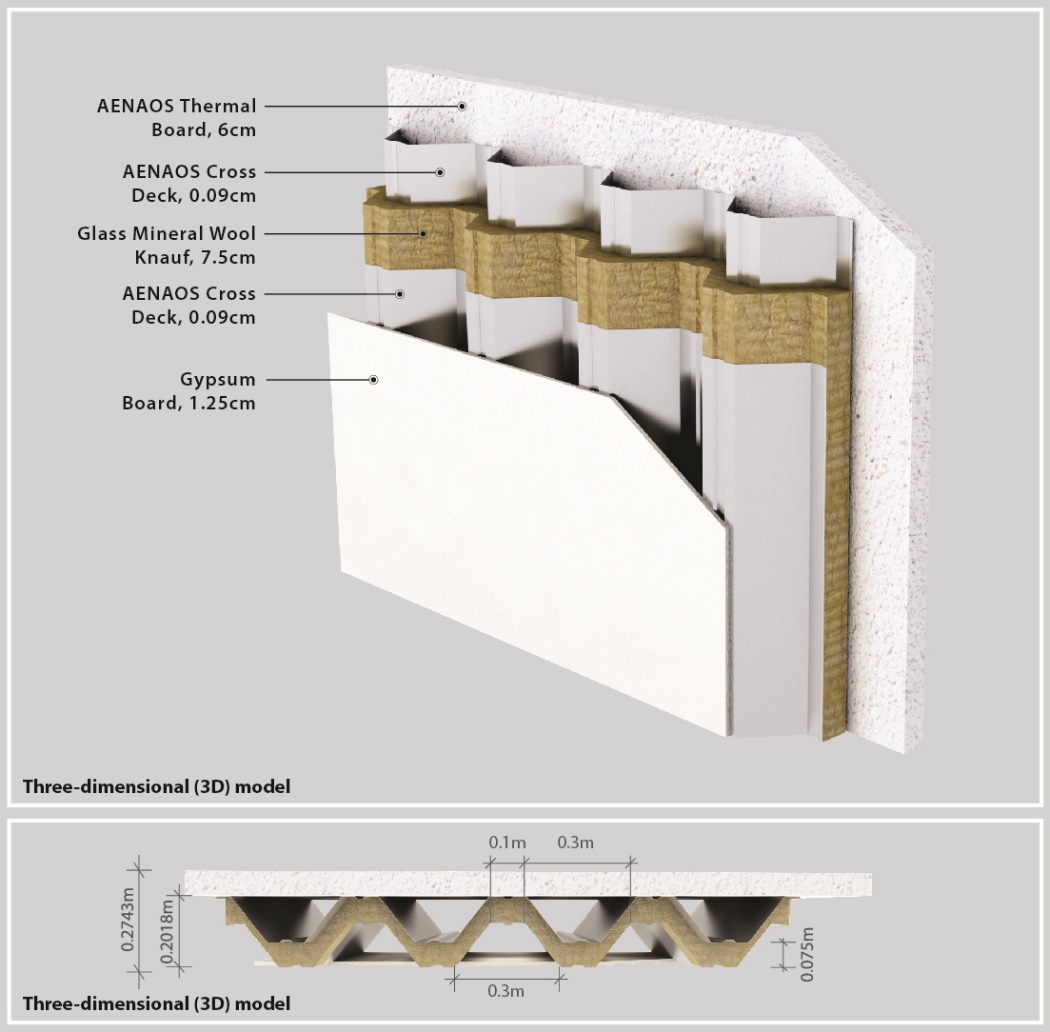** | **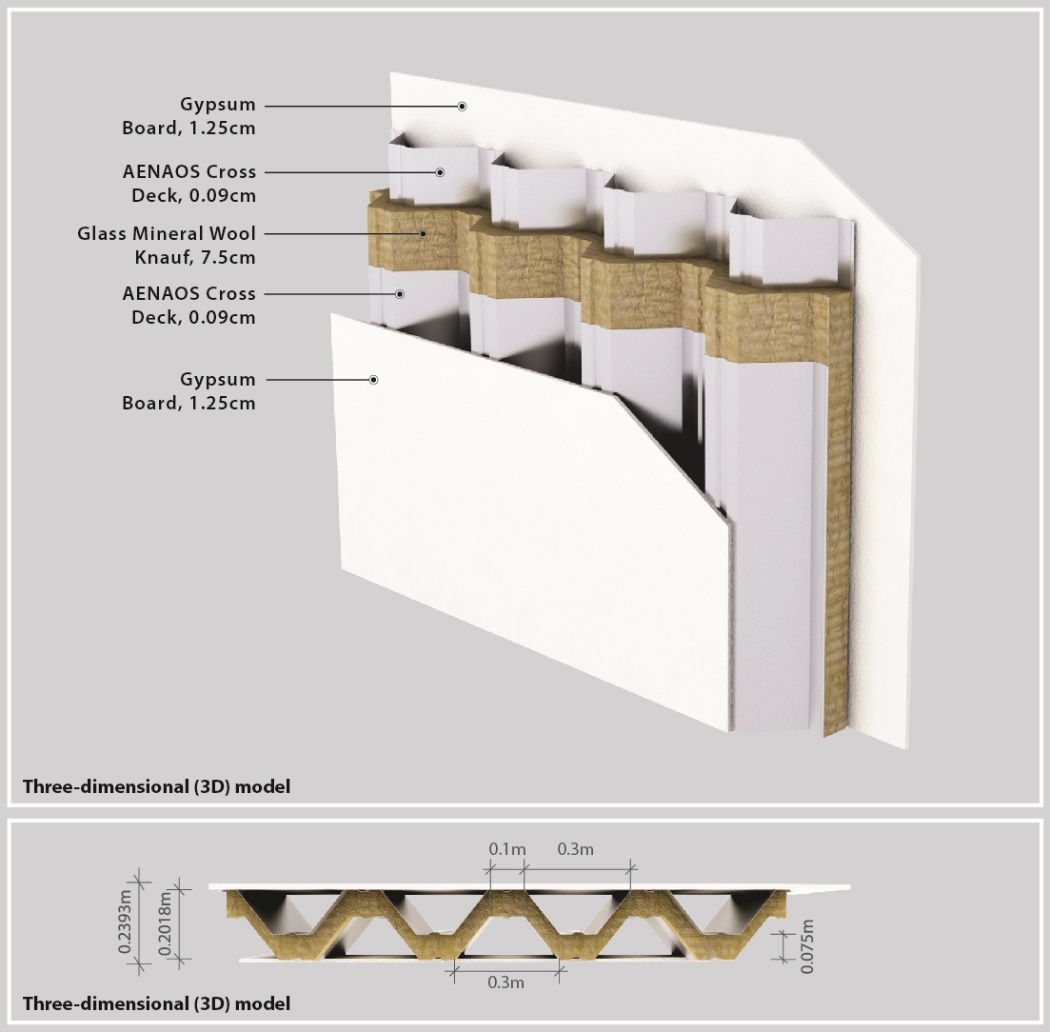** |
| --- | --- |
| **Reference Figure 3.** Investigated geometry 3 of novel double-skin façade (DSF) controlled-temperature building element | **Reference Figure 4.** Investigated geometry 4 of novel double-skin façade (DSF) controlled-temperature building element |

| **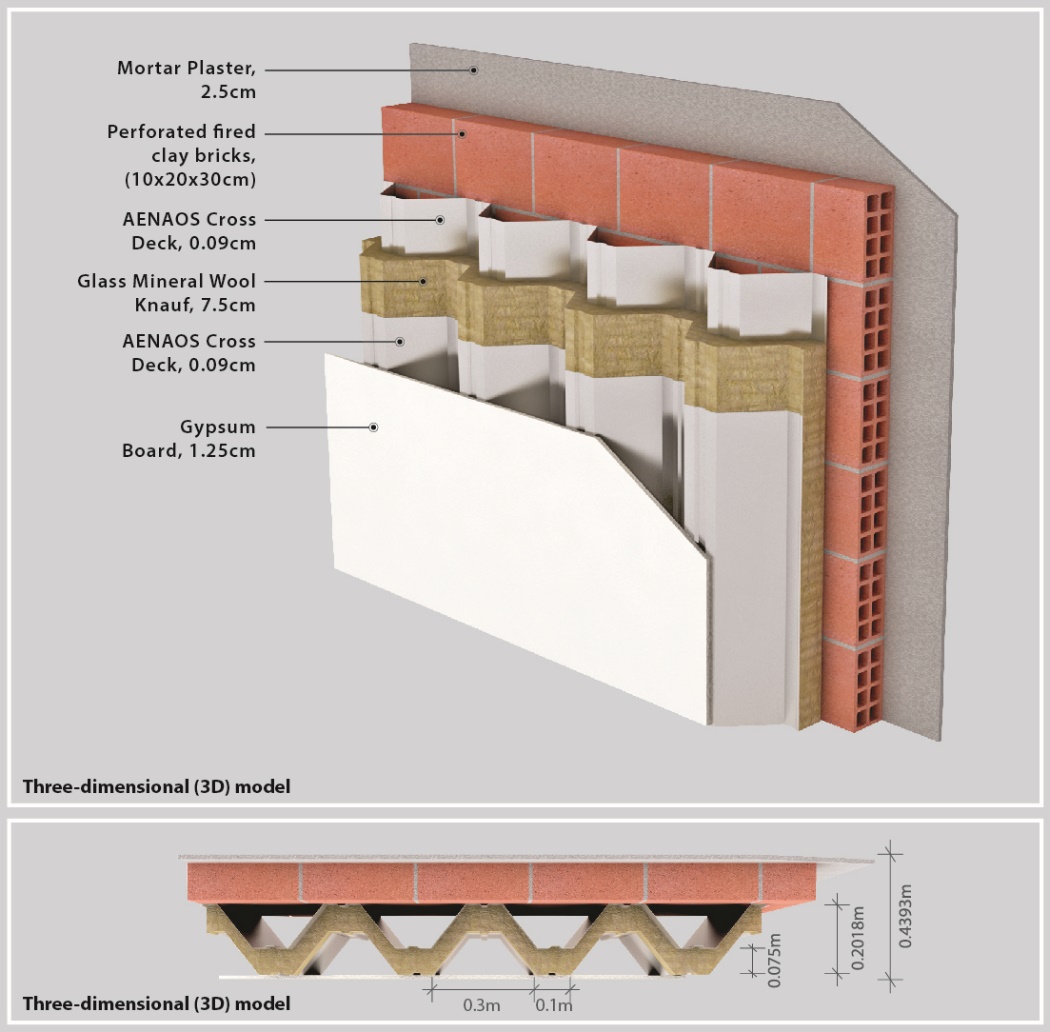** | **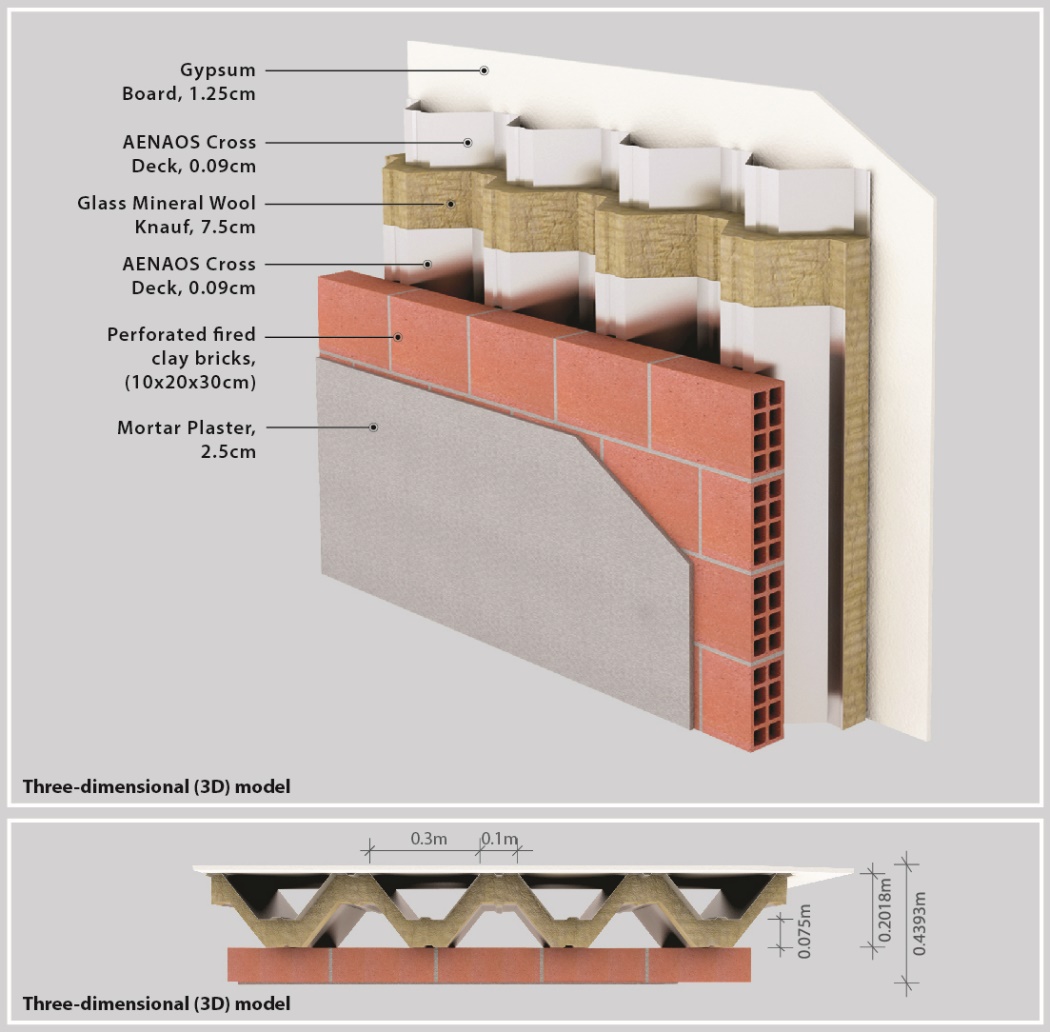** |
| --- | --- |
| **Reference Figure 5.** Investigated geometry 5 of novel double-skin façade (DSF) controlled-temperature building element | **Reference Figure 6.** Investigated geometry 6 of novel double-skin façade (DSF) controlled-temperature building element |


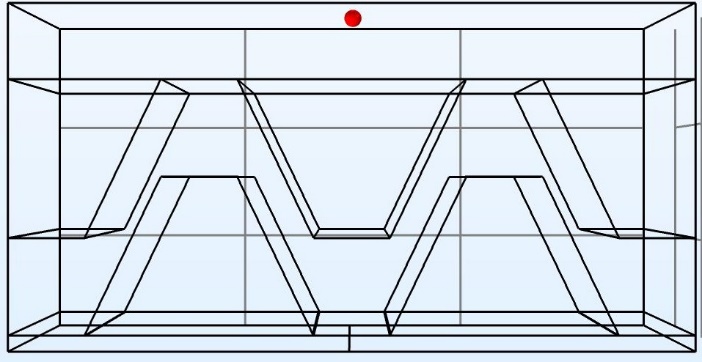


**Point 1**


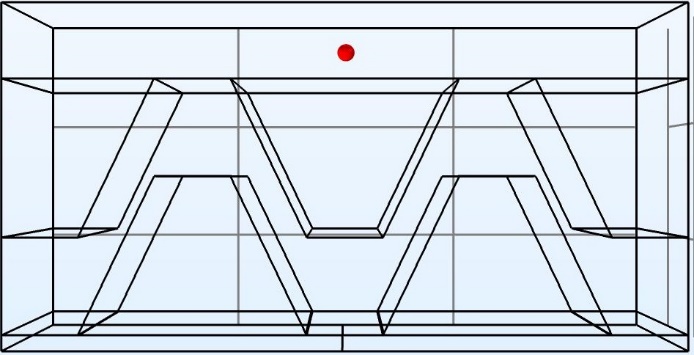


**Point 2**


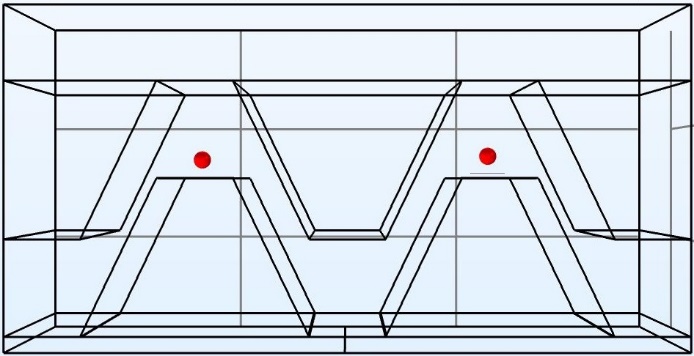


**Point 3**

**Reference Figure 7.** Locations of points 1,2,3 in investigated geometry 1 of novel double-skin façade (DSF)


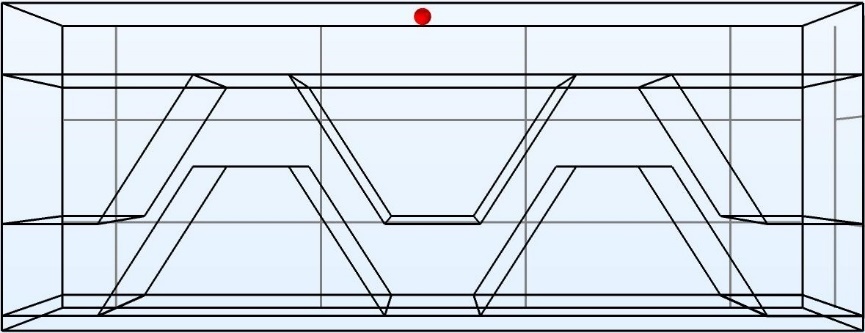


**Point 1**


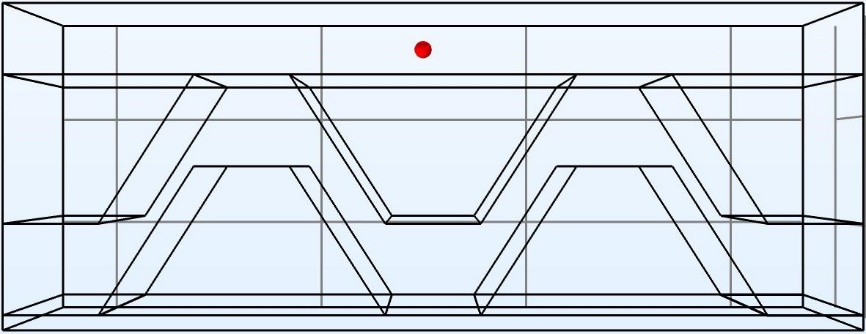


**Point 2**


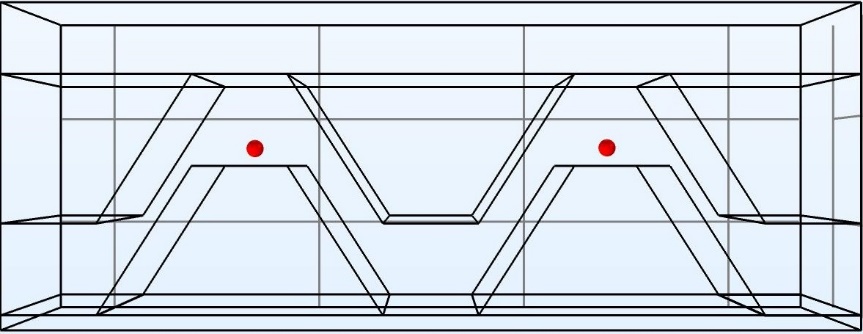


**Point 3**

**Reference Figure 8.** Locations of points 1,2,3 in investigated geometry 2 of novel double-skin façade (DSF)


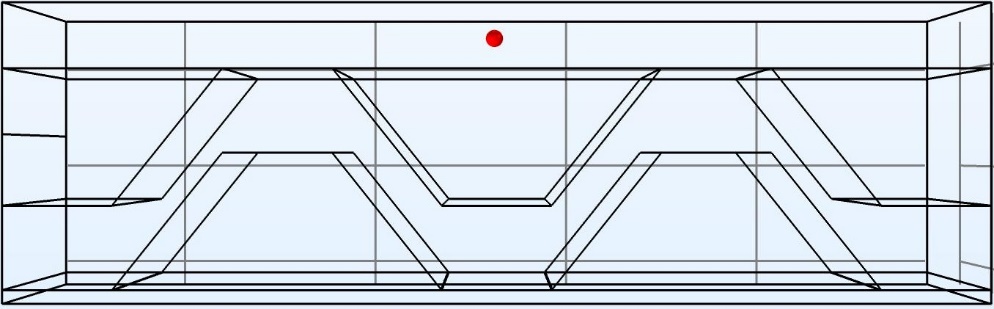


**Point 1**


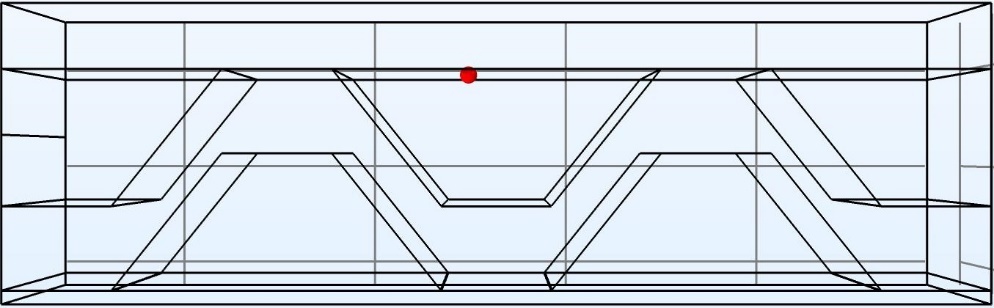


**Point 2**


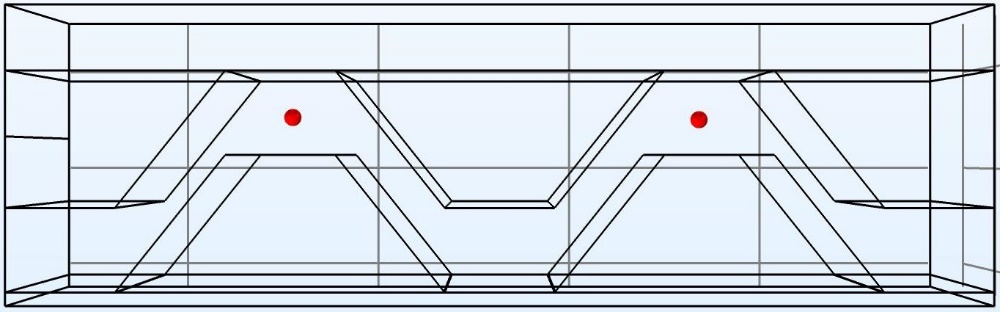


**Point 3**

**Reference Figure 9.** Locations of points 1,2,3 in investigated geometry 3 of novel double-skin façade (DSF)


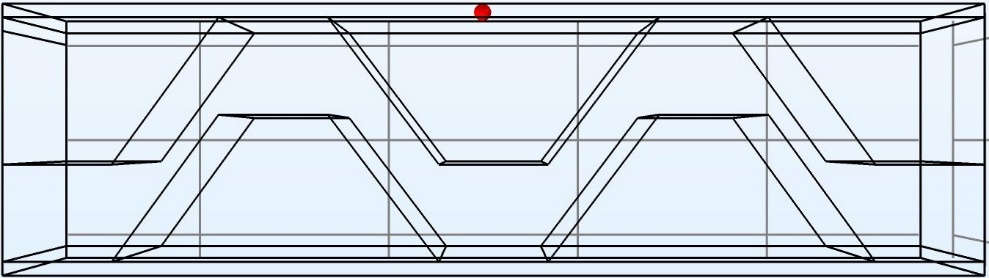


**Point 1**


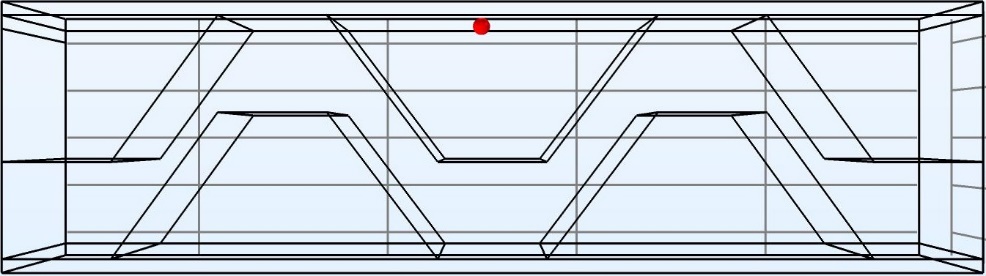


**Point 2**


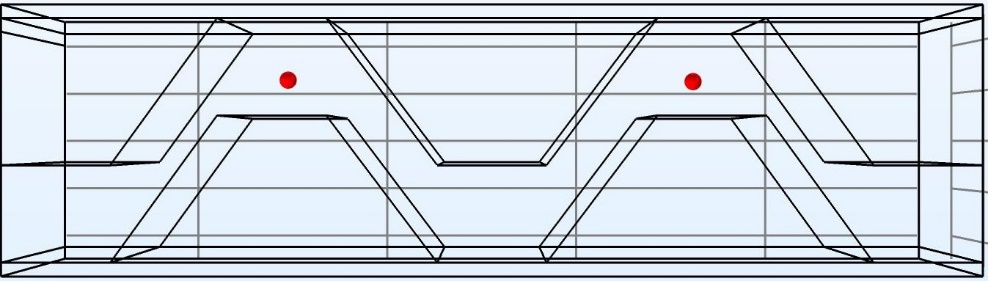


**Point 3**

**Reference Figure 10.** Locations of points 1,2,3 in investigated geometry 4 of novel double-skin façade (DSF)


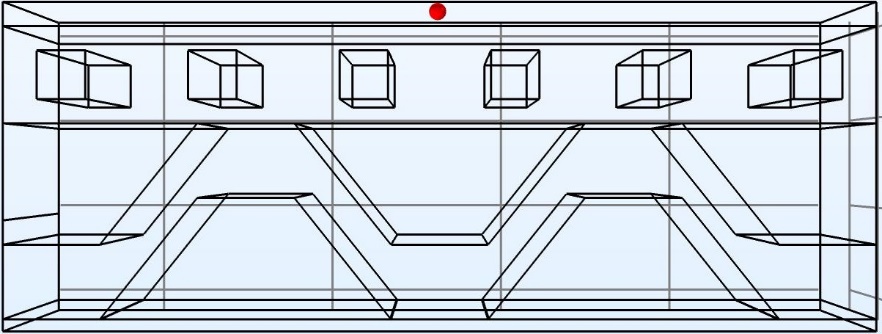


**Point 1**


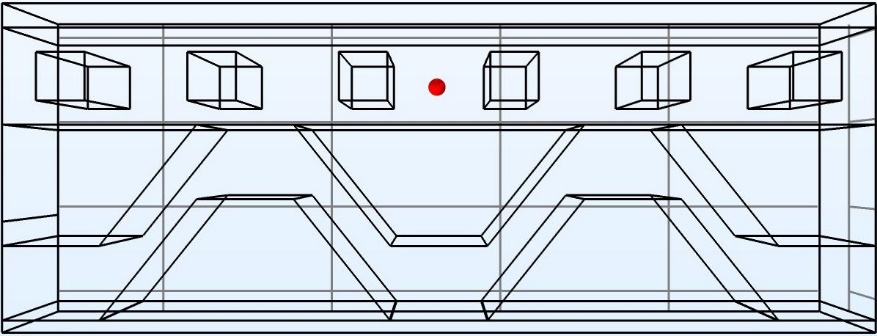


**Point 2**


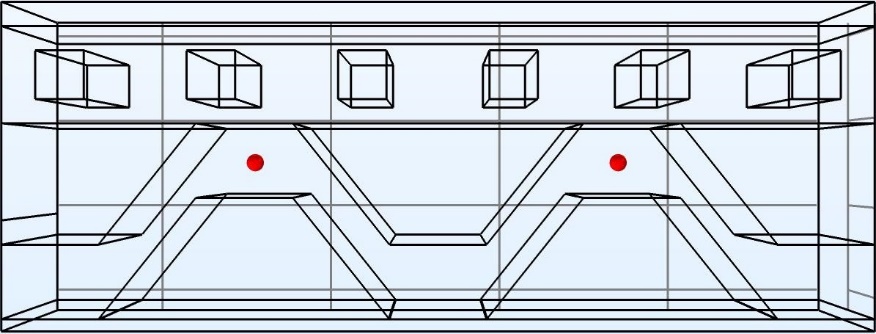


**Point 3**

**Reference Figure 11.** Locations of points 1,2,3 in investigated geometry 5 of novel double-skin façade (DSF)


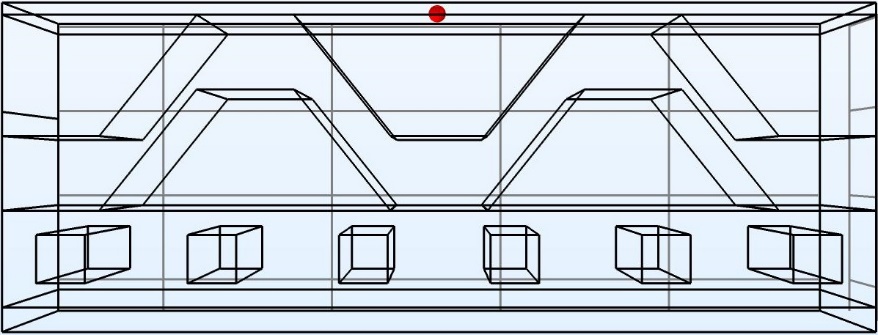


**Point 1**


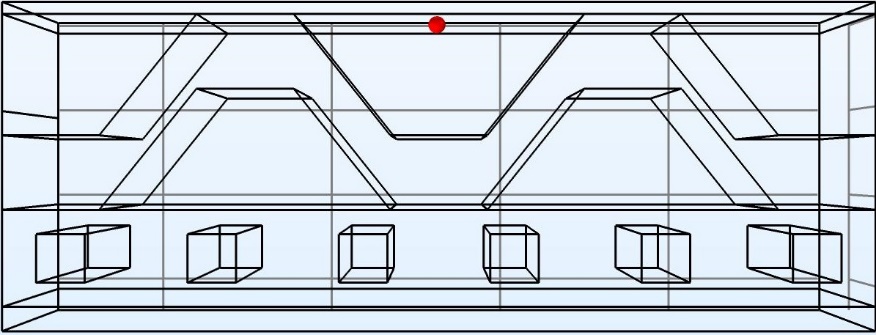


**Point 2**


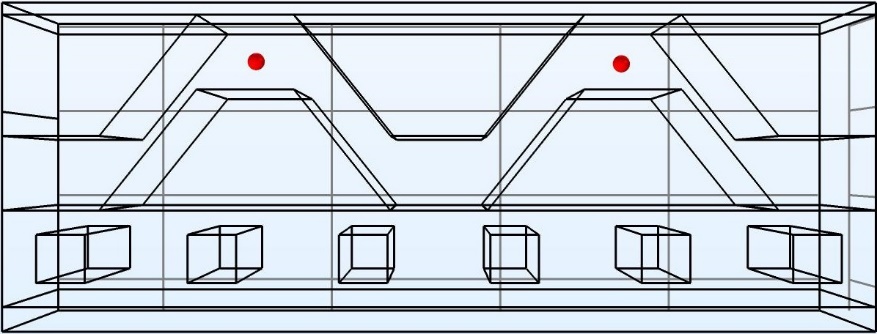


**Point 3**

**Reference Figure 12.** Locations of points 1,2,3 in investigated geometry 6 of novel double-skin façade (DSF)

**Table A1.** Hourly heat flux (W/ m^2^) for geometry 1 of novel double-skin façade (DSF) controlled-temperature building element

| **Season/ Heat flux [W/m^2^]** | **Winter** | | | | **Spring** | | | | **Summer** | | | | **Autumn** | | | |
| --- | --- | --- | --- | --- | --- | --- | --- | --- | --- | --- | --- | --- | --- | --- | --- | --- |
| **Time [hr]** | **Azimuth [°]** | | | | | | | | | | | | | | | |
|  | **0** | **90** | **180** | **270** | **0** | **90** | **180** | **270** | **0** | **90** | **180** | **270** | **0** | **90** | **180** | **270** |
| **1** | 1,45 | 1,45 | 1,47 | 1,46 | 0,90 | 0,93 | 0,90 | 0,90 | -0,48 | -0,45 | -0,49 | -0,51 | -0,25 | -0,26 | -0,27 | -0,27 |
| **2** | 1,62 | 1,79 | 1,79 | 1,78 | 0,98 | 0,95 | 0,97 | 0,97 | -0,46 | -0,46 | -0,48 | -0,49 | -0,26 | -0,27 | -0,29 | -0,29 |
| **3** | 1,54 | 1,62 | 1,62 | 1,61 | 0,98 | 0,96 | 0,98 | 0,97 | -0,37 | -0,38 | -0,35 | -0,35 | -0,22 | -0,23 | -0,21 | -0,21 |
| **4** | 1,37 | 1,43 | 1,43 | 1,43 | 0,96 | 0,96 | 0,95 | 0,96 | -0,34 | -0,34 | -0,42 | -0,55 | -0,19 | -0,19 | -0,15 | -0,15 |
| **5** | 1,30 | 1,34 | 1,34 | 1,34 | 0,57 | 0,57 | 0,18 | -1,49 | -0,86 | -0,86 | -2,06 | -4,35 | -0,30 | -0,25 | -0,24 | -0,46 |
| **6** | 1,21 | 1,28 | 1,28 | 1,30 | -0,33 | 0,09 | -0,28 | -4,46 | -1,35 | -1,35 | -3,06 | -8,36 | -1,81 | -0,56 | -0,56 | -3,58 |
| **7** | -0,84 | 1,02 | 1,02 | 1,10 | -1,87 | -0,24 | -0,28 | -5,27 | -1,89 | -1,58 | -2,06 | -7,99 | -3,44 | -0,84 | -0,84 | -5,05 |
| **8** | -2,75 | 0,65 | 0,65 | -1,56 | -3,14 | -0,37 | -0,27 | -4,18 | -2,97 | -1,56 | -1,11 | -4,35 | -4,66 | -1,05 | -1,05 | -4,41 |
| **9** | -3,51 | 0,49 | 0,49 | -2,85 | -3,45 | -0,48 | -0,41 | -2,09 | -3,82 | -1,46 | -0,97 | -1,35 | -5,18 | -1,29 | -1,29 | -2,71 |
| **10** | -3,01 | 0,60 | 0,60 | -1,82 | -3,02 | -0,42 | -0,40 | 0,05 | -3,83 | -1,41 | -1,18 | -0,12 | -5,05 | -1,39 | -1,39 | -0,84 |
| **11** | -1,77 | 0,23 | 0,80 | 0,05 | -2,17 | -1,55 | -0,42 | 1,81 | -3,19 | -2,51 | -1,33 | 0,82 | -3,93 | -2,64 | -1,36 | 0,25 |
| **12** | -0,71 | -0,93 | 0,87 | 1,84 | -1,33 | -2,99 | -0,26 | 1,42 | -2,39 | -4,42 | -1,36 | 0,27 | -2,73 | -3,94 | -1,01 | 0,18 |
| **13** | -0,03 | -1,74 | 0,82 | 1,94 | -0,47 | -3,75 | 0,02 | 0,16 | -1,57 | -5,79 | -1,36 | -1,10 | -1,98 | -4,41 | -0,78 | -0,34 |
| **14** | 0,52 | -1,54 | 0,97 | 1,47 | 0,24 | -3,92 | 0,18 | -0,27 | -0,77 | -5,97 | -1,25 | -1,61 | -0,82 | -3,34 | -0,63 | -0,51 |
| **15** | 1,91 | 0,10 | 1,36 | 1,26 | 0,95 | -3,60 | 0,34 | 0,30 | 0,04 | -5,56 | -1,68 | -1,06 | 0,44 | -1,08 | -0,37 | -0,34 |
| **16** | 3,29 | 2,49 | 1,61 | 1,40 | 1,60 | -1,68 | 0,26 | 0,88 | -0,10 | -4,18 | -2,21 | -0,56 | 1,42 | 2,10 | -0,03 | -0,04 |
| **17** | 2,76 | 2,92 | 1,46 | 1,53 | 1,72 | 2,81 | 0,86 | 1,21 | -0,59 | -1,03 | -1,87 | -0,45 | 0,91 | 2,27 | -0,01 | -0,01 |
| **18** | 1,37 | 1,57 | 1,22 | 1,46 | 1,28 | 4,21 | 1,33 | 1,15 | -0,59 | 3,12 | -0,24 | -0,42 | -0,14 | 0,20 | -0,19 | -0,09 |
| **19** | 0,86 | 0,67 | 1,18 | 1,36 | 0,94 | 2,64 | 1,26 | 1,00 | -0,38 | 2,95 | 0,23 | -0,44 | -0,45 | -0,94 | -0,25 | -0,13 |
| **20** | 1,24 | 0,93 | 1,25 | 1,28 | 0,92 | 1,28 | 1,06 | 0,91 | -0,34 | -0,04 | -0,02 | -0,48 | -0,12 | -0,54 | -0,16 | -0,13 |
| **21** | 1,53 | 1,20 | 1,28 | 1,24 | 1,02 | 0,86 | 0,96 | 0,92 | -0,42 | -1,50 | -0,29 | -0,47 | 0,12 | -0,15 | -0,09 | -0,12 |
| **22** | 1,40 | 1,27 | 1,25 | 1,24 | 1,05 | 0,90 | 0,96 | 0,97 | -0,42 | -0,90 | -0,37 | -0,40 | 0,03 | -0,04 | -0,07 | -0,09 |
| **23** | 1,09 | 1,15 | 1,11 | 1,14 | 0,95 | 0,93 | 0,93 | 0,94 | -0,40 | -0,39 | -0,39 | -0,37 | -0,15 | -0,09 | -0,13 | -0,12 |
| **24** | 0,98 | 0,97 | 0,97 | 0,99 | 0,84 | 0,90 | 0,87 | 0,86 | -0,39 | -0,30 | -0,39 | -0,40 | -0,17 | -0,15 | -0,18 | -0,16 |

**Table A2.** Hourly heat flux (W/ m^2^) for geometry 2 of novel double-skin façade (DSF) controlled-temperature building element

| **Season/ Heat flux [W/m^2^]** | **Winter** | | | | **Spring** | | | | **Summer** | | | | **Autumn** | | | |
| --- | --- | --- | --- | --- | --- | --- | --- | --- | --- | --- | --- | --- | --- | --- | --- | --- |
| **Time [hr]** | **Azimuth [°]** | | | | | | | | | | | | | | | |
|  | **0** | **90** | **180** | **270** | **0** | **90** | **180** | **270** | **0** | **90** | **180** | **270** | **0** | **90** | **180** | **270** |
| **1** | 1,47 | 1,47 | 1,48 | 1,48 | 0,90 | 0,93 | 0,90 | 0,90 | -0,48 | -0,45 | -0,49 | -0,51 | -0,25 | -0,26 | -0,27 | -0,27 |
| **2** | 1,63 | 1,80 | 1,80 | 1,80 | 0,98 | 0,95 | 0,97 | 0,97 | -0,45 | -0,46 | -0,48 | -0,49 | -0,26 | -0,27 | -0,28 | -0,29 |
| **3** | 1,54 | 1,62 | 1,62 | 1,62 | 0,98 | 0,96 | 0,98 | 0,97 | -0,37 | -0,37 | -0,34 | -0,34 | -0,22 | -0,22 | -0,21 | -0,21 |
| **4** | 1,37 | 1,43 | 1,43 | 1,43 | 0,96 | 0,96 | 0,95 | 0,96 | -0,33 | -0,33 | -0,42 | -0,55 | -0,18 | -0,18 | -0,15 | -0,14 |
| **5** | 1,30 | 1,34 | 1,34 | 1,34 | 0,56 | 0,56 | 0,17 | -1,51 | -0,87 | -0,87 | -2,08 | -4,40 | -0,30 | -0,25 | -0,24 | -0,46 |
| **6** | 1,21 | 1,29 | 1,29 | 1,31 | -0,34 | 0,09 | -0,29 | -4,49 | -1,35 | -1,35 | -3,07 | -8,39 | -1,82 | -0,57 | -0,56 | -3,60 |
| **7** | -0,87 | 1,02 | 1,03 | 1,10 | -1,89 | -0,25 | -0,27 | -5,25 | -1,88 | -1,57 | -2,03 | -7,86 | -3,44 | -0,84 | -0,84 | -5,04 |
| **8** | -2,77 | 0,65 | 0,73 | -1,61 | -3,15 | -0,37 | -0,27 | -4,11 | -2,96 | -1,55 | -1,07 | -4,10 | -4,64 | -1,05 | -1,05 | -4,35 |
| **9** | -3,49 | 0,49 | 0,56 | -2,87 | -3,43 | -0,47 | -0,40 | -2,01 | -3,80 | -1,44 | -0,94 | -1,15 | -5,12 | -1,28 | -1,28 | -2,62 |
| **10** | -2,96 | 0,61 | 0,57 | -1,79 | -2,97 | -0,41 | -0,39 | 0,13 | -3,77 | -1,39 | -1,16 | -0,02 | -4,96 | -1,38 | -1,38 | -0,76 |
| **11** | -1,71 | 0,23 | 0,69 | 0,10 | -2,11 | -1,55 | -0,41 | 1,85 | -3,10 | -2,50 | -1,31 | 0,87 | -3,81 | -2,64 | -1,34 | 0,31 |
| **12** | -0,67 | -0,95 | 0,78 | 1,88 | -1,28 | -2,99 | -0,25 | 1,40 | -2,30 | -4,40 | -1,34 | 0,29 | -2,61 | -3,93 | -0,99 | 0,20 |
| **13** | 0,00 | -1,75 | 0,81 | 1,94 | -0,43 | -3,73 | 0,02 | 0,12 | -1,49 | -5,74 | -1,34 | -1,11 | -1,88 | -4,36 | -0,76 | -0,34 |
| **14** | 0,54 | -1,53 | 1,00 | 1,45 | 0,27 | -3,88 | 0,19 | -0,28 | -0,72 | -5,85 | -1,23 | -1,61 | -0,74 | -3,25 | -0,61 | -0,51 |
| **15** | 1,93 | 0,14 | 1,31 | 1,25 | 0,97 | -3,53 | 0,34 | 0,32 | 0,08 | -5,41 | -1,66 | -1,03 | 0,50 | -0,97 | -0,35 | -0,33 |
| **16** | 3,30 | 2,52 | 1,55 | 1,39 | 1,60 | -1,60 | 0,25 | 0,89 | -0,09 | -4,00 | -2,19 | -0,53 | 1,44 | 2,19 | -0,02 | -0,04 |
| **17** | 2,72 | 2,91 | 1,44 | 1,53 | 1,71 | 2,89 | 0,87 | 1,19 | -0,60 | -0,84 | -1,84 | -0,43 | 0,89 | 2,27 | 0,00 | -0,01 |
| **18** | 1,31 | 1,52 | 1,23 | 1,46 | 1,26 | 4,21 | 1,34 | 1,14 | -0,59 | 3,26 | -0,20 | -0,41 | -0,17 | 0,13 | -0,19 | -0,09 |
| **19** | 0,85 | 0,65 | 1,18 | 1,35 | 0,92 | 2,58 | 1,25 | 0,99 | -0,37 | 2,95 | 0,26 | -0,43 | -0,46 | -0,97 | -0,25 | -0,13 |
| **20** | 1,26 | 0,93 | 1,25 | 1,28 | 0,91 | 1,23 | 1,05 | 0,91 | -0,33 | -0,14 | -0,01 | -0,48 | -0,10 | -0,54 | -0,16 | -0,13 |
| **21** | 1,54 | 1,21 | 1,28 | 1,25 | 1,02 | 0,83 | 0,95 | 0,92 | -0,42 | -1,55 | -0,29 | -0,47 | 0,12 | -0,15 | -0,08 | -0,12 |
| **22** | 1,39 | 1,27 | 1,25 | 1,24 | 1,04 | 0,89 | 0,96 | 0,97 | -0,42 | -0,89 | -0,37 | -0,39 | 0,02 | -0,04 | -0,08 | -0,09 |
| **23** | 1,08 | 1,15 | 1,12 | 1,14 | 0,94 | 0,93 | 0,93 | 0,94 | -0,39 | -0,38 | -0,39 | -0,37 | -0,16 | -0,09 | -0,13 | -0,12 |
| **24** | 0,98 | 0,97 | 0,97 | 0,99 | 0,84 | 0,90 | 0,86 | 0,86 | -0,39 | -0,30 | -0,39 | -0,40 | -0,17 | -0,15 | -0,18 | -0,16 |

**Table A3.** Hourly heat flux (W/ m^2^) for geometry 3 of novel double-skin façade (DSF) controlled-temperature building element

| **Season/ Heat flux [W/m^2^]** | **Winter** | | | | **Spring** | | | | **Summer** | | | | **Autumn** | | | |
| --- | --- | --- | --- | --- | --- | --- | --- | --- | --- | --- | --- | --- | --- | --- | --- | --- |
| **Time [hr]** | **Azimuth [°]** | | | | | | | | | | | | | | | |
|  | **0** | **90** | **180** | **270** | **0** | **90** | **180** | **270** | **0** | **90** | **180** | **270** | **0** | **90** | **180** | **270** |
| **1** | 1,51 | 1,43 | 1,48 | 1,48 | 0,90 | 0,73 | 0,88 | 0,90 | -0,50 | -0,59 | -0,47 | -0,51 | -0,25 | -0,28 | -0,27 | -0,27 |
| **2** | 1,82 | 1,62 | 1,80 | 1,80 | 0,98 | 0,90 | 0,94 | 0,97 | -0,49 | -0,61 | -0,48 | -0,49 | -0,25 | -0,25 | -0,28 | -0,29 |
| **3** | 1,62 | 1,54 | 1,56 | 1,57 | 0,98 | 0,97 | 0,96 | 0,97 | -0,34 | -0,29 | -0,35 | -0,34 | -0,22 | -0,21 | -0,21 | -0,21 |
| **4** | 1,43 | 1,43 | 1,27 | 1,27 | 0,94 | 0,98 | 0,96 | 0,96 | -0,28 | -0,20 | -0,42 | -0,56 | -0,18 | -0,18 | -0,15 | -0,14 |
| **5** | 1,33 | 1,35 | 1,24 | 1,24 | 0,51 | 0,56 | 0,18 | -1,52 | -0,85 | -0,92 | -2,08 | -4,42 | -0,30 | -0,25 | -0,24 | -0,49 |
| **6** | 1,20 | 1,29 | 1,31 | 1,33 | -0,54 | 0,09 | -0,29 | -4,49 | -1,35 | -1,61 | -3,06 | -8,39 | -1,82 | -0,57 | -0,63 | -3,97 |
| **7** | -0,72 | 1,01 | 1,06 | 1,14 | -2,04 | -0,28 | -0,22 | -5,33 | -1,88 | -1,70 | -1,80 | -7,81 | -3,44 | -0,83 | -0,96 | -6,05 |
| **8** | -2,12 | 0,64 | 0,64 | -1,63 | -3,17 | -0,35 | -0,11 | -3,44 | -2,96 | -1,41 | -0,36 | -4,05 | -4,63 | -1,04 | -1,10 | -4,37 |
| **9** | -3,16 | 0,49 | 0,47 | -2,88 | -3,40 | -0,38 | -0,32 | -0,72 | -3,79 | -1,23 | -0,54 | -1,14 | -5,02 | -1,28 | -1,25 | -1,35 |
| **10** | -3,10 | 0,61 | 0,61 | -1,40 | -2,96 | -0,33 | -0,42 | 1,12 | -3,76 | -1,31 | -1,45 | -0,04 | -4,65 | -1,37 | -1,35 | 0,43 |
| **11** | -1,89 | 0,23 | 0,82 | 1,08 | -2,11 | -1,59 | -0,40 | 1,96 | -3,09 | -2,63 | -1,70 | 0,88 | -3,33 | -2,64 | -1,34 | 0,54 |
| **12** | -0,64 | -0,96 | 0,88 | 2,72 | -1,27 | -3,43 | -0,19 | 1,15 | -2,29 | -4,92 | -1,36 | 0,31 | -2,03 | -3,93 | -0,99 | -0,29 |
| **13** | 0,09 | -1,74 | 0,81 | 2,04 | -0,41 | -4,12 | 0,13 | 0,08 | -1,48 | -6,07 | -1,16 | -1,12 | -1,58 | -4,35 | -0,76 | -0,97 |
| **14** | 0,56 | -1,41 | 0,98 | 0,77 | 0,27 | -3,69 | 0,23 | -0,14 | -0,71 | -5,84 | -1,16 | -1,62 | -0,79 | -3,22 | -0,61 | -0,81 |
| **15** | 1,90 | 0,61 | 1,37 | 0,67 | 0,97 | -3,03 | 0,33 | 0,39 | 0,09 | -5,32 | -1,72 | -1,03 | 0,55 | -0,95 | -0,35 | -0,22 |
| **16** | 3,29 | 3,36 | 1,61 | 1,43 | 1,60 | -1,09 | 0,25 | 0,84 | -0,09 | -4,00 | -2,40 | -0,53 | 1,57 | 2,20 | -0,02 | 0,19 |
| **17** | 2,71 | 3,32 | 1,45 | 1,83 | 1,70 | 3,79 | 0,88 | 1,16 | -0,61 | -0,86 | -1,93 | -0,44 | 0,92 | 2,25 | -0,01 | 0,06 |
| **18** | 1,30 | 1,44 | 1,21 | 1,52 | 1,25 | 4,80 | 1,35 | 1,15 | -0,59 | 3,28 | 0,21 | -0,41 | -0,22 | 0,11 | -0,19 | -0,22 |
| **19** | 0,85 | 0,56 | 1,18 | 1,17 | 0,91 | 1,87 | 1,21 | 1,02 | -0,36 | 2,94 | 0,91 | -0,43 | -0,47 | -0,96 | -0,25 | -0,20 |
| **20** | 1,27 | 1,06 | 1,26 | 1,19 | 0,91 | -0,18 | 0,87 | 0,90 | -0,33 | -0,18 | -0,12 | -0,47 | -0,08 | -0,39 | -0,16 | -0,15 |
| **21** | 1,54 | 1,57 | 1,28 | 1,24 | 1,02 | 0,37 | 0,80 | 0,90 | -0,42 | -1,55 | -0,90 | -0,47 | 0,13 | 0,26 | -0,08 | -0,12 |
| **22** | 1,38 | 1,46 | 1,24 | 1,24 | 1,04 | 1,45 | 0,96 | 0,96 | -0,42 | -0,63 | -0,65 | -0,39 | 0,01 | 0,19 | -0,08 | -0,09 |
| **23** | 1,08 | 1,07 | 1,11 | 1,14 | 0,94 | 1,42 | 0,98 | 0,94 | -0,38 | 0,22 | -0,42 | -0,37 | -0,16 | -0,17 | -0,13 | -0,12 |
| **24** | 0,94 | 0,90 | 0,97 | 0,98 | 0,84 | 0,83 | 0,87 | 0,86 | -0,38 | -0,02 | -0,37 | -0,40 | -0,17 | -0,26 | -0,17 | -0,16 |

**Table A4**. Hourly heat flux (W/ m^2^) for geometry 4 of novel double-skin façade (DSF) controlled-temperature building element

| **Season/ Heat flux [W/m^2^]** | **Winter** | | | | **Spring** | | | | **Summer** | | | | **Autumn** | | | |
| --- | --- | --- | --- | --- | --- | --- | --- | --- | --- | --- | --- | --- | --- | --- | --- | --- |
| **Time [hr]** | **Azimuth [°]** | | | | | | | | | | | | | | | |
|  | **0** | **90** | **180** | **270** | **0** | **90** | **180** | **270** | **0** | **90** | **180** | **270** | **0** | **90** | **180** | **270** |
| **1** | 1,34 | 1,34 | 1,34 | 1,34 | 0,85 | 0,86 | 0,85 | 0,85 | -0,52 | -0,51 | -0,53 | -0,52 | -0,24 | -0,24 | -0,24 | -0,24 |
| **2** | 1,41 | 1,41 | 1,41 | 1,41 | 0,88 | 0,88 | 0,88 | 0,88 | -0,49 | -0,48 | -0,50 | -0,49 | -0,24 | -0,24 | -0,24 | -0,24 |
| **3** | 1,36 | 1,36 | 1,36 | 1,36 | 0,89 | 0,89 | 0,89 | 0,89 | -0,43 | -0,42 | -0,43 | -0,43 | -0,21 | -0,21 | -0,21 | -0,21 |
| **4** | 1,31 | 1,31 | 1,31 | 1,31 | 0,89 | 0,89 | 0,89 | 0,89 | -0,40 | -0,40 | -0,47 | -0,56 | -0,19 | -0,19 | -0,19 | -0,19 |
| **5** | 1,32 | 1,32 | 1,32 | 1,32 | 0,65 | 0,65 | 0,44 | -1,00 | -0,70 | -0,70 | -1,46 | -3,18 | -0,27 | -0,24 | -0,24 | -0,37 |
| **6** | 1,26 | 1,31 | 1,31 | 1,32 | 0,07 | 0,35 | 0,25 | -2,29 | -0,96 | -0,96 | -1,52 | -4,17 | -1,42 | -0,41 | -0,41 | -2,47 |
| **7** | -0,05 | 1,16 | 1,16 | 1,20 | -0,73 | 0,21 | 0,24 | -2,36 | -1,27 | -1,09 | -1,12 | -4,03 | -2,07 | -0,56 | -0,56 | -2,56 |
| **8** | -0,67 | 0,98 | 0,98 | -0,48 | -1,34 | 0,14 | 0,17 | -2,15 | -1,93 | -1,12 | -1,04 | -3,55 | -2,70 | -0,70 | -0,70 | -2,43 |
| **9** | -1,26 | 0,87 | 0,87 | -0,26 | -1,71 | 0,00 | -0,01 | -1,52 | -2,44 | -1,11 | -1,12 | -2,92 | -3,18 | -0,87 | -0,87 | -2,04 |
| **10** | -1,52 | 0,84 | 0,84 | -0,17 | -1,88 | -0,06 | -0,07 | -0,63 | -2,59 | -1,17 | -1,22 | -2,05 | -3,40 | -0,97 | -0,97 | -1,36 |
| **11** | -1,24 | 0,55 | 0,86 | 0,31 | -1,76 | -0,93 | -0,14 | 0,10 | -2,54 | -2,00 | -1,24 | -0,94 | -2,93 | -1,85 | -1,04 | -0,82 |
| **12** | -0,88 | -0,04 | 0,87 | 0,97 | -1,41 | -1,69 | -0,10 | -0,15 | -2,35 | -3,00 | -1,24 | -1,44 | -2,45 | -2,36 | -0,89 | -0,95 |
| **13** | -0,64 | -0,49 | 0,83 | 0,82 | -0,99 | -2,13 | 0,01 | -0,13 | -1,93 | -3,63 | -1,24 | -1,48 | -2,40 | -2,73 | -0,83 | -0,99 |
| **14** | -0,30 | -0,53 | 0,93 | 0,80 | -0,66 | -2,39 | 0,06 | -0,02 | -1,48 | -4,04 | -1,19 | -1,27 | -1,82 | -2,42 | -0,77 | -0,84 |
| **15** | 0,67 | 0,38 | 1,14 | 0,92 | -0,18 | -2,38 | 0,15 | 0,14 | -1,03 | -4,21 | -1,49 | -1,09 | -0,99 | -1,64 | -0,62 | -0,62 |
| **16** | 1,37 | 1,43 | 1,25 | 1,11 | 0,33 | -1,27 | 0,13 | 0,29 | -1,06 | -3,68 | -1,86 | -1,02 | -0,31 | -0,05 | -0,43 | -0,39 |
| **17** | 1,14 | 1,21 | 1,21 | 1,22 | 0,59 | 1,29 | 0,51 | 0,55 | -1,08 | -2,10 | -1,39 | -0,89 | -0,44 | -0,53 | -0,38 | -0,38 |
| **18** | 1,09 | 1,11 | 1,18 | 1,21 | 0,63 | 0,20 | 0,69 | 0,66 | -0,78 | -0,07 | -0,36 | -0,73 | -0,44 | -0,61 | -0,40 | -0,43 |
| **19** | 1,15 | 1,13 | 1,19 | 1,20 | 0,61 | 0,75 | 0,73 | 0,71 | -0,64 | -1,20 | -0,74 | -0,68 | -0,36 | -0,30 | -0,34 | -0,34 |
| **20** | 1,19 | 1,18 | 1,21 | 1,20 | 0,71 | 0,84 | 0,69 | 0,72 | -0,62 | -0,58 | -0,79 | -0,65 | -0,28 | -0,19 | -0,26 | -0,26 |
| **21** | 1,21 | 1,21 | 1,21 | 1,20 | 0,79 | 0,79 | 0,74 | 0,76 | -0,60 | -0,43 | -0,67 | -0,60 | -0,23 | -0,19 | -0,22 | -0,22 |
| **22** | 1,21 | 1,21 | 1,21 | 1,21 | 0,83 | 0,80 | 0,82 | 0,82 | -0,54 | -0,64 | -0,55 | -0,54 | -0,20 | -0,20 | -0,19 | -0,19 |
| **23** | 1,15 | 1,15 | 1,15 | 1,15 | 0,82 | 0,81 | 0,83 | 0,83 | -0,51 | -0,60 | -0,50 | -0,51 | -0,19 | -0,20 | -0,20 | -0,20 |
| **24** | 1,09 | 1,09 | 1,09 | 1,09 | 0,81 | 0,81 | 0,82 | 0,81 | -0,49 | -0,52 | -0,49 | -0,49 | -0,19 | -0,20 | -0,20 | -0,20 |

**Table A5**. Hourly heat flux (W/ m^2^) for geometry 5 of novel double-skin façade (DSF) controlled-temperature building element

| **Season/ Heat flux [W/m^2^]** | **Winter** | | | | **Spring** | | | | **Summer** | | | | **Autumn** | | | |
| --- | --- | --- | --- | --- | --- | --- | --- | --- | --- | --- | --- | --- | --- | --- | --- | --- |
| **Time [hr]** | **Azimuth [°]** | | | | | | | | | | | | | | | |
|  | **0** | **90** | **180** | **270** | **0** | **90** | **180** | **270** | **0** | **90** | **180** | **270** | **0** | **90** | **180** | **270** |
| **1** | 3,32 | 3,16 | 2,66 | 2,80 | 2,30 | 3,09 | 1,95 | 1,97 | 0,46 | 1,81 | 0,66 | 0,37 | 0,37 | 0,44 | -0,13 | -0,04 |
| **2** | 4,56 | 4,44 | 3,28 | 3,37 | 2,37 | 2,97 | 2,02 | 2,08 | 0,32 | 1,29 | 0,50 | 0,25 | 0,10 | 0,14 | -0,27 | -0,22 |
| **3** | 3,43 | 3,32 | 2,77 | 2,84 | 2,03 | 2,49 | 1,87 | 1,92 | 0,63 | 1,54 | 0,69 | 0,58 | 0,28 | 0,30 | -0,09 | -0,04 |
| **4** | 2,42 | 2,33 | 1,94 | 1,99 | 1,69 | 2,00 | 1,64 | 1,66 | 0,66 | 1,39 | 0,06 | -0,56 | 0,40 | 0,41 | 0,07 | 0,10 |
| **5** | 1,90 | 1,84 | 1,60 | 1,64 | -0,47 | -0,27 | -2,22 | -13,97 | -2,01 | -1,59 | -7,99 | -20,06 | -0,35 | -0,12 | -0,31 | -1,34 |
| **6** | 1,19 | 1,54 | 1,43 | 1,54 | -5,46 | -3,04 | -4,22 | -29,39 | -4,28 | -4,03 | -12,13 | -31,06 | -7,61 | -1,70 | -1,80 | -18,72 |
| **7** | -9,65 | 0,25 | 0,19 | 0,55 | -12,39 | -4,39 | -3,97 | -24,12 | -6,64 | -5,03 | -6,75 | -25,92 | -14,89 | -2,93 | -3,00 | -22,35 |
| **8** | -14,40 | -1,20 | -1,25 | -13,29 | -17,18 | -4,09 | -3,76 | -12,86 | -11,57 | -4,74 | -2,11 | -17,10 | -19,72 | -3,79 | -3,85 | -16,61 |
| **9** | -17,65 | -1,88 | -1,93 | -14,30 | -17,46 | -4,02 | -4,18 | -3,72 | -14,95 | -4,05 | -1,46 | -6,43 | -20,96 | -4,75 | -4,79 | -4,95 |
| **10** | -16,96 | -1,69 | -1,72 | -9,50 | -14,50 | -3,72 | -3,78 | 4,54 | -14,34 | -3,61 | -2,28 | 5,44 | -19,01 | -4,96 | -4,99 | 4,53 |
| **11** | -11,94 | -3,47 | -0,96 | -1,57 | -9,50 | -8,91 | -3,49 | 12,28 | -11,44 | -8,60 | -2,62 | 15,24 | -11,00 | -10,80 | -4,51 | 7,61 |
| **12** | -4,64 | -7,70 | -0,20 | 7,93 | -4,28 | -15,03 | -2,35 | 10,84 | -7,33 | -17,02 | -2,48 | 9,49 | -2,95 | -16,24 | -2,47 | 4,99 |
| **13** | 1,02 | -10,68 | 0,02 | 5,55 | 1,10 | -17,45 | -0,70 | 5,18 | -1,61 | -22,22 | -2,30 | 2,15 | 0,28 | -17,18 | -1,17 | 1,77 |
| **14** | 4,51 | -9,39 | 1,05 | 1,71 | 5,28 | -16,97 | 0,29 | 2,77 | 3,53 | -22,21 | -1,64 | 2,00 | 4,87 | -10,85 | -0,22 | 2,08 |
| **15** | 11,36 | -1,15 | 2,95 | 2,93 | 8,95 | -13,33 | 1,22 | 3,80 | 7,55 | -20,13 | -3,62 | 3,20 | 11,85 | 0,55 | 1,15 | 3,40 |
| **16** | 17,75 | 10,33 | 4,05 | 5,92 | 11,75 | -1,77 | 0,95 | 4,66 | 6,31 | -12,52 | -5,95 | 3,03 | 16,31 | 15,91 | 2,60 | 4,36 |
| **17** | 14,12 | 9,05 | 3,22 | 5,97 | 11,56 | 23,07 | 3,89 | 5,93 | 3,73 | 4,00 | -3,98 | 3,06 | 12,01 | 12,13 | 2,62 | 3,81 |
| **18** | 6,59 | 3,94 | 2,06 | 4,32 | 8,55 | 20,01 | 5,06 | 5,81 | 3,69 | 23,82 | 3,94 | 3,45 | 5,59 | 4,45 | 1,72 | 2,91 |
| **19** | 3,92 | 2,87 | 1,90 | 3,15 | 6,18 | 8,95 | 4,79 | 5,17 | 3,63 | 15,83 | 5,84 | 3,03 | 3,75 | 3,51 | 1,33 | 2,34 |
| **20** | 4,19 | 3,48 | 1,95 | 2,59 | 5,32 | 9,43 | 3,57 | 4,30 | 3,03 | 9,33 | 4,26 | 2,42 | 3,82 | 4,25 | 1,39 | 1,96 |
| **21** | 3,94 | 3,30 | 1,84 | 2,28 | 4,81 | 8,42 | 2,89 | 3,51 | 2,43 | 7,20 | 1,99 | 2,02 | 3,40 | 3,79 | 1,37 | 1,70 |
| **22** | 3,20 | 2,68 | 1,68 | 2,05 | 4,01 | 6,52 | 2,86 | 3,15 | 2,07 | 6,03 | 1,72 | 1,83 | 2,67 | 2,84 | 1,26 | 1,50 |
| **23** | 2,09 | 1,55 | 1,11 | 1,40 | 2,96 | 4,70 | 2,49 | 2,66 | 1,64 | 4,60 | 1,76 | 1,49 | 1,85 | 1,92 | 0,88 | 1,08 |
| **24** | 1,02 | 0,67 | 0,52 | 0,72 | 2,21 | 3,36 | 1,99 | 2,01 | 1,24 | 3,29 | 1,47 | 1,13 | 1,18 | 1,24 | 0,50 | 0,65 |

**Table A6**. Hourly heat flux (W/ m^2^) for geometry 6 of novel double-skin façade (DSF) controlled-temperature building element

| **Season/ Heat flux [W/m^2^]** | **Winter** | | | | **Spring** | | | | **Summer** | | | | **Autumn** | | | |
| --- | --- | --- | --- | --- | --- | --- | --- | --- | --- | --- | --- | --- | --- | --- | --- | --- |
| **Time [hr]** | **Azimuth [°]** | | | | | | | | | | | | | | | |
|  | **0** | **90** | **180** | **270** | **0** | **90** | **180** | **270** | **0** | **90** | **180** | **270** | **0** | **90** | **180** | **270** |
| **1** | 1,47 | 1,34 | 1,54 | 1,50 | 0,92 | 0,87 | 0,93 | 0,89 | -0,60 | -0,71 | -0,62 | -0,66 | -0,31 | -0,28 | -0,28 | -0,27 |
| **2** | 1,49 | 1,42 | 1,51 | 1,53 | 0,90 | 0,91 | 0,96 | 0,94 | -0,57 | -0,65 | -0,67 | -0,62 | -0,28 | -0,27 | -0,27 | -0,28 |
| **3** | 1,50 | 1,38 | 1,46 | 1,55 | 0,88 | 0,82 | 0,99 | 0,88 | -0,54 | -0,59 | -0,76 | -0,59 | -0,39 | -0,34 | -0,26 | -0,28 |
| **4** | 1,52 | 1,33 | 1,40 | 1,33 | 0,87 | 0,74 | 0,88 | 0,62 | -0,50 | -0,53 | -0,84 | -0,69 | -0,81 | -0,43 | -0,25 | -0,29 |
| **5** | 1,54 | 1,29 | 1,35 | 1,10 | 0,81 | 0,66 | 0,74 | 0,35 | -0,81 | -0,73 | -0,92 | -0,86 | -1,24 | -0,52 | -0,25 | -0,35 |
| **6** | 1,31 | 1,24 | 1,29 | 0,88 | 0,40 | 0,57 | 0,60 | 0,09 | -1,16 | -0,97 | -1,01 | -1,03 | -1,67 | -0,62 | -0,33 | -0,45 |
| **7** | 0,65 | 1,19 | 1,24 | 0,65 | -0,28 | 0,44 | 0,46 | -0,18 | -1,52 | -1,20 | -1,09 | -1,20 | -2,09 | -0,71 | -0,43 | -0,55 |
| **8** | -0,13 | 1,15 | 1,19 | 0,43 | -0,96 | 0,32 | 0,32 | -0,44 | -1,87 | -1,44 | -1,17 | -1,37 | -2,47 | -0,80 | -0,53 | -0,66 |
| **9** | -0,74 | 1,10 | 1,17 | 0,20 | -1,34 | 0,19 | 0,18 | -0,71 | -2,09 | -1,67 | -1,26 | -1,53 | -2,67 | -0,90 | -0,63 | -0,76 |
| **10** | -1,28 | 1,13 | 1,19 | -0,02 | -1,67 | 0,06 | 0,09 | -0,91 | -2,29 | -1,91 | -1,34 | -1,70 | -2,87 | -0,91 | -0,73 | -0,86 |
| **11** | -1,15 | 1,16 | 1,21 | 0,12 | -1,64 | 0,13 | 0,11 | -0,71 | -2,49 | -2,14 | -1,42 | -1,75 | -3,08 | -0,85 | -0,83 | -0,96 |
| **12** | -0,97 | 1,19 | 1,23 | 0,34 | -1,50 | 0,19 | 0,12 | -0,50 | -2,53 | -2,38 | -1,50 | -1,65 | -2,97 | -0,80 | -0,93 | -0,98 |
| **13** | -0,70 | 1,22 | 1,25 | 0,55 | -1,25 | 0,26 | 0,13 | -0,30 | -2,19 | -2,62 | -1,59 | -1,55 | -2,59 | -0,74 | -0,92 | -0,90 |
| **14** | -0,22 | 1,25 | 1,27 | 0,77 | -0,85 | 0,33 | 0,14 | -0,09 | -1,85 | -2,87 | -1,67 | -1,45 | -2,13 | -0,69 | -0,84 | -0,83 |
| **15** | 0,41 | 1,29 | 1,29 | 0,99 | -0,41 | 0,39 | 0,15 | 0,12 | -1,51 | -3,11 | -1,75 | -1,35 | -1,48 | -0,64 | -0,77 | -0,75 |
| **16** | 0,84 | 1,32 | 1,30 | 1,18 | -0,04 | 0,46 | 0,16 | 0,27 | -1,31 | -3,36 | -1,76 | -1,25 | -0,86 | -0,58 | -0,69 | -0,67 |
| **17** | 1,11 | 1,33 | 1,31 | 1,22 | 0,23 | 0,53 | 0,26 | 0,43 | -1,16 | -3,08 | -1,61 | -1,15 | -0,76 | -0,53 | -0,61 | -0,59 |
| **18** | 1,09 | 1,33 | 1,32 | 1,26 | 0,35 | 0,59 | 0,36 | 0,58 | -1,01 | -2,29 | -1,46 | -1,06 | -0,67 | -0,49 | -0,54 | -0,51 |
| **19** | 1,11 | 1,34 | 1,33 | 1,30 | 0,47 | 0,66 | 0,47 | 0,73 | -0,92 | -1,50 | -1,32 | -0,97 | -0,59 | -0,45 | -0,46 | -0,44 |
| **20** | 1,16 | 1,34 | 1,33 | 1,35 | 0,59 | 0,72 | 0,58 | 0,76 | -0,84 | -0,96 | -1,17 | -0,89 | -0,52 | -0,40 | -0,40 | -0,40 |
| **21** | 1,19 | 1,34 | 1,34 | 1,33 | 0,71 | 0,78 | 0,68 | 0,79 | -0,77 | -0,90 | -1,02 | -0,80 | -0,46 | -0,36 | -0,37 | -0,36 |
| **22** | 1,22 | 1,34 | 1,35 | 1,32 | 0,76 | 0,84 | 0,79 | 0,81 | -0,73 | -0,84 | -0,88 | -0,71 | -0,41 | -0,32 | -0,33 | -0,32 |
| **23** | 1,25 | 1,35 | 1,35 | 1,31 | 0,81 | 0,90 | 0,85 | 0,84 | -0,70 | -0,80 | -0,74 | -0,66 | -0,36 | -0,27 | -0,29 | -0,28 |
| **24** | 1,26 | 1,35 | 1,35 | 1,30 | 0,86 | 0,92 | 0,87 | 0,86 | -0,66 | -0,75 | -0,72 | -0,65 | -0,31 | -0,24 | -0,26 | -0,24 |

**Figure A1**. Hourly heat flux (W/ m^2^) for geometry 1 of novel double-skin façade (DSF) controlled-temperature building element

| **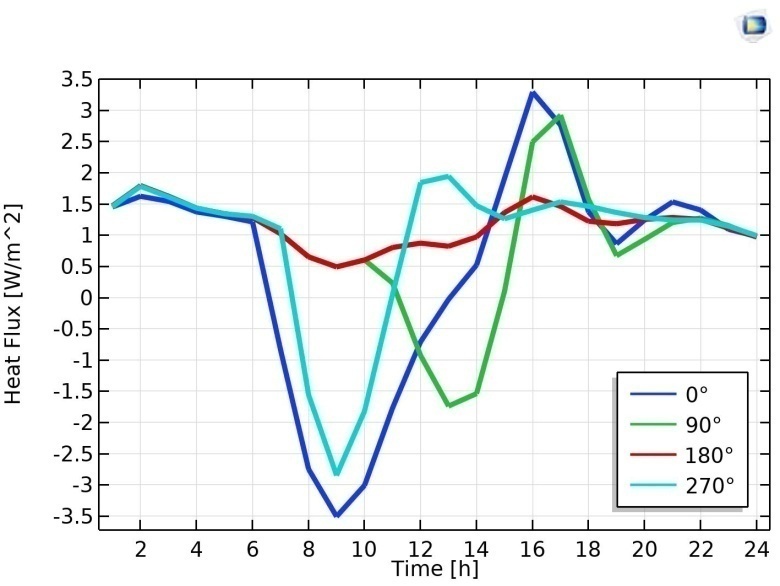**  **Winter** | **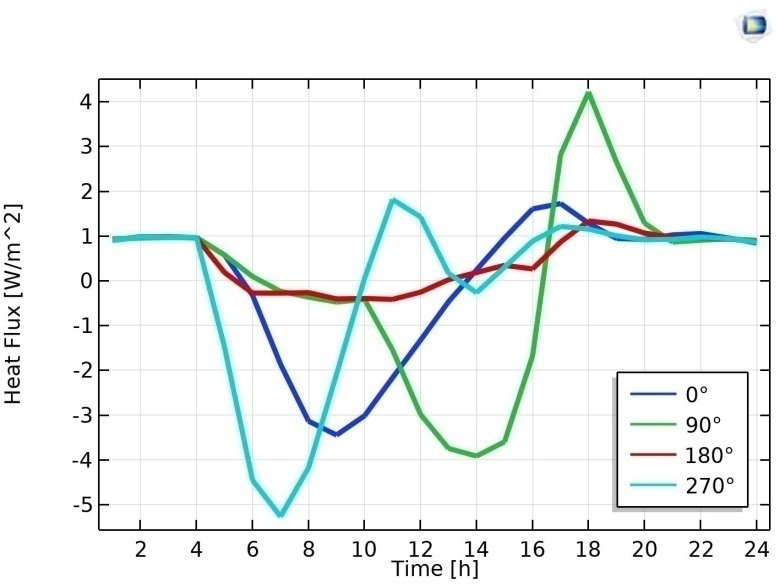**  **Spring** |
| --- | --- |
| **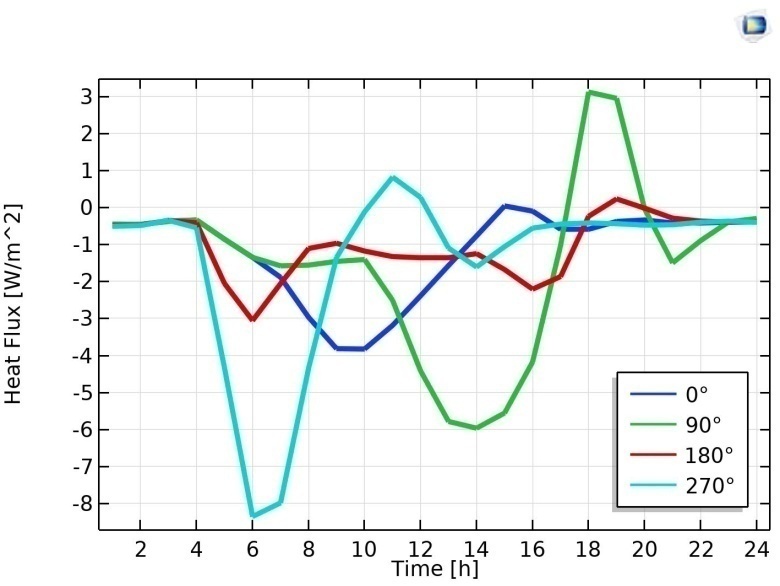**  **Summer** | **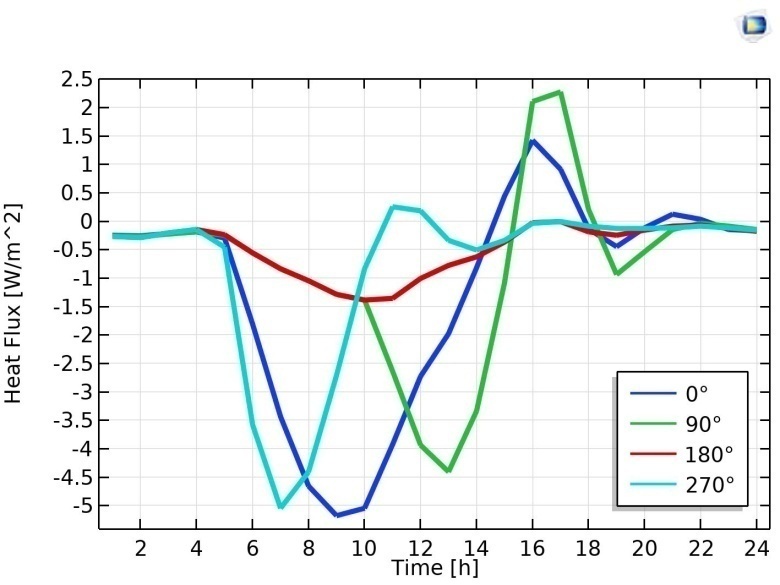**  **Autumn** |

**Figure A2**. Hourly heat flux (W/ m^2^) for geometry 2 of novel double-skin façade (DSF) controlled-temperature building element

| **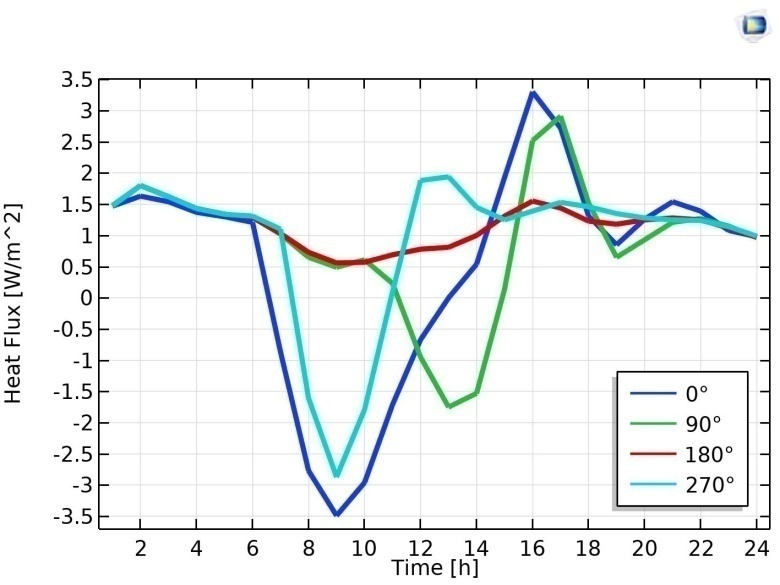**  **Winter** | **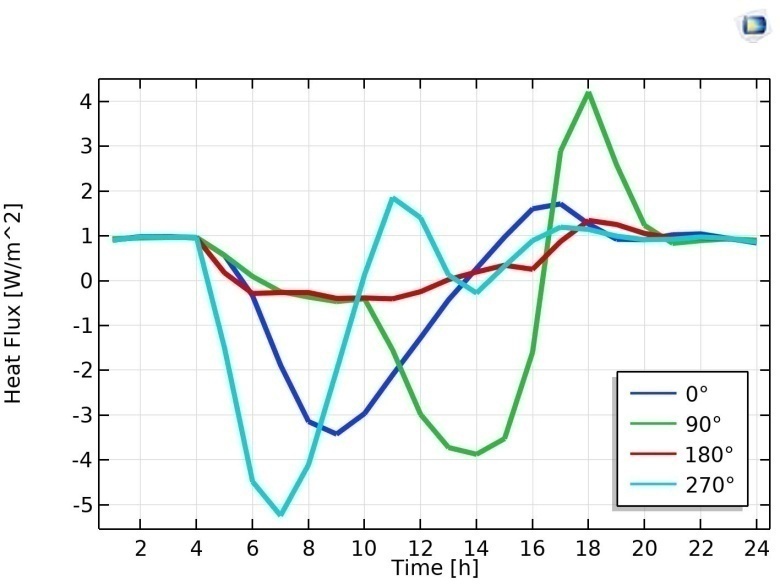**  **Spring** |
| --- | --- |
| **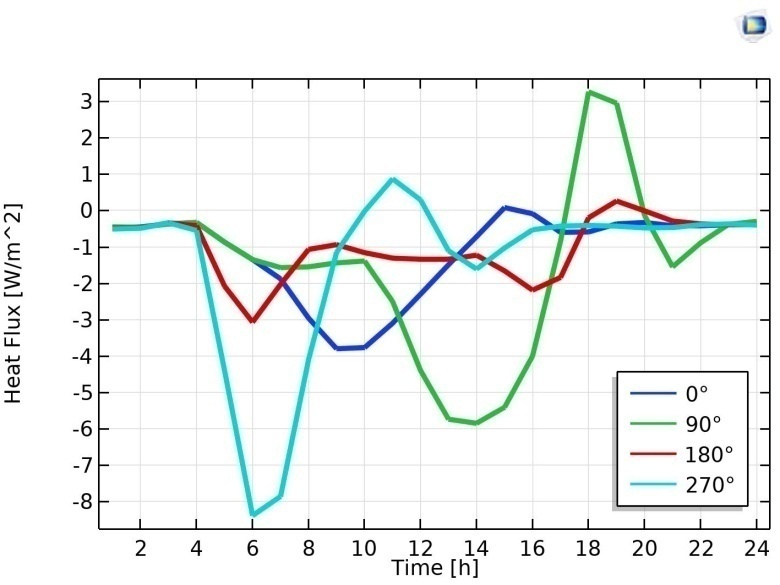**  **Summer** | **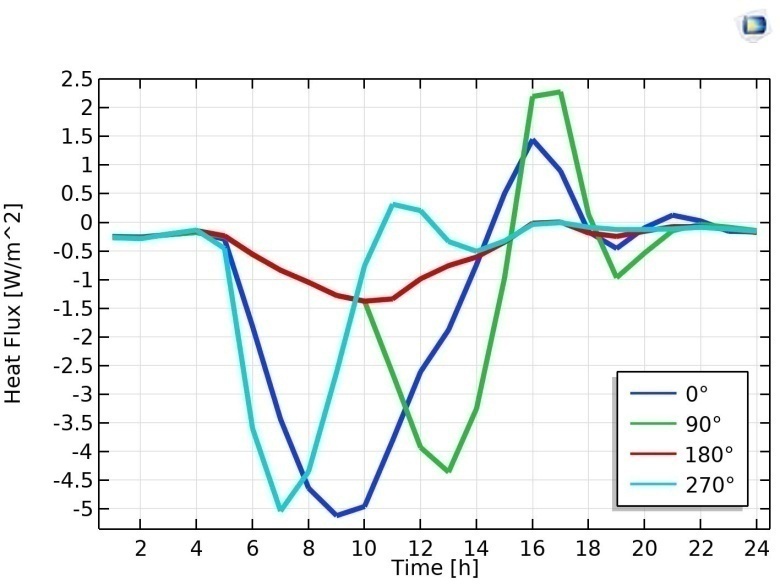**  **Autumn** |

**Figure A3**. Hourly heat flux (W/ m^2^) for geometry 3 of novel double-skin façade (DSF) controlled-temperature building element

| **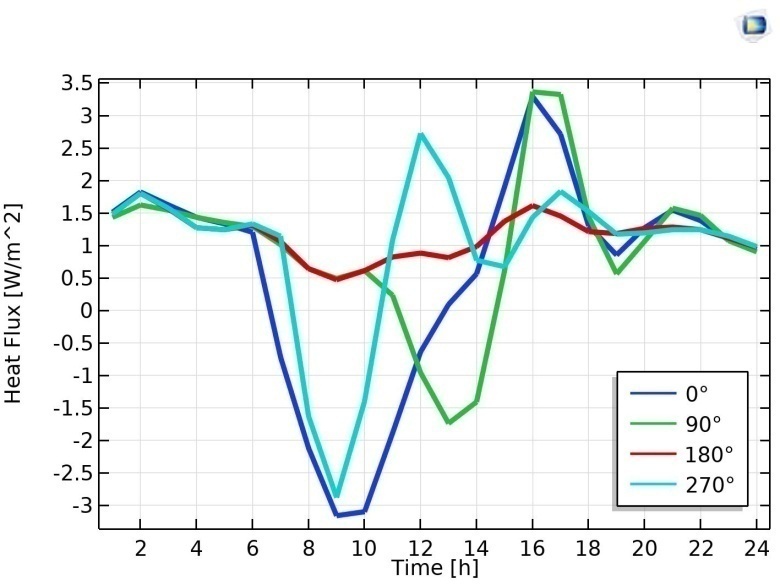**  **Winter** | **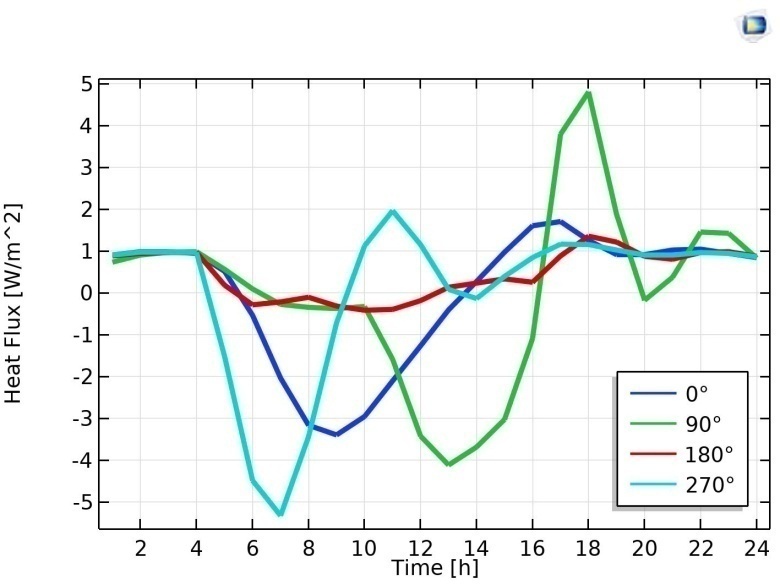**  **Spring** |
| --- | --- |
| **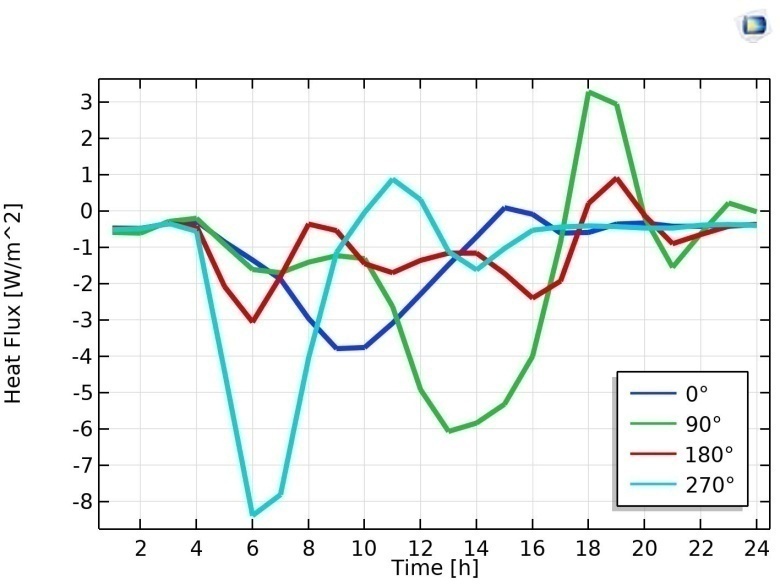**  **Summer** | **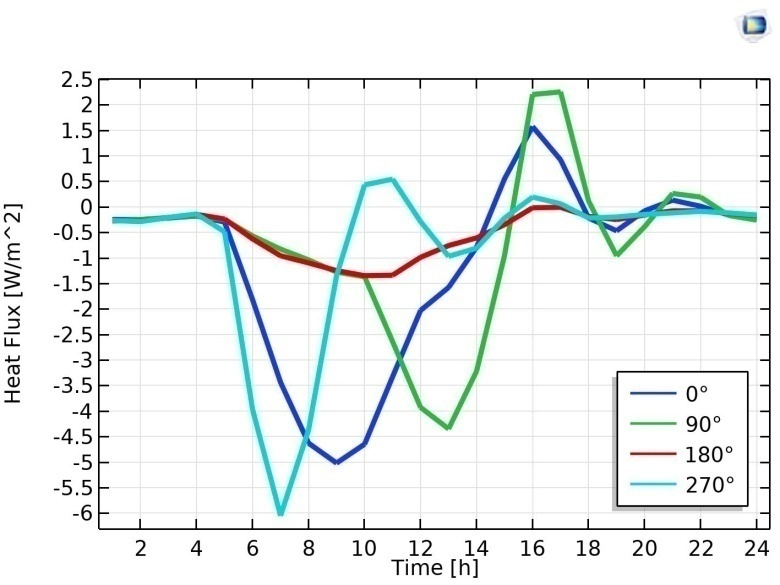**  **Autumn** |

**Figure A4**. Hourly heat flux (W/ m^2^) for geometry 4 of novel double-skin façade (DSF) controlled-temperature building element

| **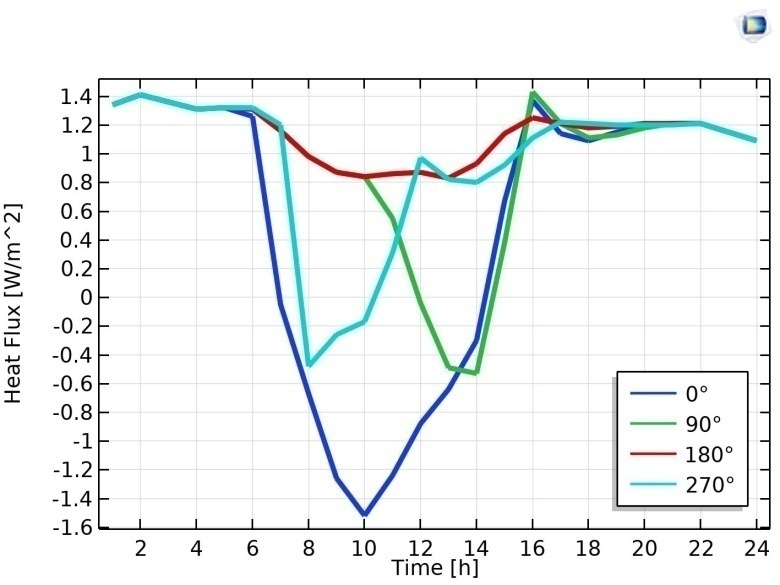**  **Winter** | **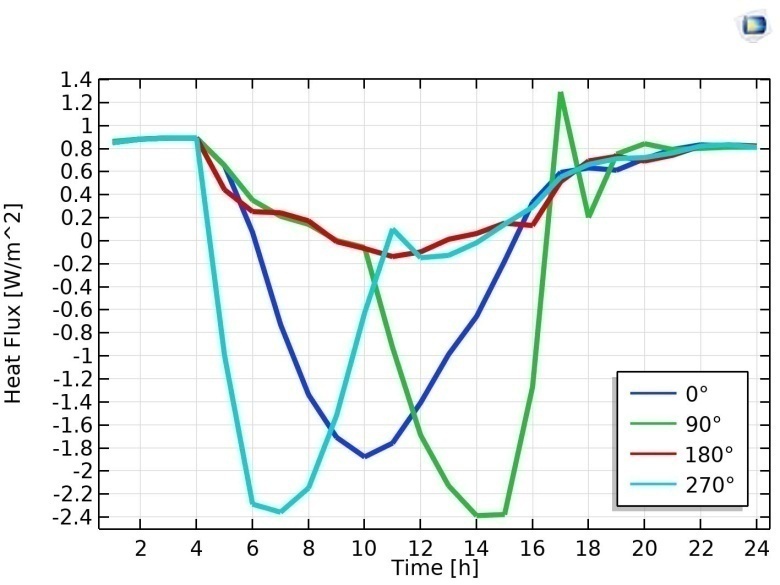**  **Spring** |
| --- | --- |
| **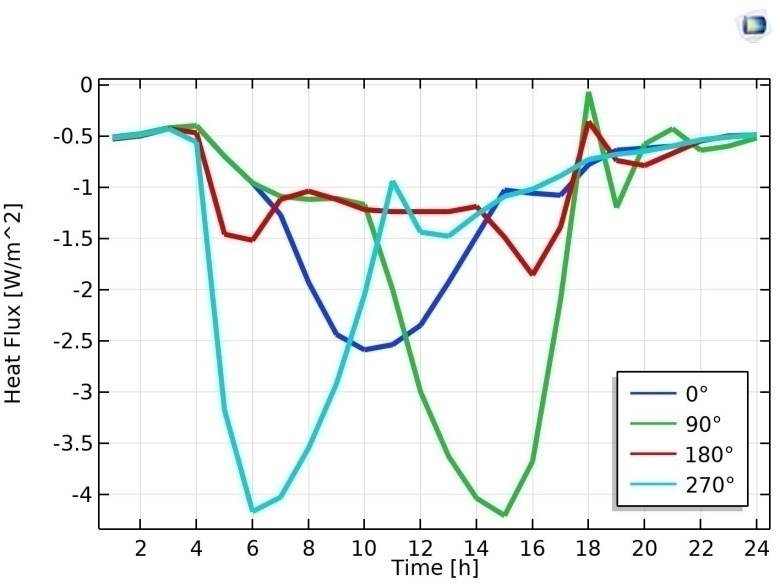**  **Summer** | **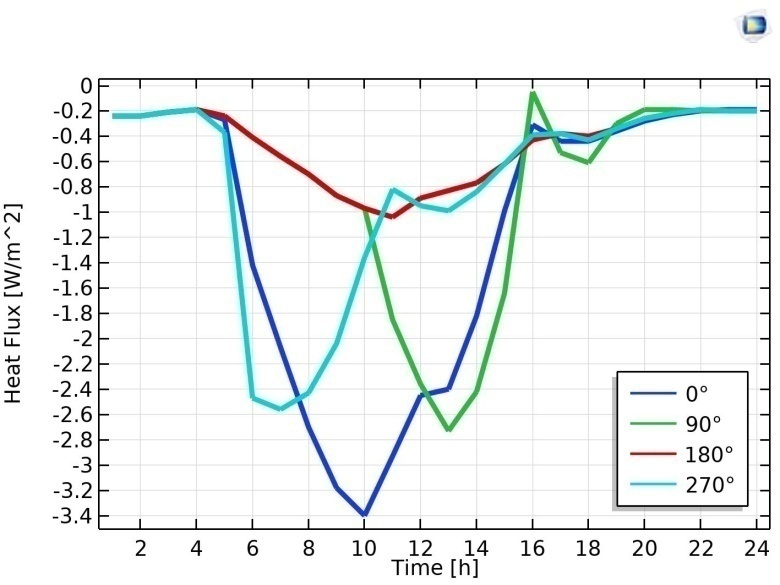**  **Autumn** |

**Figure A5**. Hourly heat flux (W/ m^2^) for geometry 5 of novel double-skin façade (DSF) controlled-temperature building element

| 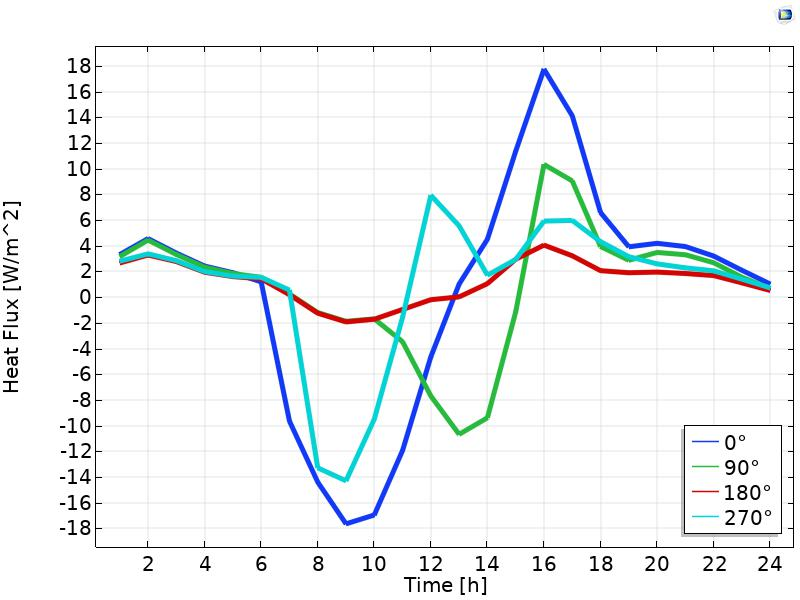  **Winter** | 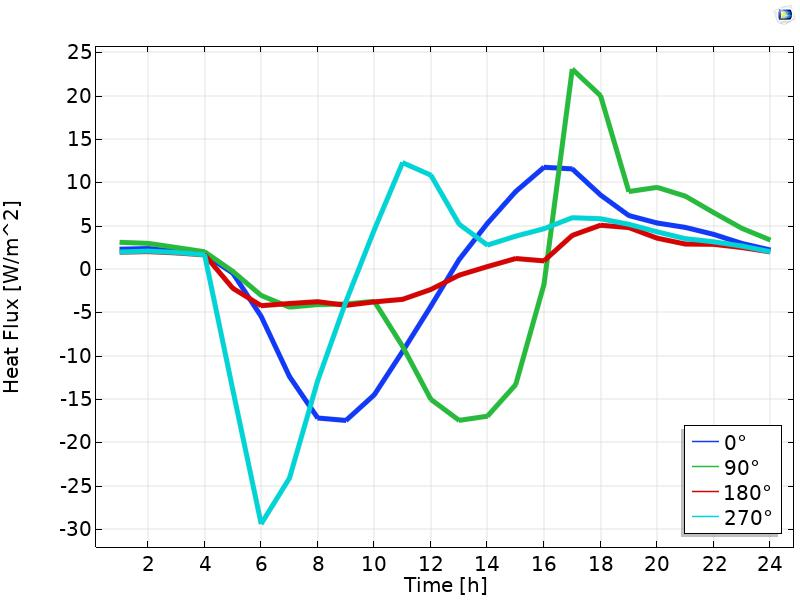  **Spring** |
| --- | --- |
| 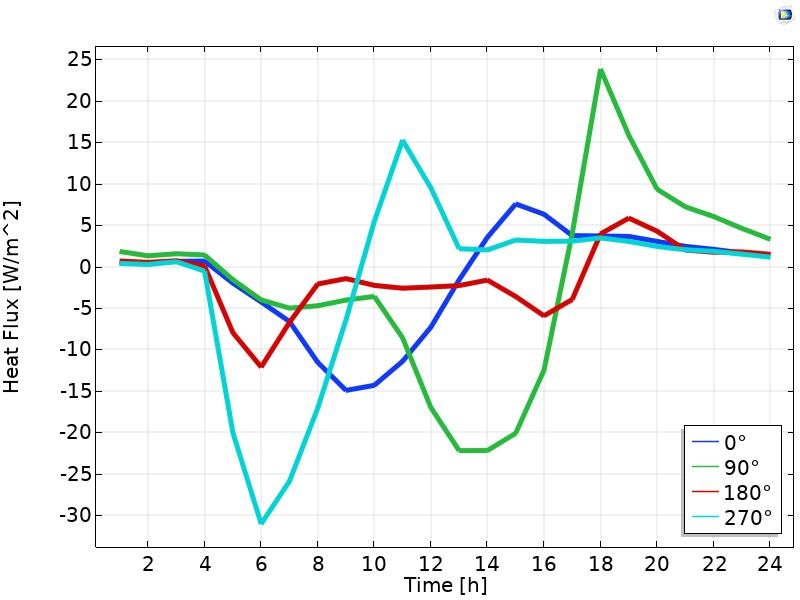  **Summer** | 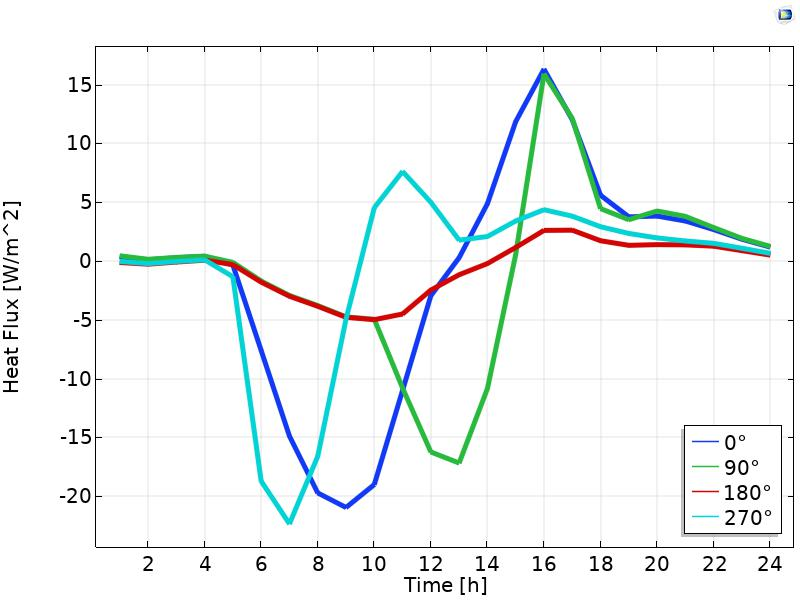  **Autumn** |

**Figure A6**. Hourly heat flux (W/ m^2^) for geometry 6 of novel double- skin façade (DSF) controlled-temperature building element

| **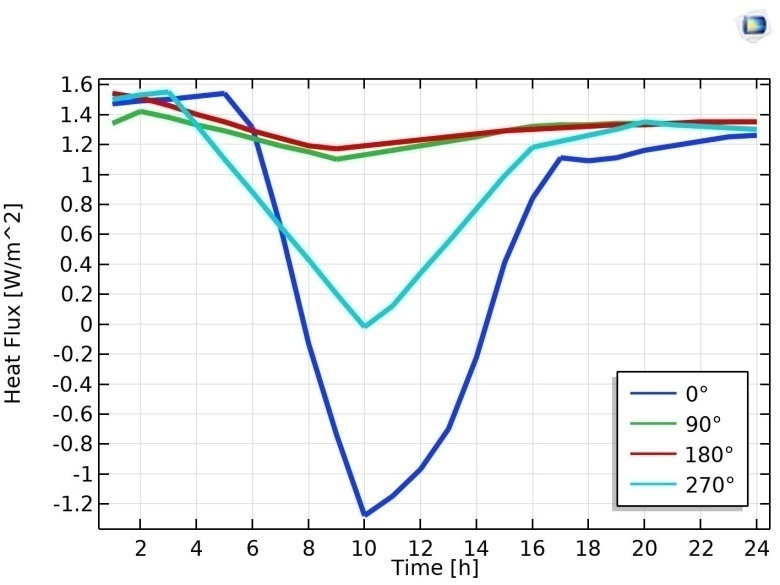**  **Winter** | **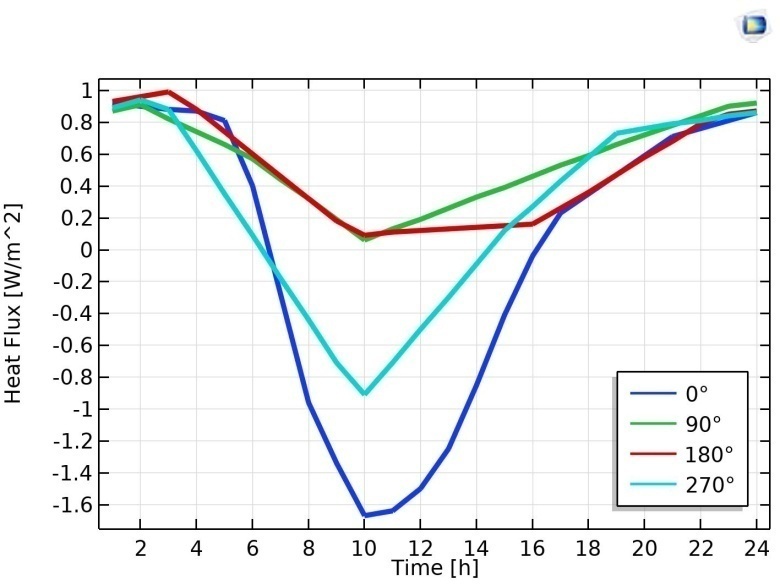**  **Spring** |
| --- | --- |
| **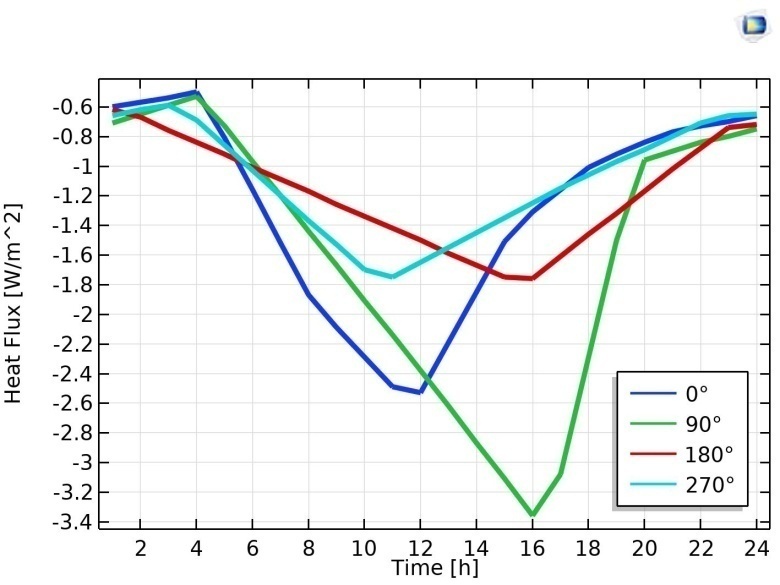**  **Summer** | **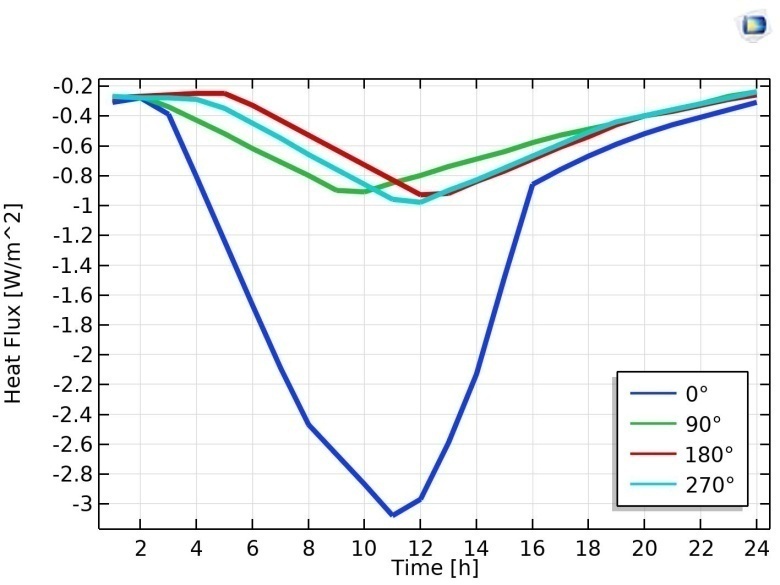**  **Autumn** |

**Figure B1**. Hourly building element temperature graphs (°C) for geometry 1 of novel double-skin façade (DSF) controlled-temperature building element – Winter

| **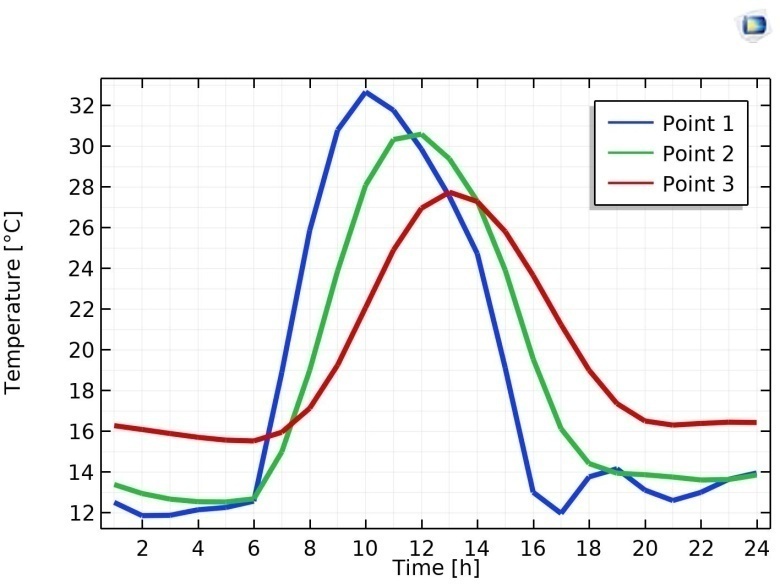**  **Azimuth 0°** | **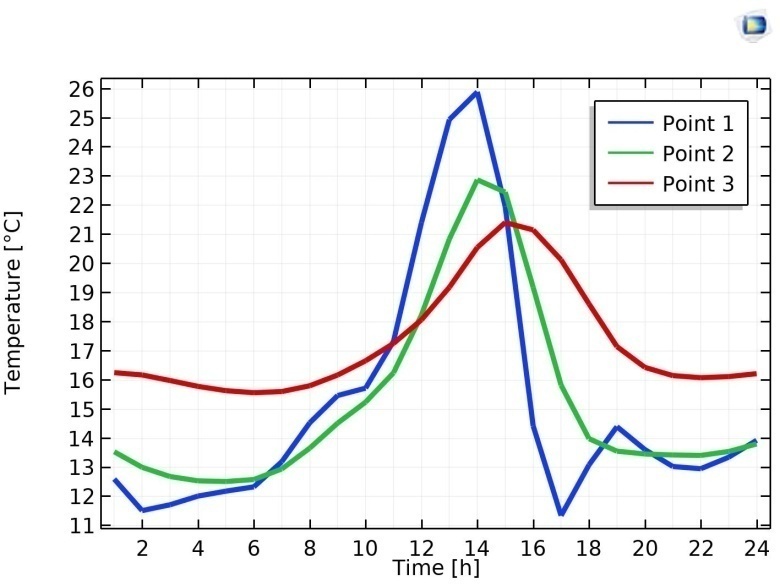**  **Azimuth 90°** |
| --- | --- |
| **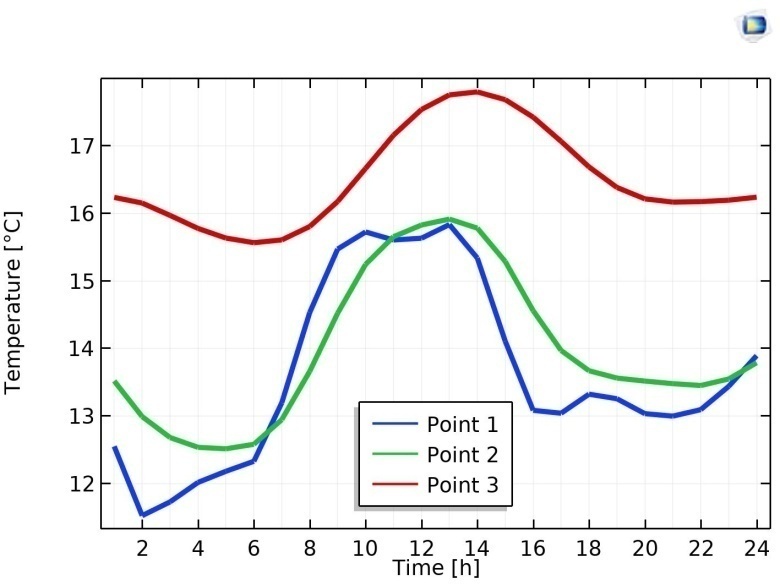**  **Azimuth 180°** | **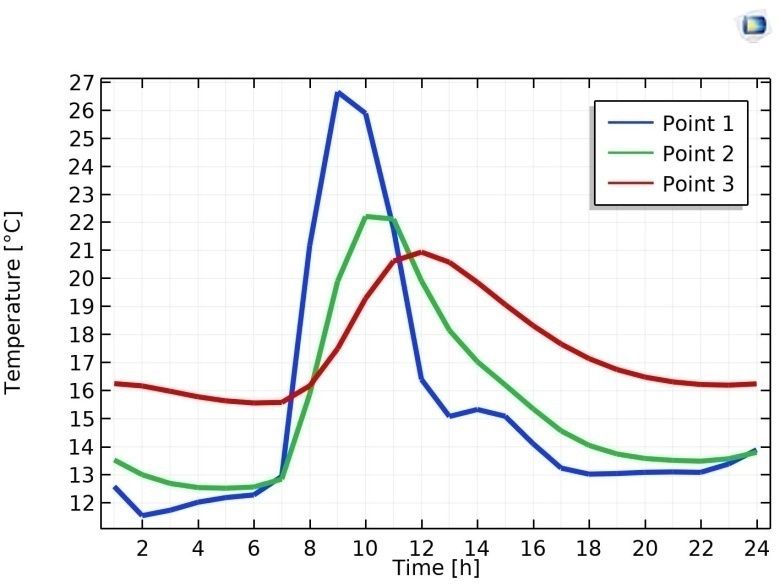**  **Azimuth 270°** |

**Figure B2**. Hourly building element temperature graphs (°C) for geometry 1 of novel double-skin façade (DSF) controlled-temperature building element – Spring

| **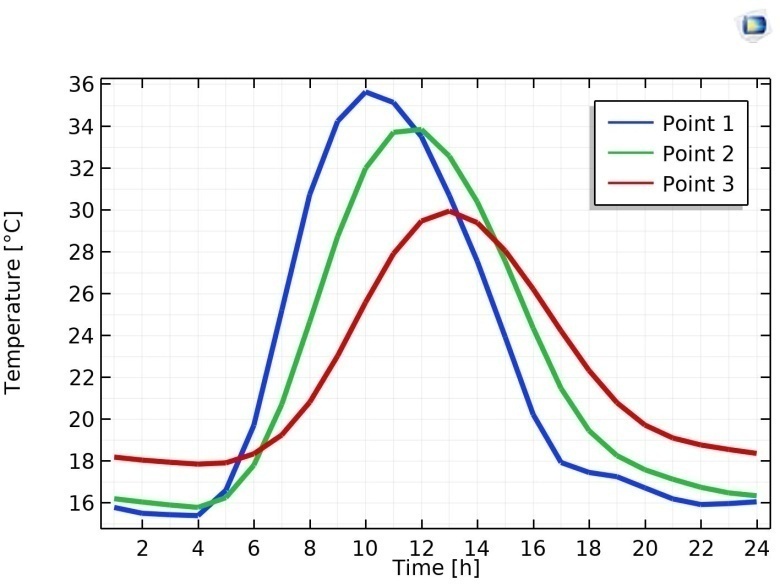**  **Azimuth 0°** | **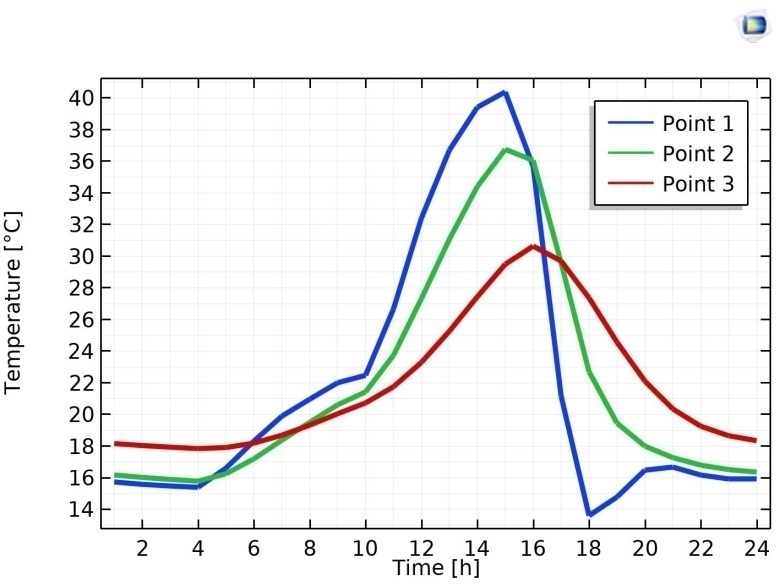**  **Azimuth 90°** |
| --- | --- |
| **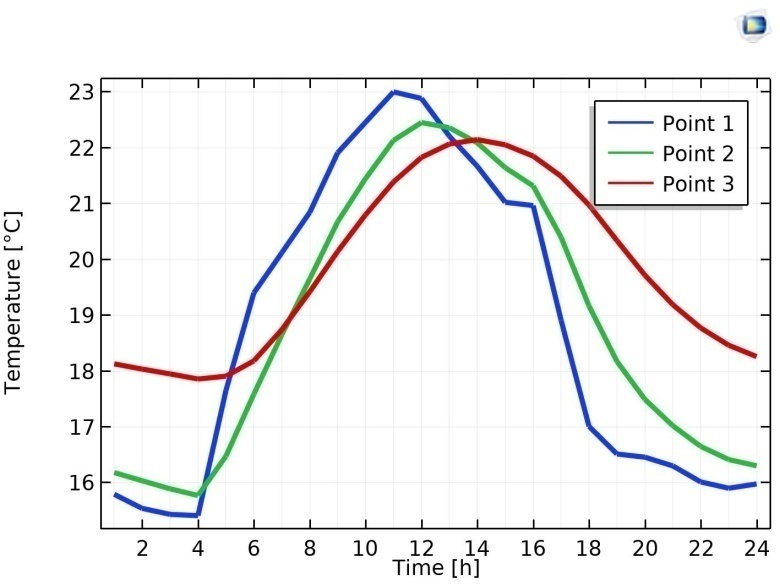**  **Azimuth 180°** | **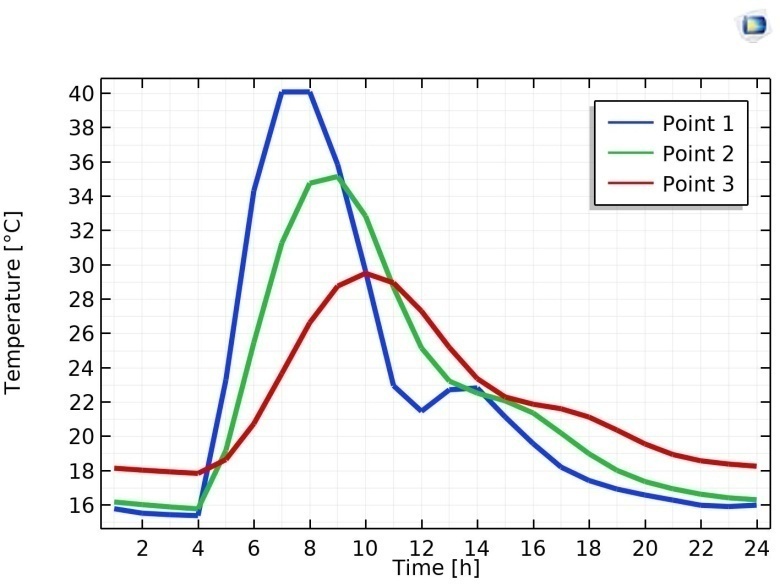**  **Azimuth 270°** |

**Figure B3**. Hourly building element temperature graphs (°C) for geometry 1 of novel double-skin façade (DSF) controlled-temperature building element – Summer

| **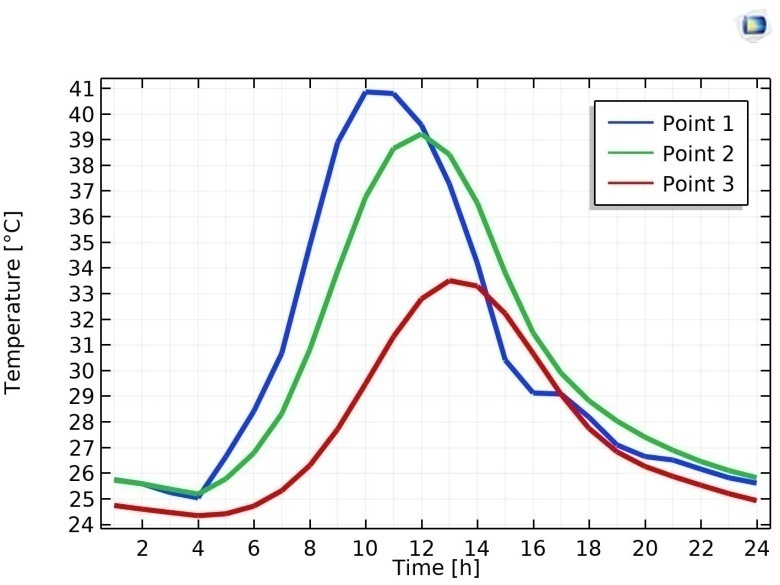**  **Azimuth 0°** | **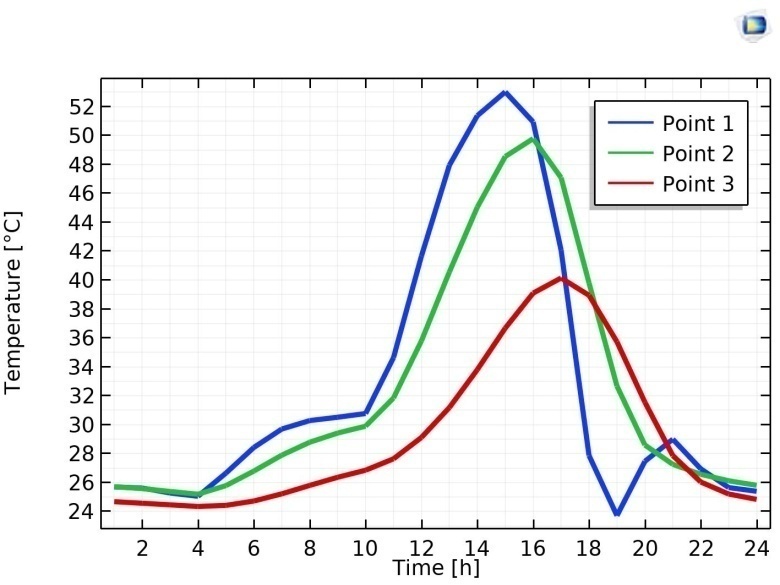**  **Azimuth 90°** |
| --- | --- |
| **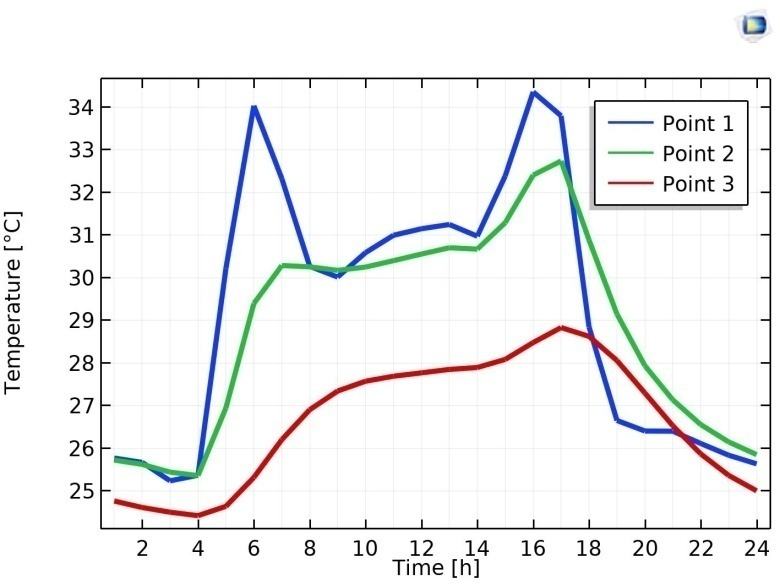**  **Azimuth 180°** | **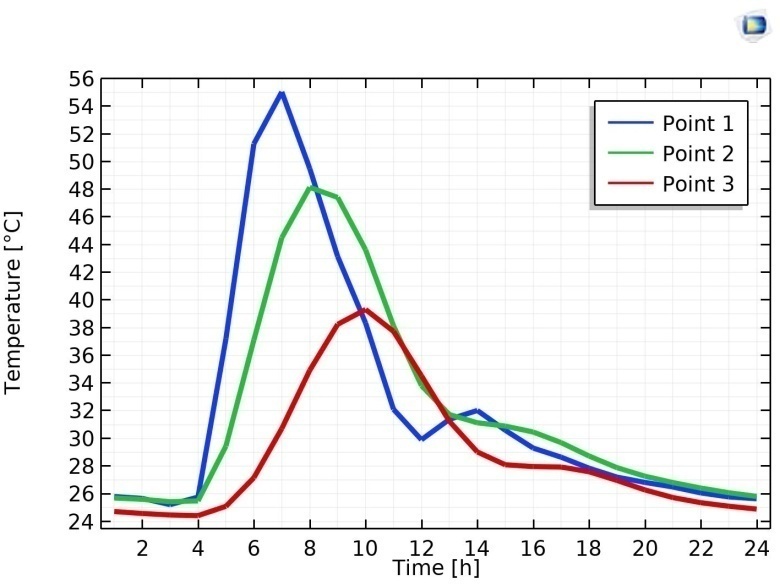**  **Azimuth 270°** |

**Figure B4**. Hourly building element temperature graphs (°C) for geometry 1 of novel double-skin façade (DSF) controlled-temperature building element – Autumn

| **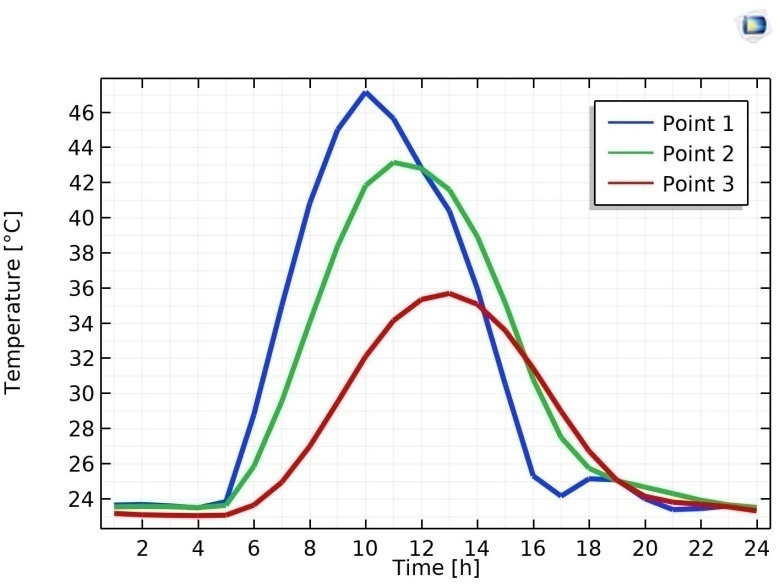**  **Azimuth 0°** | **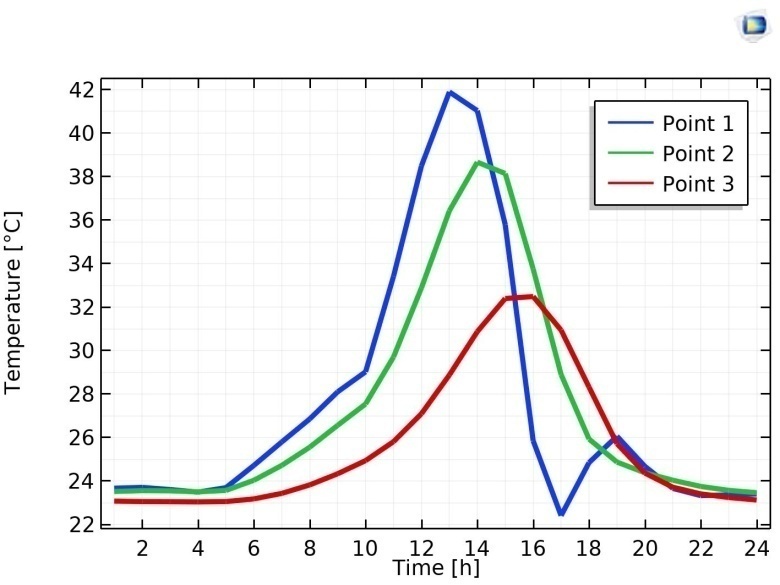**  **Azimuth 90°** |
| --- | --- |
| **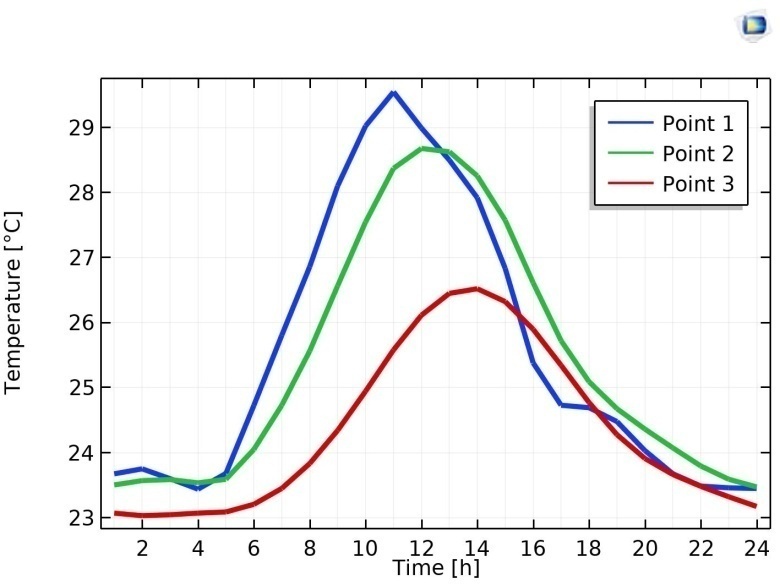**  **Azimuth 180°** | **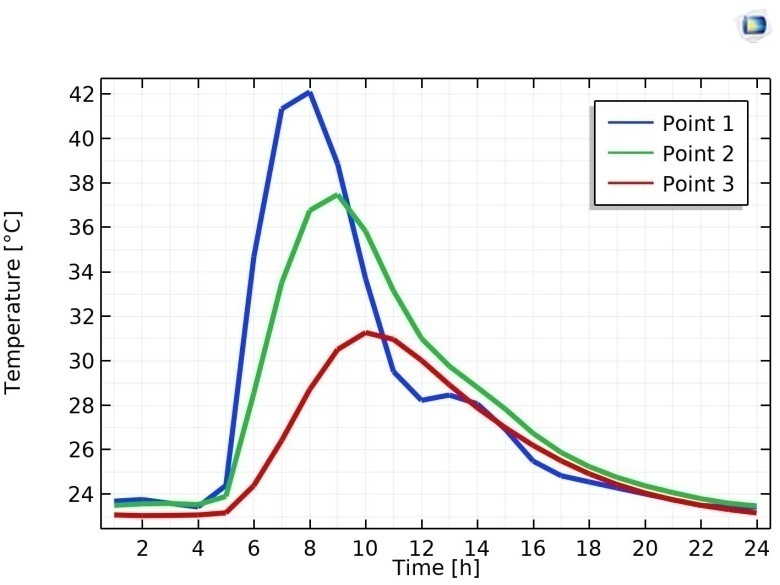**  **Azimuth 270°** |

**Figure B5**. Hourly building element temperature graphs (°C) for geometry 2 of novel double-skin façade (DSF) controlled-temperature building element – Winter

| **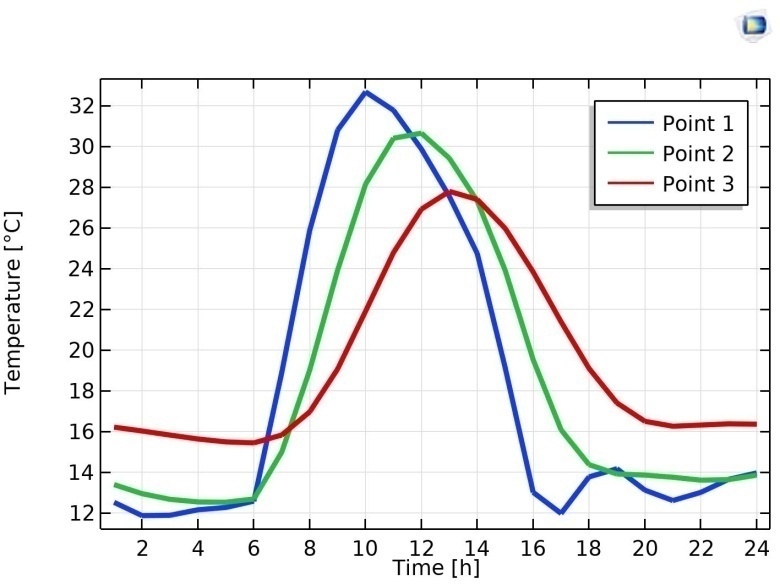**  **Azimuth 0°** | **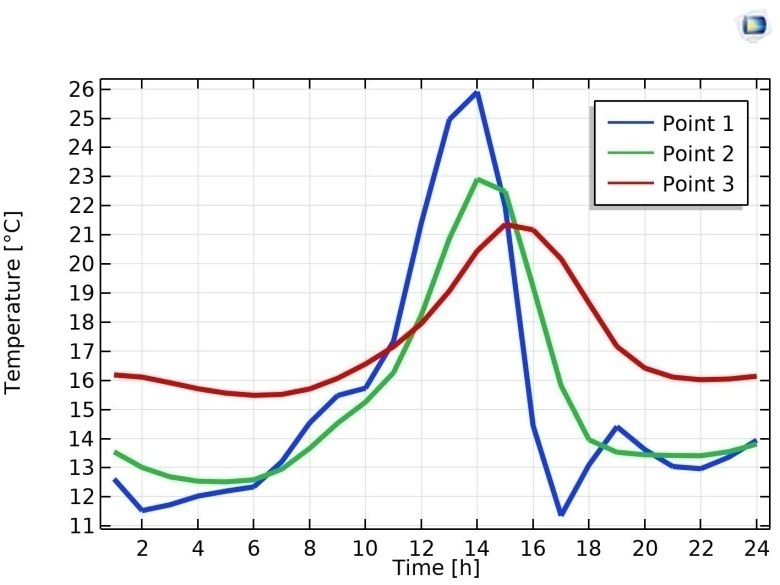**  **Azimuth 90°** |
| --- | --- |
| **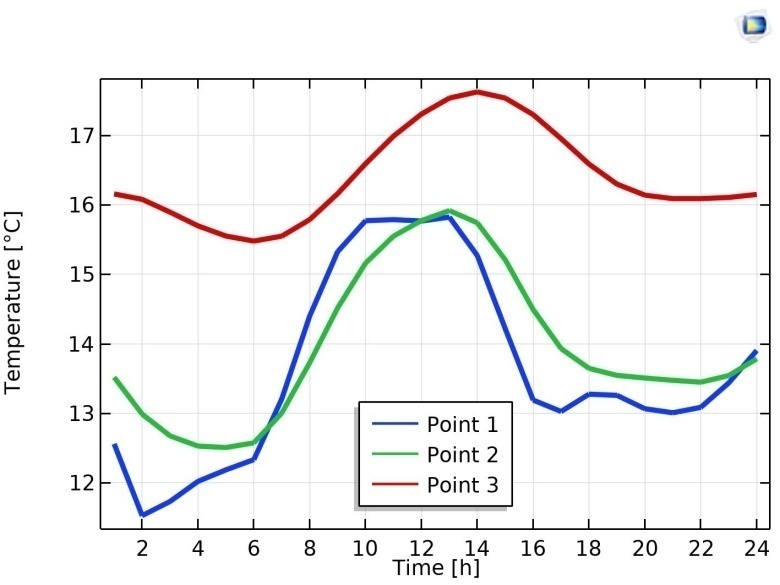**  **Azimuth 180°** | **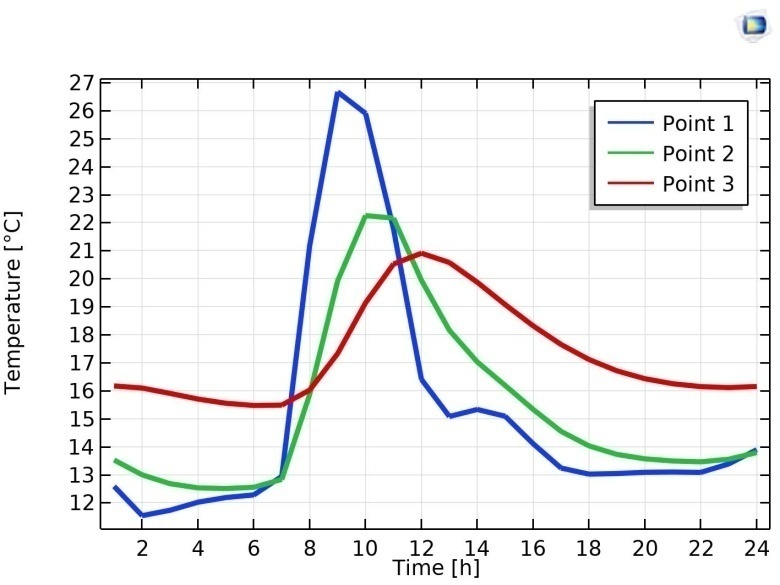**  **Azimuth 270°** |

**Figure B6**. Hourly building element temperature graphs (°C) for geometry 2 of novel double-skin façade (DSF) controlled-temperature building element – Spring

| **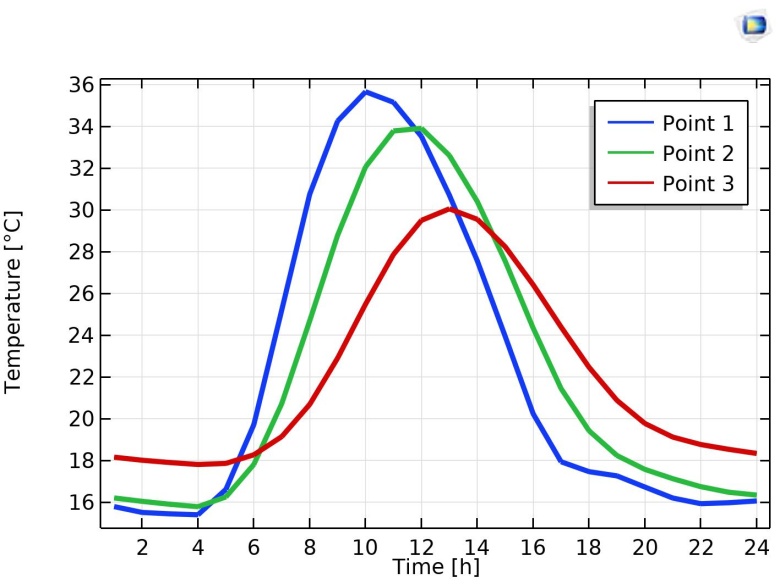**  **Azimuth 0°** | **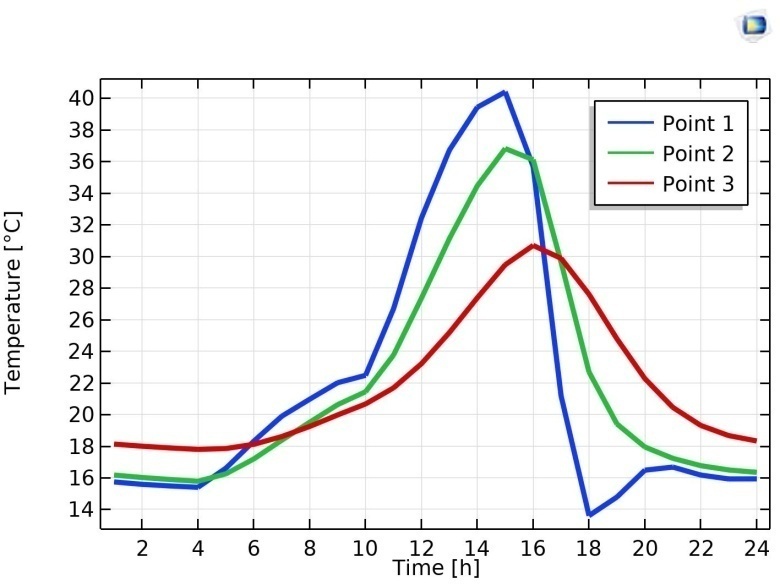**  **Azimuth 90°** |
| --- | --- |
| **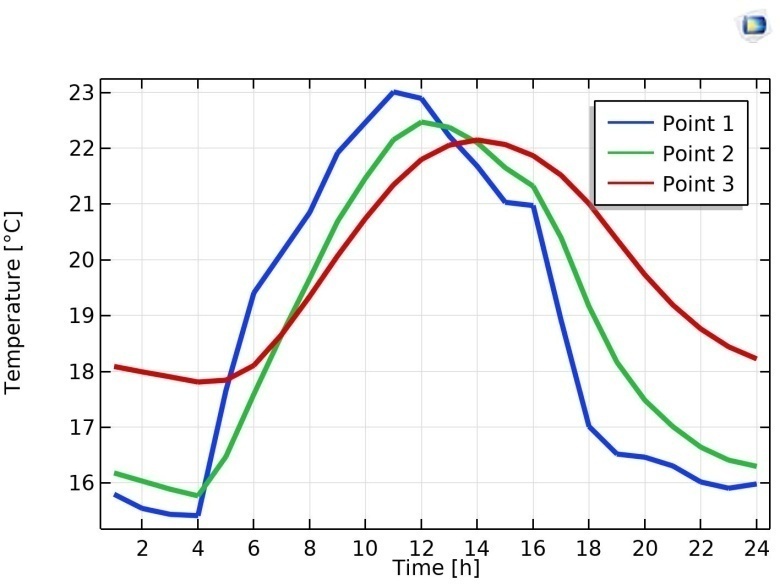**  **Azimuth 180°** | **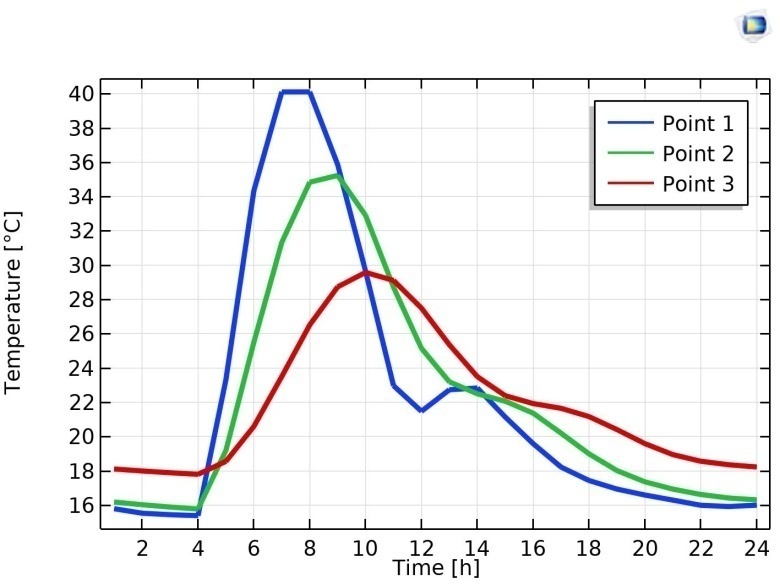**  **Azimuth 270°** |

**Figure B7**. Hourly building element temperature graphs (°C) for geometry 2 of novel double-skin façade (DSF) controlled-temperature building element – Summer

| **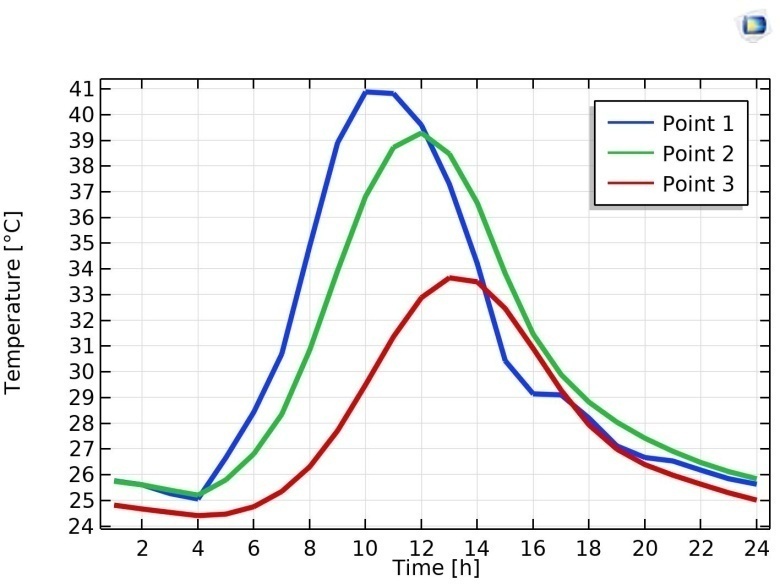**  **Azimuth 0°** | **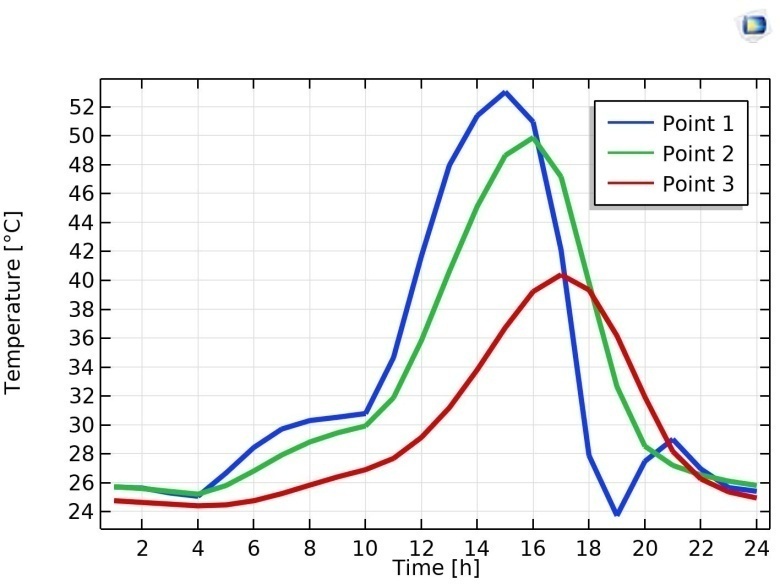**  **Azimuth 90°** |
| --- | --- |
| **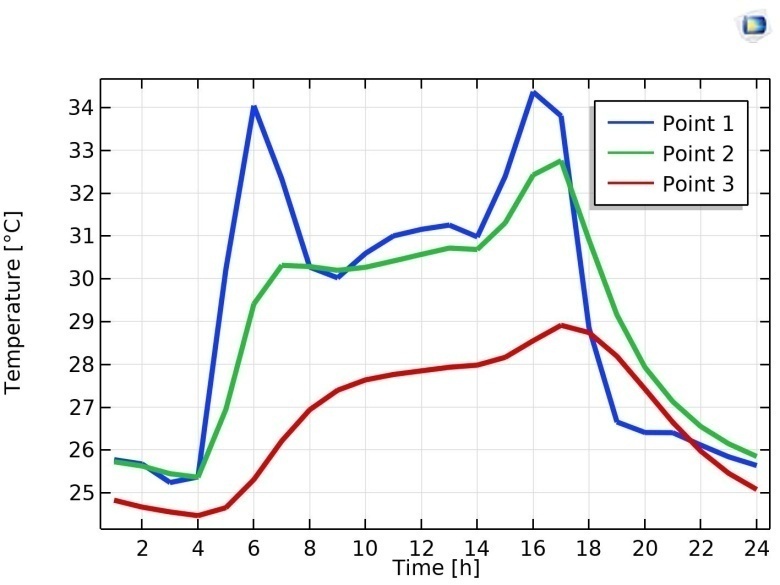**  **Azimuth 180°** | **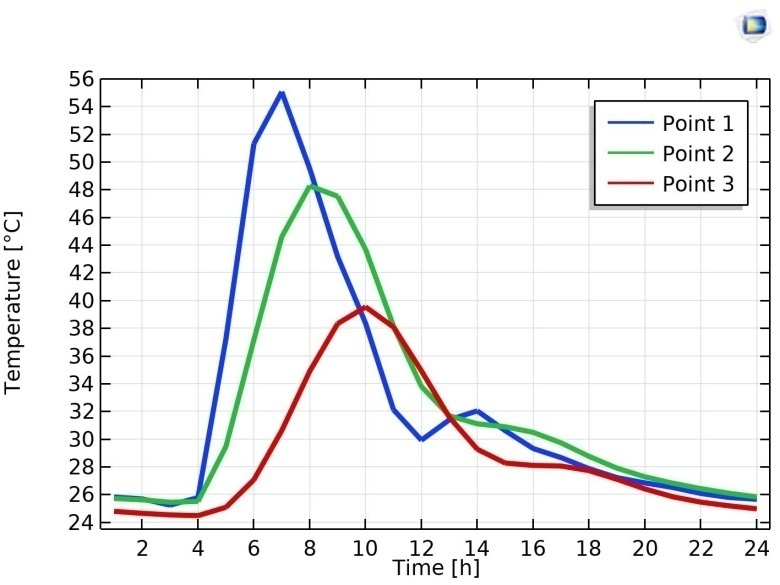**  **Azimuth 270°** |

**Figure B8**. Hourly building element temperature graphs (°C) for geometry 2 of novel double-skin façade (DSF) controlled-temperature building element – Autumn

| **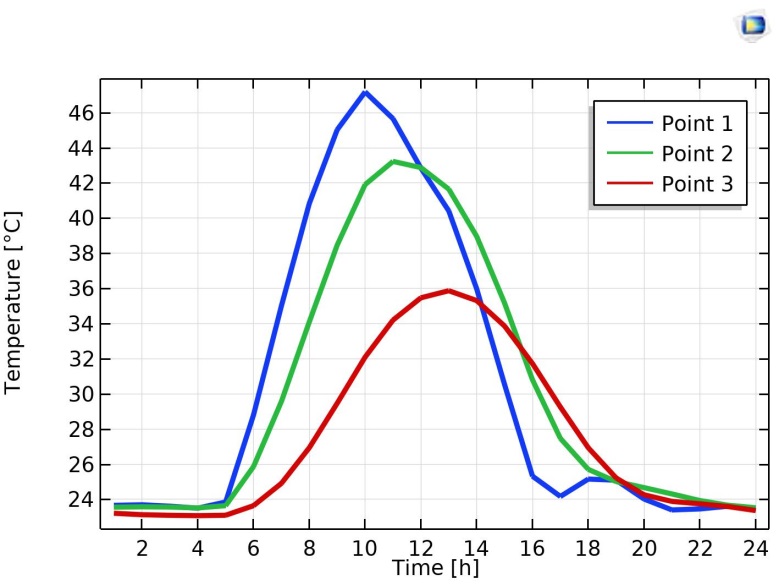**  **Azimuth 0°** | **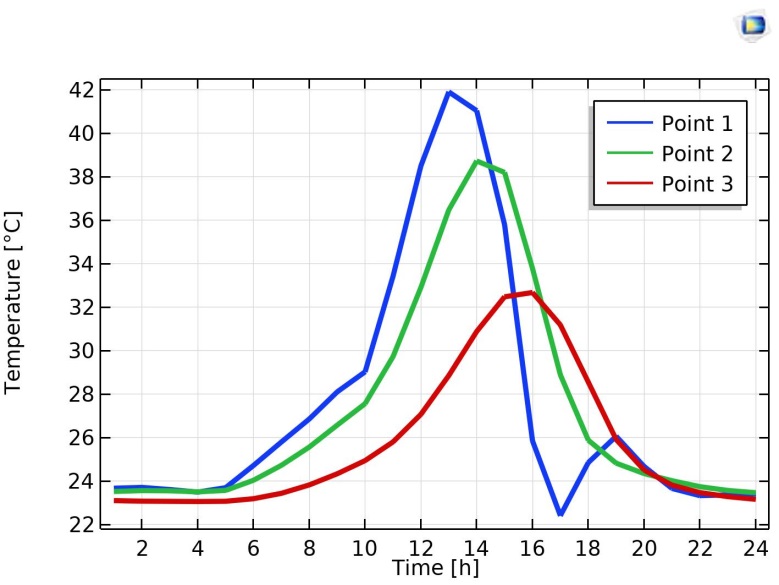**  **Azimuth 90°** |
| --- | --- |
| **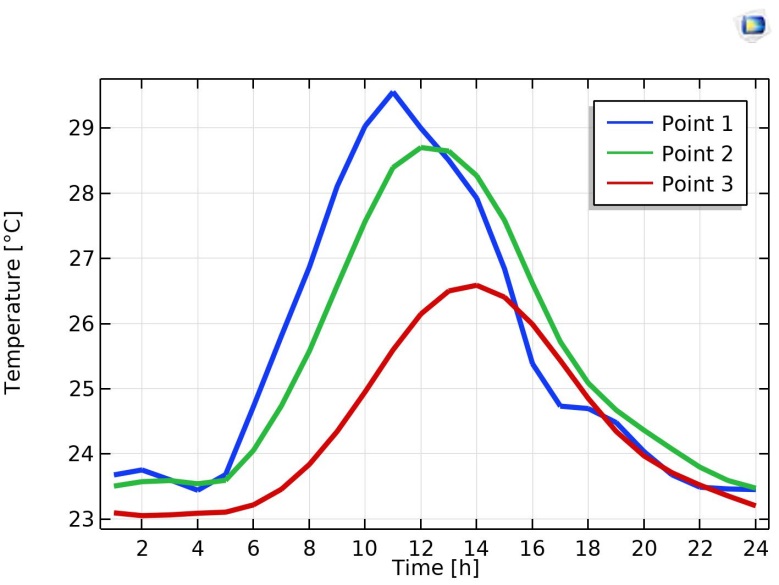**  **Azimuth 180°** | **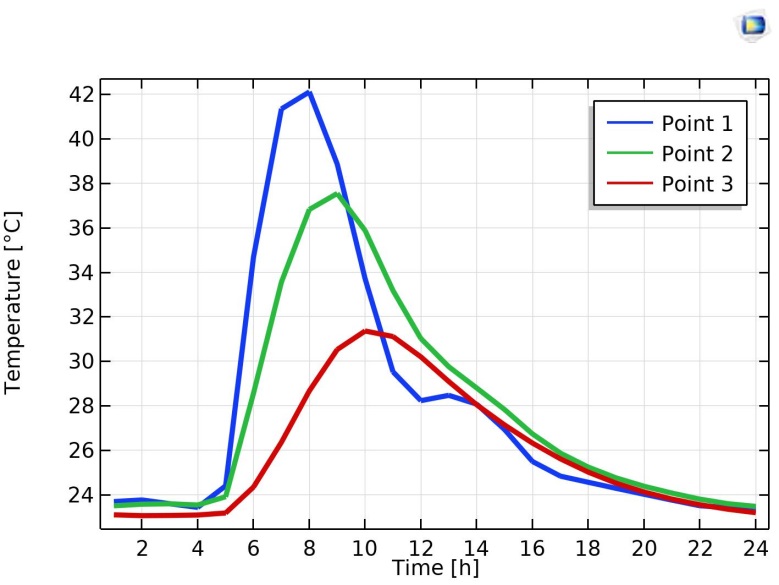**  **Azimuth 270°** |

**Figure B9**. Hourly building element temperature graphs (°C) for geometry 3 of novel double-skin façade (DSF) controlled-temperature building element – Winter

| **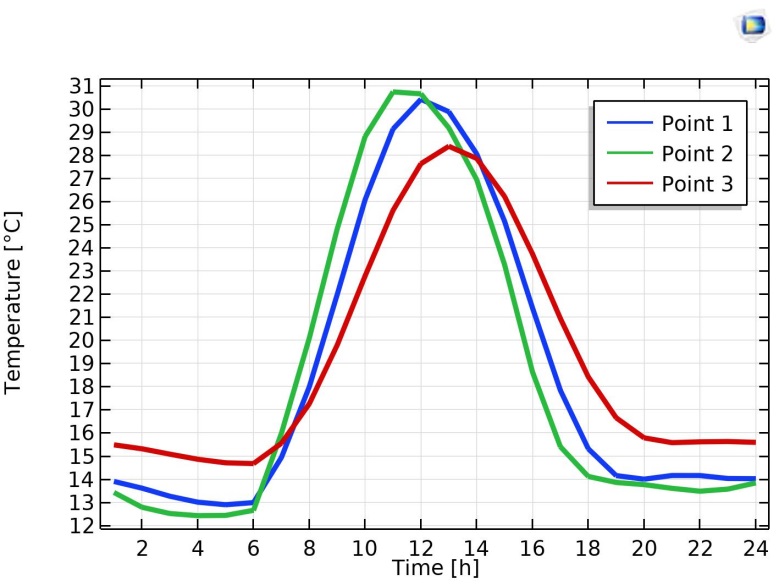**  **Azimuth 0°** | **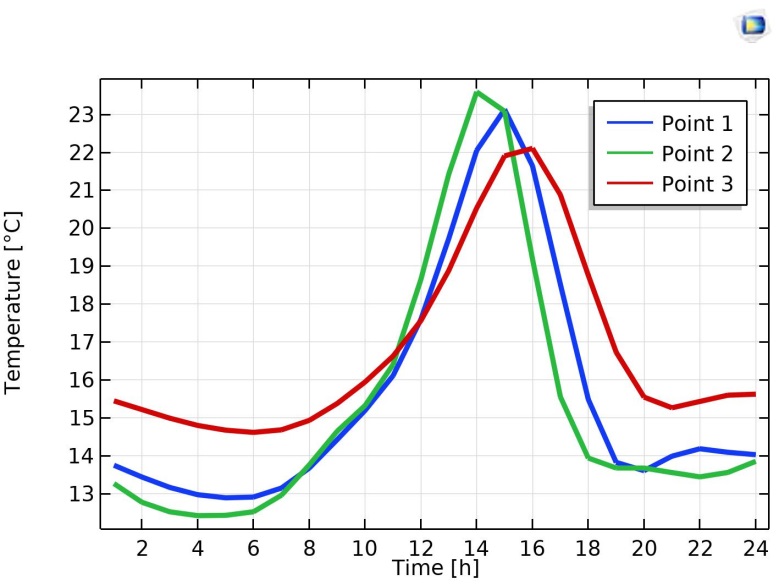**  **Azimuth 90°** |
| --- | --- |
| **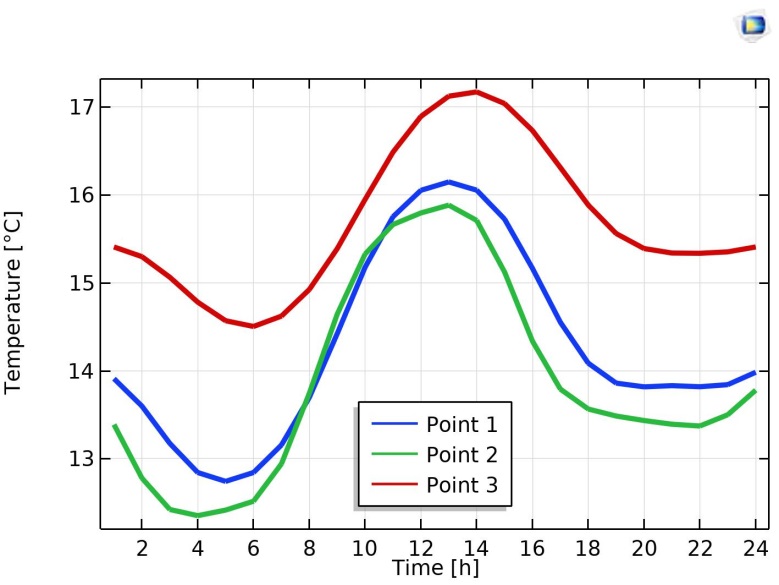**  **Azimuth 180°** | **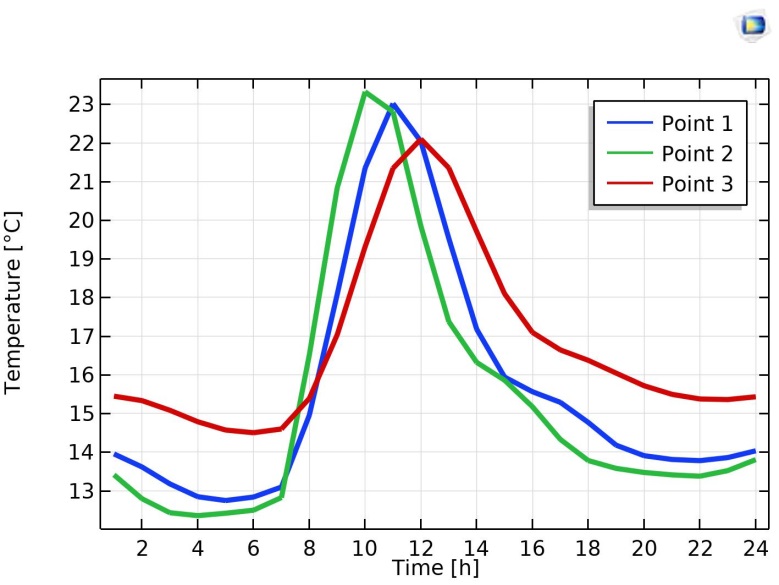**  **Azimuth 270°** |

**Figure B10**. Hourly building element temperature graphs (°C) for geometry 3 of novel double-skin façade (DSF) controlled-temperature building element – Spring

| **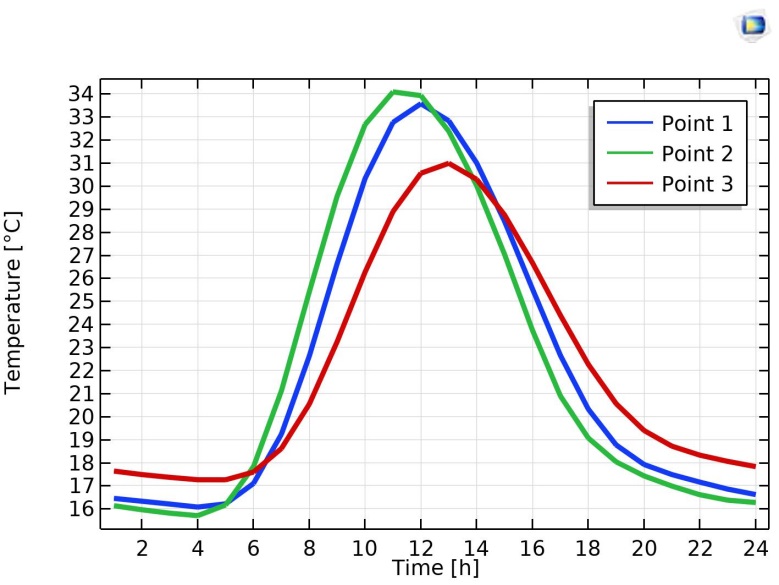**  **Azimuth 0°** | **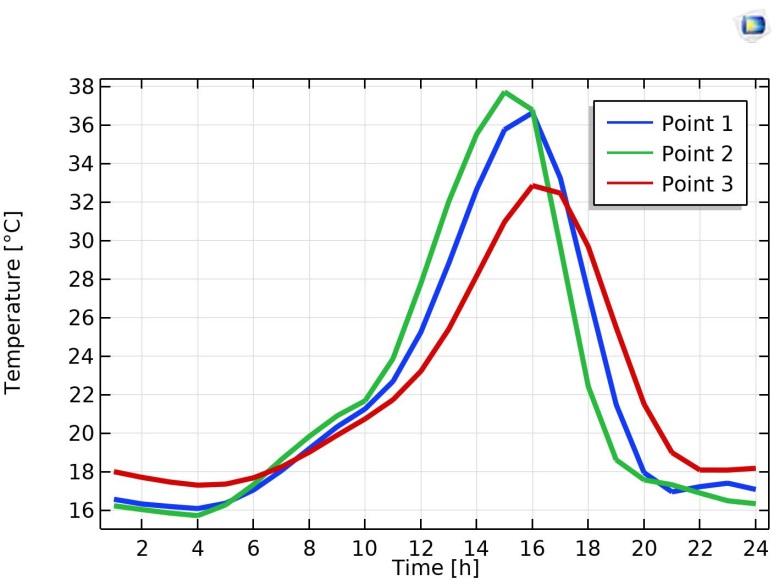**  **Azimuth 90°** |
| --- | --- |
| **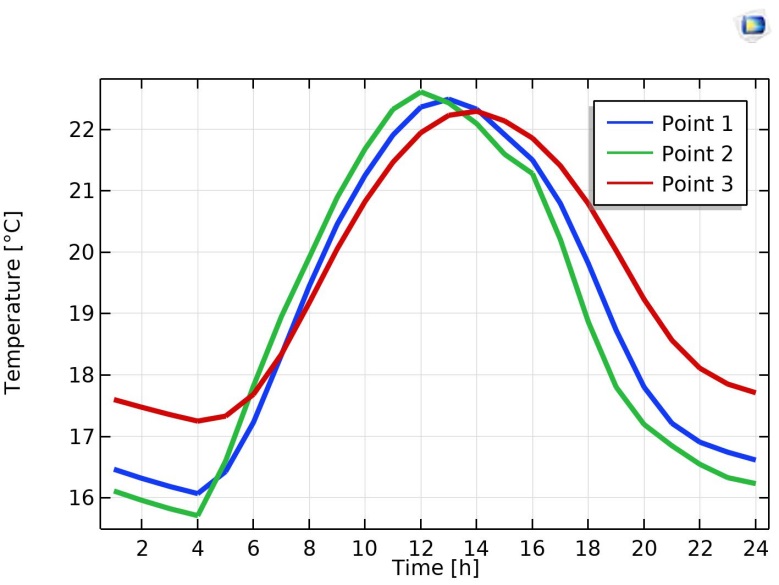**  **Azimuth 180°** | **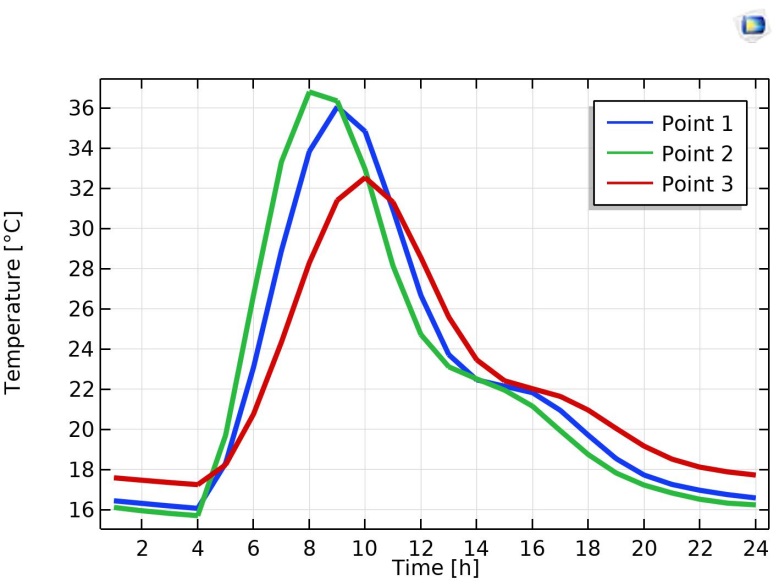**  **Azimuth 270°** |

**Figure B11**. Hourly building element temperature graphs (°C) for geometry 3 of novel double-skin façade (DSF) controlled-temperature building element – Summer

| **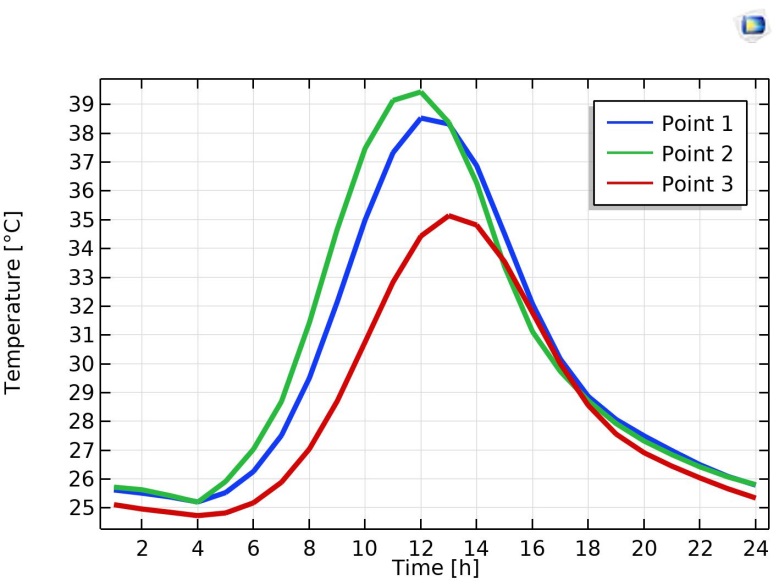**  **Azimuth 0°** | **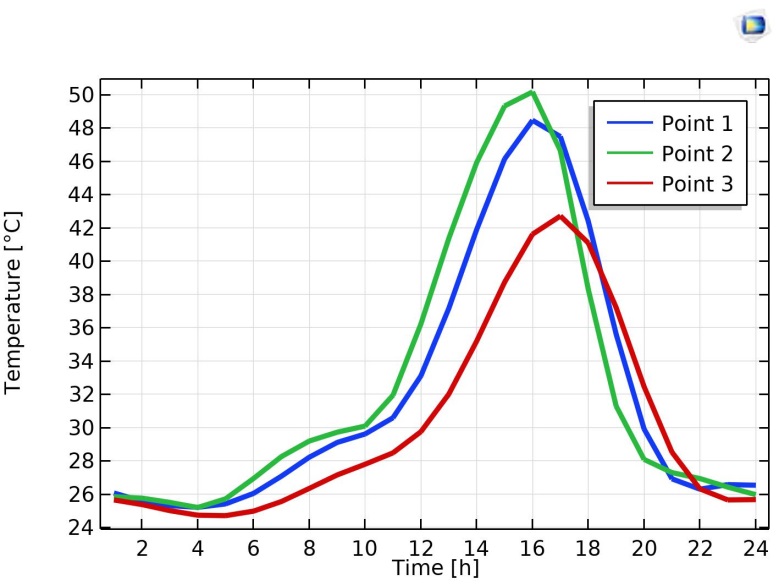**  **Azimuth 90°** |
| --- | --- |
| **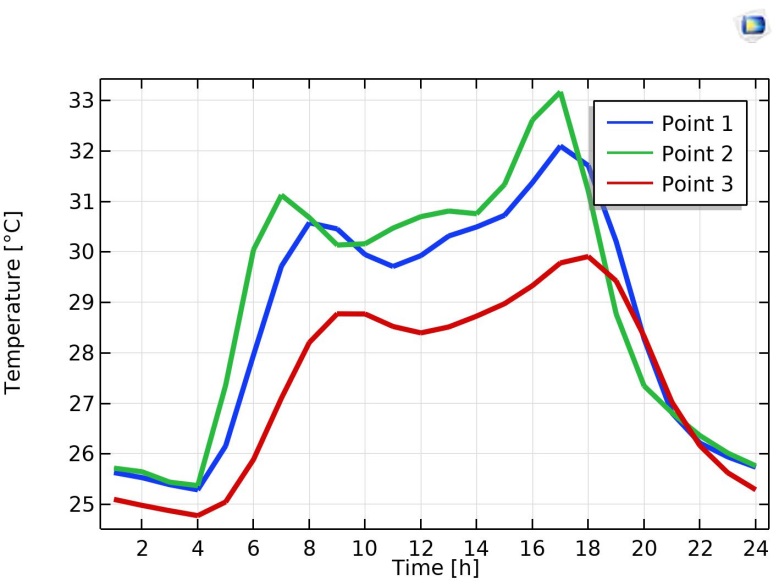**  **Azimuth 180°** | 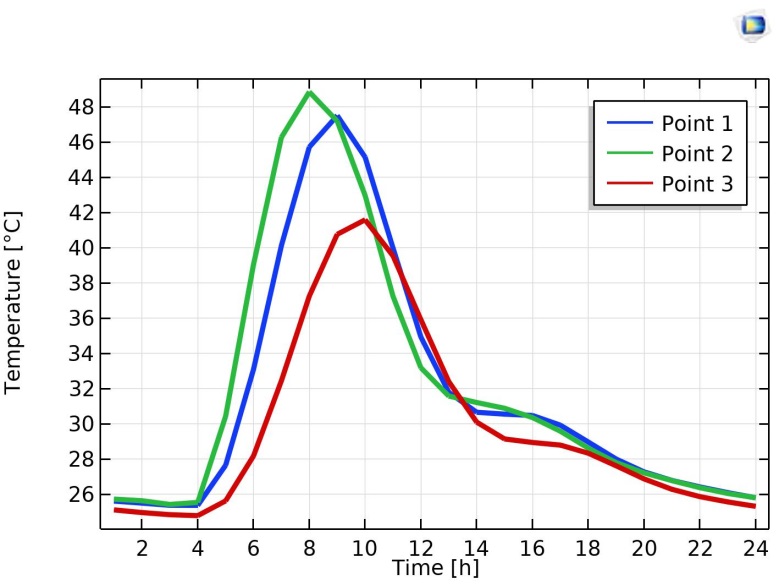  **Azimuth 270°** |

**Figure B12.** Hourly building element temperature graphs (°C) for geometry 3 of novel double-skin façade (DSF) controlled-temperature building element – Autumn

| **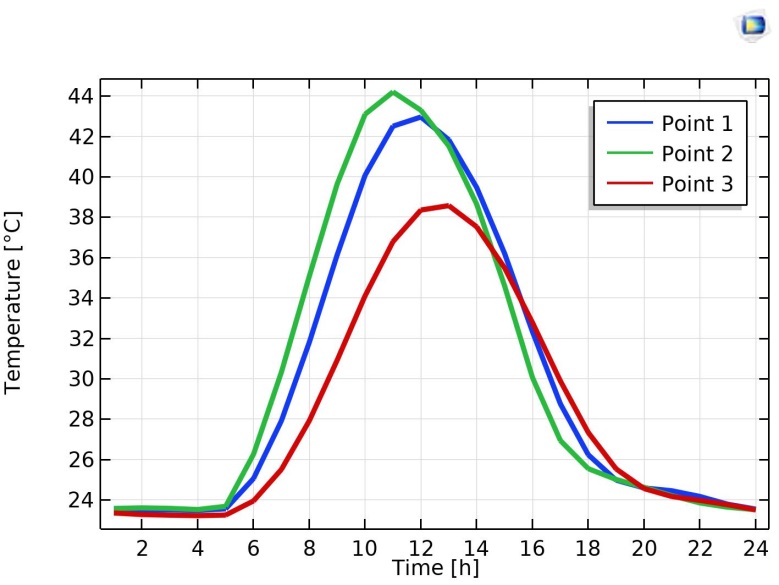**  **Azimuth 0°** | **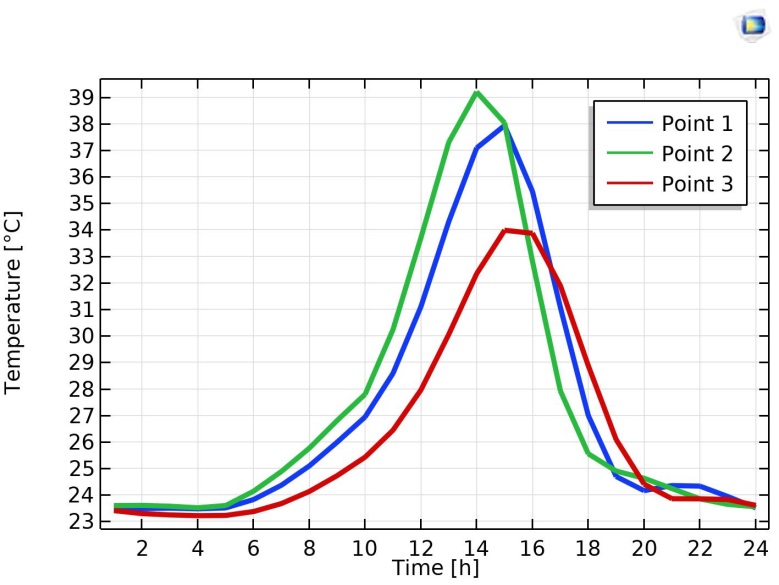**  **Azimuth 90°** |
| --- | --- |
| **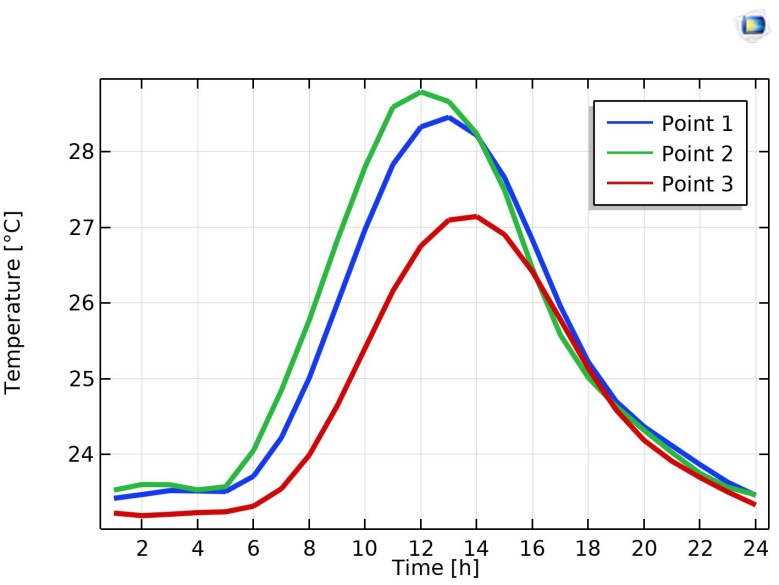**  **Azimuth 180°** | 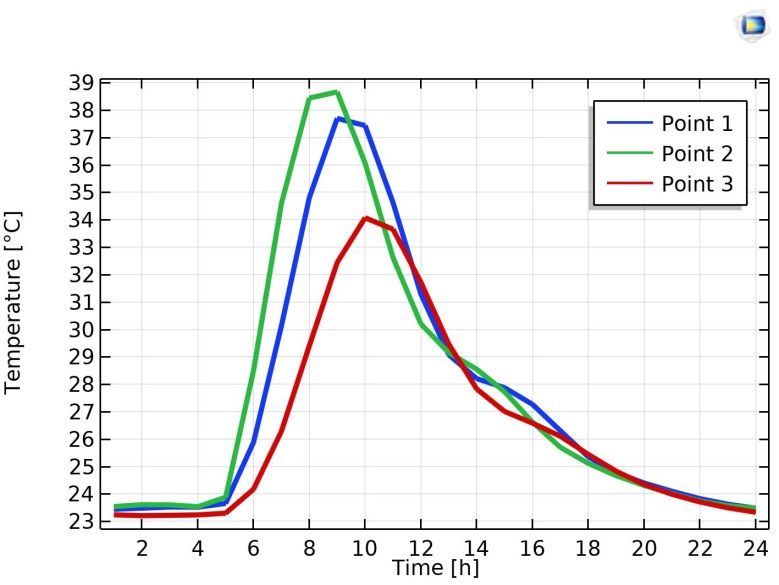  **Azimuth 270°** |

**Figure B13.** Hourly building element temperature graphs (°C) for geometry 4 of novel double-skin façade (DSF) controlled-temperature building element – Winter

| **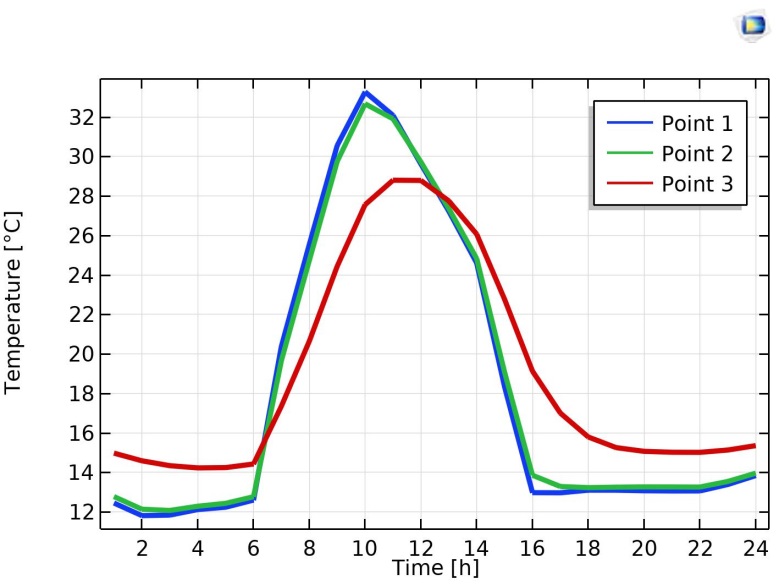**  **Azimuth 0°** | **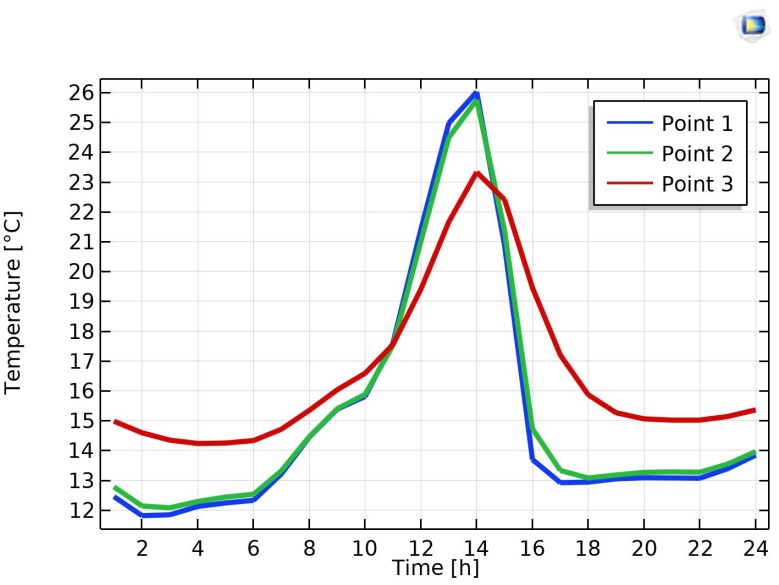**  **Azimuth 90°** |
| --- | --- |
| **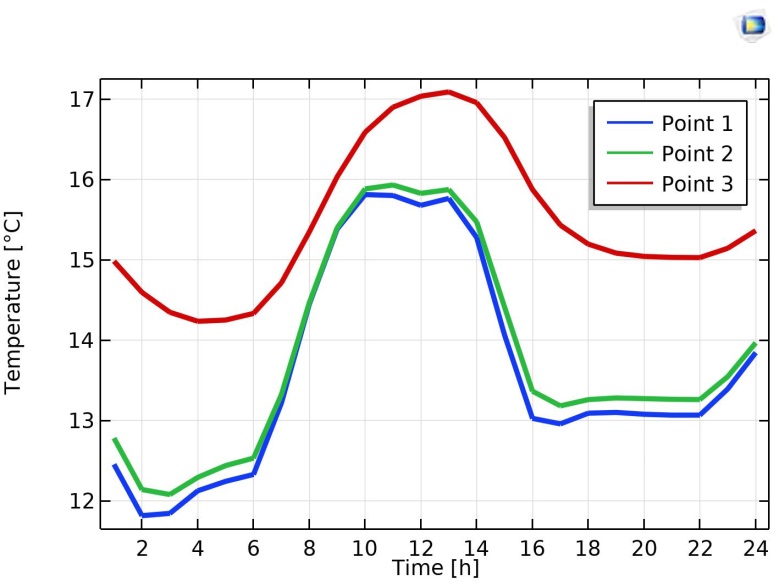**  **Azimuth 180°** | 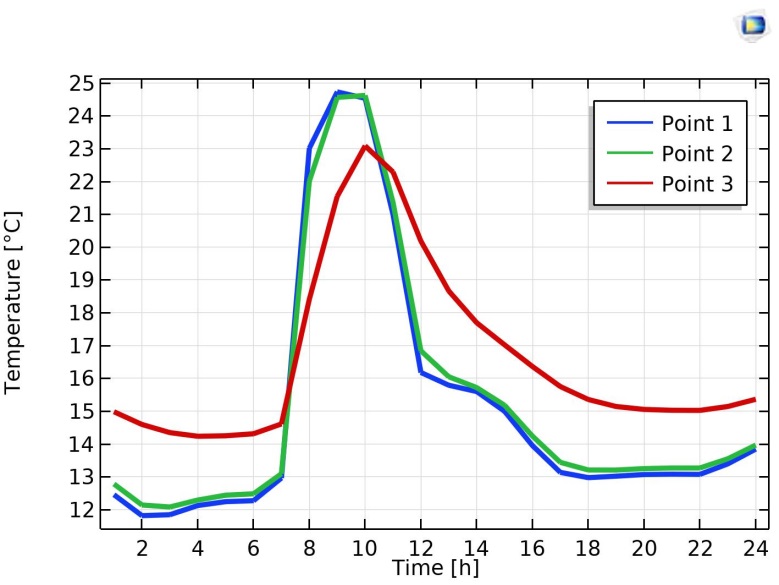  **Azimuth 270°** |

**Figure B14**. Hourly building element temperature graphs (°C) for geometry 4 of novel double-skin façade (DSF) controlled-temperature building element – Spring

| **Azimuth 0°** | **Azimuth 90°** |
| --- | --- |
| **Azimuth 180°** | **Azimuth 270°** |

**Figure B15**. Hourly building element temperature graphs (°C) for geometry 4 of novel double-skin façade (DSF) controlled-temperature building element – Summer

| **Azimuth 0°** | **Azimuth 90°** |
| --- | --- |
| **Azimuth 180°** | **Azimuth 270°** |

**Figure B16**. Hourly building element temperature graphs (°C) for geometry 4 of novel double-skin façade (DSF) controlled-temperature building element – Autumn

| **Azimuth 0°** | **Azimuth 90°** |
| --- | --- |
| **Azimuth 180°** | **Azimuth 270°** |

**Figure B17**. Hourly building element temperature graphs (°C) for geometry 5 of novel double-skin façade (DSF) controlled-temperature building element – Winter

| **Azimuth 0°** | **Azimuth 90°** |
| --- | --- |
| **Azimuth 180°** | **Azimuth 270°** |

**Figure B18**. Hourly building element temperature graphs (°C) for geometry 5 of novel double-skin façade (DSF) controlled-temperature building element – Spring

| **Azimuth 0°** | **Azimuth 90°** |
| --- | --- |
| **Azimuth 180°** | **Azimuth 270°** |

**Figure B19**. Hourly building element temperature graphs (°C) for geometry 5 of novel double-skin façade (DSF) controlled-temperature building element – Summer

| **Azimuth 0°** | **Azimuth 90°** |
| --- | --- |
| **Azimuth 180°** | **Azimuth 270°** |

**Figure B20**. Hourly building element temperature graphs (°C) for geometry 5 of novel double-skin façade (DSF) controlled-temperature building element – Autumn

| **Azimuth 0°** | **Azimuth 90°** |
| --- | --- |
| **Azimuth 180°** | **Azimuth 270°** |

**Figure B21.** Hourly building element temperature graphs (°C) for geometry 6 of novel double-skin façade (DSF) controlled-temperature building element – Winter

| **Azimuth 0°** | **Azimuth 90°** |
| --- | --- |
| **Azimuth 180°** | **Azimuth 270°** |

**Figure B22**. Hourly building element temperature graphs (°C) for geometry 6 of novel double-skin façade (DSF) controlled-temperature building element – Spring

| **Azimuth 0°** | **Azimuth 90°** |
| --- | --- |
| **Azimuth 180°** | **Azimuth 270°** |

**Figure B23**. Hourly building element temperature graphs (°C) for geometry 6 of novel double-skin façade (DSF) controlled-temperature building element – Summer

| **Azimuth 0°** | **Azimuth 90°** |
| --- | --- |
| **Azimuth 180°** | **Azimuth 270°** |

**Figure B24**. Hourly building element temperature graphs (°C) for geometry 6 of novel double-skin façade (DSF) controlled-temperature building element – Autumn

| **Azimuth 0°** | **Azimuth 90°** |
| --- | --- |
| **Azimuth 180°** | **Azimuth 270°** |
